# Genetically and Phenotypically Distinct *Pseudomonas aeruginosa* Cystic Fibrosis Isolates Share a Core Proteomic Signature

**DOI:** 10.1371/journal.pone.0138527

**Published:** 2015-10-02

**Authors:** Anahit Penesyan, Sheemal S. Kumar, Karthik Kamath, Abdulrahman M. Shathili, Vignesh Venkatakrishnan, Christoph Krisp, Nicolle H. Packer, Mark P. Molloy, Ian T. Paulsen

**Affiliations:** 1 Department of Chemistry and Biomolecular Sciences, Faculty of Science and Engineering, Macquarie University, Sydney, New South Wales, Australia; 2 Australian Proteome Analysis Facility, Macquarie University, Sydney, New South Wales, Australia; Ghent University, BELGIUM

## Abstract

The opportunistic pathogen *Pseudomonas aeruginosa* is among the main colonizers of the lungs of cystic fibrosis (CF) patients. We have isolated and sequenced several *P*. *aeruginosa* isolates from the sputum of CF patients and compared them with each other and with the model strain PAO1. Phenotypic analysis of CF isolates showed significant variability in colonization and virulence-related traits suggesting different strategies for adaptation to the CF lung. Genomic analysis indicated these strains shared a large set of core genes with the standard laboratory strain PAO1, and identified the genetic basis for some of the observed phenotypic differences. Proteomics revealed that in a conventional laboratory medium PAO1 expressed 827 proteins that were absent in the CF isolates while the CF isolates shared a distinctive signature set of 703 proteins not detected in PAO1. PAO1 expressed many transporters for the uptake of organic nutrients and relatively few biosynthetic pathways. Conversely, the CF isolates expressed a narrower range of transporters and a broader set of metabolic pathways for the biosynthesis of amino acids, carbohydrates, nucleotides and polyamines. The proteomic data suggests that in a common laboratory medium PAO1 may transport a diverse set of “ready-made” nutrients from the rich medium, whereas the CF isolates may only utilize a limited number of nutrients from the medium relying mainly on their own metabolism for synthesis of essential nutrients. These variations indicate significant differences between the metabolism and physiology of *P*. *aeruginos*a CF isolates and PAO1 that cannot be detected at the genome level alone. The widening gap between the increasing genomic data and the lack of phenotypic data means that researchers are increasingly reliant on extrapolating from genomic comparisons using experimentally characterized model organisms such as PAO1. While comparative genomics can provide valuable information, our data suggests that such extrapolations may be fraught with peril.

## Introduction

Cystic fibrosis (CF) is an autosomal recessive genetic disorder affecting most critically the lungs, and also the pancreas, liver, and intestine. It is characterized by abnormal transport of chloride and sodium ions across the epithelium leading to thick, viscous secretions. It is most common among the Caucasian population where, according to the World Health Organisation, approximately 1 in 2500 children are born with the genetic mutation leading to the development of CF. CF is caused by mutations in the gene encoding for the CF transmembrane conductance regulator protein (CFTR) involved in the regulation of the movement of chloride and sodium ions across epithelial membranes. The thick mucus that forms as a result of *CFTR* mutations represents a breeding ground for various microorganisms that cause chronic infection in lungs, thus leading to complications associated with the disease.

*Pseudomonas aeruginosa*, a Gram-negative opportunistic human pathogen, is one of the main colonizers of lungs in CF patients. It was found that by the age of 3 years, over 95% of children with CF show evidence of intermittent *P*. *aeruginosa* infection [1]. Moreover, early colonization with *P*. *aeruginosa* has been strongly correlated with poor prognosis in CF [2, 3]. By the age of 25, according to the 2010 Cystic Fibrosis Foundation Patient registry Annual Data Report, *P*. *aeruginosa* becomes the most dominant microorganism in the respiratory tract of CF patients.

Due to its metabolic versatility, innate resistance to the majority of drugs used in clinical practice, and extensive biofilm formation, infections caused by *P*. *aeruginosa* are especially hard to treat using conventional treatment regimes and are often destined to fail. As a result, CF is ranked among the most widespread life-shortening genetic diseases with the current life expectancy often not exceeding mid 40s.

Successful CF pathogens, including *P*. *aeruginosa*, have developed an effective arsenal to establish infection and evade the host response, together with an ability to adapt readily to the lung environment [4]. Thus, according to reports, isolates of *P*. *aeruginosa* that are involved in the acute infection and initial colonisation of CF lungs in early childhood differ from those found in adults with established chronic infections, the latter often showing adaptations specific to the CF lung environment [5, 6].

**Fig 1:**
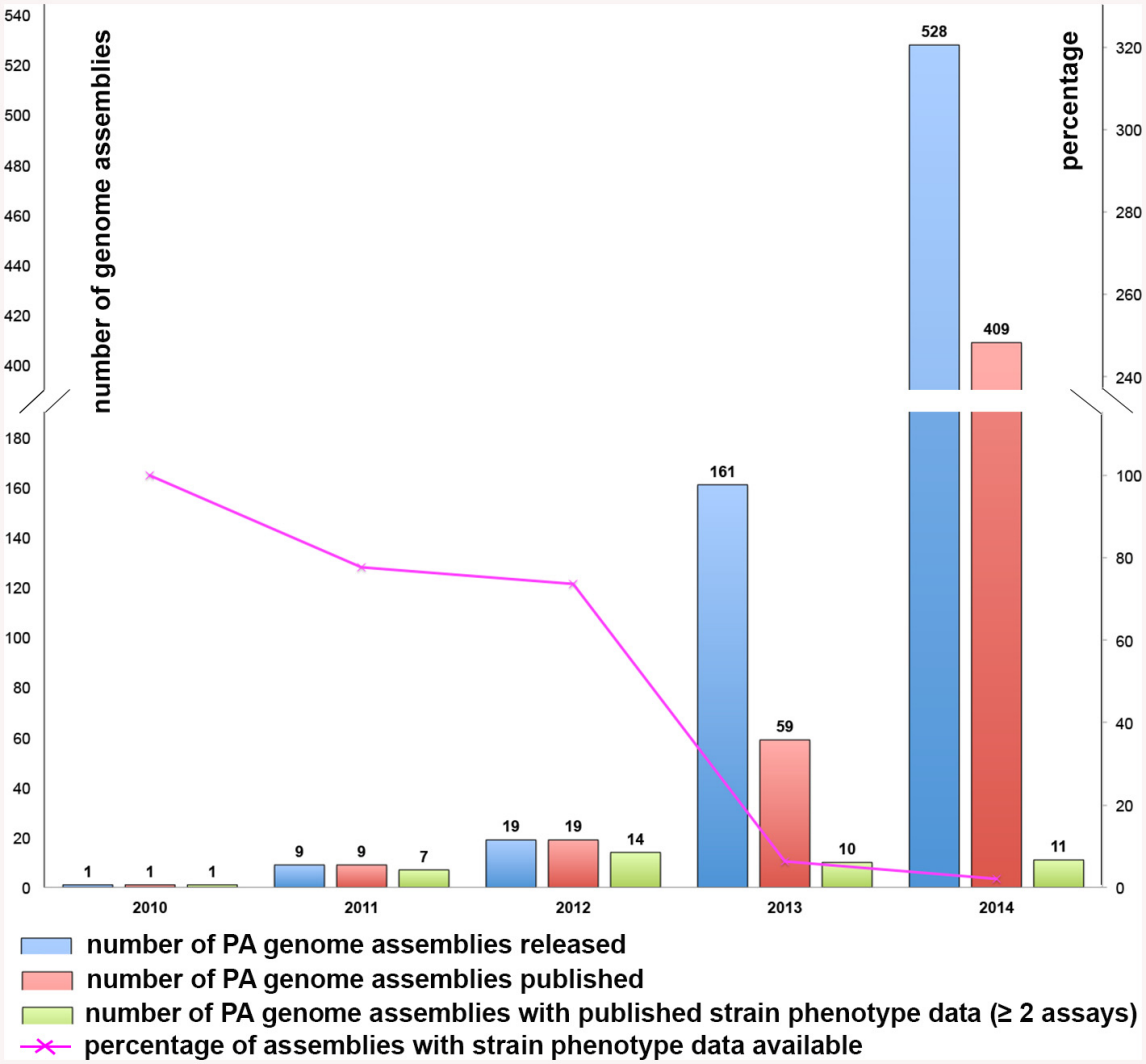
Data on *P*. *aeruginosa* (PA) genome assemblies released in the last 5 years (2010–2014 inclusive).

Considering its role in the disease progression, it is not surprising that *P*. *aeruginosa* has become a focal point for research in CF and other biofilm related complications. Improvements in DNA sequencing technology have led to the sequencing of hundreds of *P*. *aeruginosa* genomes in recent years, many of them from CF patients. However, for the vast majority of these strains there is little or no published experimental data on their phenotypic features (Fig 1). Instead most phenotypic experimental work on *P*. *aeruginosa* has focused on common well-characterised model strains, such as PAO1. Our knowledge of these sequenced CF isolates is thus largely based on extrapolations from model strains via *in silico* genome comparisons.

Strain PAO1 is a widely used model organism and the first *Pseudomonas* species to have its genome sequenced [7]. In 2014 alone 155 articles were published, as listed on NCBI PubMed, using PAO1 as a model *P*. *aeruginosa* strain, and 15% of those articles made direct correlations to CF. As strain PAO1 does not have a CF origin but was isolated from a wound infection in 1955 [8] and has been maintained in laboratories worldwide since then, PAO1 may have questionable relevance to CF.

In this study we have obtained fresh isolates of *P*. *aeruginosa* from the sputum of CF patients, subsequently named PASS1-4, and compared them with each other and with the model laboratory strain PAO1. The study aimed to identify specific adaptations developed by *P*. *aeruginosa* during chronic infection of CF lungs with an overarching aim of better understanding the mechanisms that render *P*. *aeruginosa* a successful CF lung colonizer. Significant differences were observed in genomes and phenomes amongst the CF isolates reflecting varying adaptation paths. At the same time, the CF isolates shared a common proteomic signature that was remarkably distinct from the proteome of PAO1.

Our study highlights the limitations of using model organisms when examining the role of bacteria in the context of their natural host/environment. Isolates from hosts/environments of interest may have developed specific adaptations that may not be present in model strains, or may have been lost during prolonged cultivation. Understanding these adaptations would be crucial for the successful treatment of infections caused by pathogens; therefore, the use of freshly obtained isolates is an important adjunct to work on model strains.

## Materials and Methods

### Strains used in this study

*P*. *aeruginosa* isolates PASS1-4 were previously obtained from the sputum of adult CF patients: PASS1 was obtained from a 40-year old female patient, PASS2 –from a 27-year old male, PASS3 –from a 23 year old male and PASS4 –from a 23-year old female [9]. Strains PASS1-4, as well as *P*. *aeruginosa* PAO1, were maintained in stock in -80°C freezer and grown on Luria Bertani (LB) solid or liquid media whenever needed unless otherwise stated, allowing minimal passaging before the assays.

### Genome sequencing, assembly and comparative genomics

DNA was isolated from *P*. *aeruginosa* strains using the Invitrogen PureLink Genomic DNA kit and sequenced at the Ramaciotti Centre for Gene Function Analysis (UNSW, Sydney) on an Illumina HiSeq 2000 platform. Sequence data was assessed for quality using FastQC (Babraham Bioinformatics) and assembled *de novo* using the VELVET algorithm [10]. Genome assembly quality and statistics were further evaluated using QUAST [11].

Genome sequencing data for strains PASS1-4 have been deposited to the NCBI Whole Genome Shotgun (WGS) database and are accessible under BioProject ID numbers PRJNA295120, PRJNA295121, PRJNA295122 and PRJNA295123 respectively (http://www.ncbi.nlm.nih.gov/bioproject).

The phylogenetic relationship of PASS1-4 strains to other *P*. *aeruginosa* strains in the MLST database (http://pubmlst.org/paeruginosa) was inferred via maximum likelihood analysis using the Arb software package [12]. The phylogenetic analysis was based on concatenated nucleotide sequences of seven genes utilized in the multilocus sequence typing (MLST) of *P*. *aeruginosa*. These included *acsA* (acetyl coenzyme A synthetase), *aroE* (shikimate dehydrogenase), *guaA* (GMP synthase), *mutL* (DNA mismatch repair protein), *nuoD* (NADH dehydrogenase I chain C, D), *ppsA* (phosphoenolpyruvate synthase), *trpE* (anthralite synthetase component I). Further analysis has been performed by assigning a number to each distinct allele within a locus according to the number available in the *P*. *aeruginosa* MLST database. As a result, each isolate was given up to seven numbers that represented its strain type. Any strain type that did not have a match in the existing database was designated as a “new” type.

Multiple genome alignments were performed using BRIG [13] and MAUVE [14] software.

Reciprocal similarity searches of all predicted and expressed proteins were performed using BLAST algorithm [15], as well as Proteinortho5 tool [16] followed by the visualization of results using FriPan (http://www.vicbioinformatics.com/software.fripan.shtml).

### Carbon source utilization

Carbon source utilization was assessed by growing bacteria in M9 minimal medium (Sigma) with the addition of the substrate of interest as a sole carbon source, in biological triplicates. Cultures were incubated on a horizontal shaker for 48 hours at 37°C after which the growth was assessed.

### Biofilm formation in flow cells

To assess formation of biofilms by *P*. *aeruginosa*, strains PASS1-4 and PAO1 were grown in continuous flow-cell system [17] in LB medium at 37°C for 48 hours and biofilms formed on the surface of coverslips were imaged using Olympus FV1000 Laser Scanning Confocal Microscopy (LSCM) System after staining with the BacLight Live/Dead stain (Molecular Probes). Three-dimensional rendering of LSCM images and the subsequent quantification were performed using the Imaris software (Bitplane). Quadruplicate images were used in the quantitative assessment of LSCM images of each sample; these included two independent flow cell chambers that were inoculated from separate culture stocks, and two distant fields of view in each chamber.

### Binding of *Pseudomonas aeruginosa* to mucin

*P*. *aeruginosa* isolates were cultured in 10 ml of LB broth at 37°C for 8 hours with shaking at 185 rpm. Bacterial cells were pelleted by centrifugation at 3000 x *g* for 5 min, washed twice with phosphate buffered saline (PBS), and then fluorescently labelled by resuspension in 1 ml of PBS with SYBR^®^ Green (0.1% w/v, Sigma) for 3 min. Labelled cells were collected by centrifugation at 3000 x g for 3 mins, and then washed thrice in PBS to remove residual dye. Meanwhile, PVDF membranes were placed into the wells of a 96-well microtiter plate and activated by soaking in methanol followed by washing three times in PBS. Fifty microliters of 1 mg/ml porcine gastric mucin (PGM, Sigma) were added into each well containing a PVDF membrane. Bacteria were resuspended in PBS to an OD_600_ = 1.0, applied to the wells of 96-well microplate containing the mucin-coated PVDF membranes and incubated for 30 minutes while slowly shaking at 100 rpm at RT. Unbound bacteria were washed off the membrane three times with PBS and the attached bacteria were fluorescently measured (Ex 485nm, Em 520nm) using a Fluorostar Galaxy plate reader (BMG Labtech, Offenburg, Germany). Wells containing immobilised mucin (no bacteria applied) were used as a negative control. The binding to the mucin by the different bacterial strains was normalized against the maximum bacterial binding measured on each plate.

### Flagella mediated motility

Flagella mediated motility was assayed by point inoculation of LB plates containing 0.3% (w/v) agar as previously described [18]; zone sizes were observed after 48 hours of incubation at 37°C. The assay was performed in biological triplicates.

### Virulence against the nematode eukaryotic model *Caenorhabditis elegans*

To assess toxicity against the nematode *C*. *elegans*, a selective grazing assay was performed as previously described [19]. Briefly, all strains of *P*. *aeruginosa* were spotted from frozen stock cultures on a single LB agar plate, followed by incubation at 37°C for 4 days. At day 4, 5 μl of M9 medium with four to five L4-stage nematodes were added next to each colony. The plates were stored at room temperature and checked daily. A strain was considered positive if the colony was not grazed after 14 days of incubation with *C*. *elegans*, across all three biological replicates. The assay was performed in three biological replicates.

### Assessment of phenazine production

Phenazine compounds were extracted from *P*. *aeruginosa* cultures (in biological triplicates) after 40 hours of growth in LB broth, at 37°C with shaking. Two millilitres of chloroform were added to 5 ml of culture and incubated with shaking for 20 minutes. Chloroform fractions were collected, dried under reduced pressure and dissolved in 80% v/v acetonitrile in 25 mM ammonium acetate. Filtered samples were applied to the Zorbax Eclipse Plus C18 Rapid Resolution column (Agilent, 2.1 x 50 mm, 1.8 micron) and analysed using the Infinity 1290 Ultra High Performace Liquid Chromatography (UHPLC) instrument equipped with the Infinity 1290 photodiode array detector (Agilent). The separation program was adopted for UHPLC based on the previously published protocol [20]. The solvent flow rate was 0.5 ml/min and consisted of 0.25 minutes of 8% v/v acetonitrile–25 mM ammonium acetate, followed by a 3-min linear gradient to 80% v/v acetonitrile–25 mM ammonium acetate. UHPLC gradient profiles were monitored at spectral peak maxima of 257.0 and 313.0 nm.

### Pyoverdine production

Pyoverdine production was assessed by growing strains on iron-limited King’s medium at 37°C for 40 hours, in biological triplicates, followed by visualization of pyoverdine under UV light, as previously described [21].

### Protein extraction for proteomics

Overnight cultures of *P*. *aeruginosa* strains (PAO1, PASS1, PASS2, PASS3 and PASS4) in LB broth, in biological triplicates, were inoculated into fresh LB broth and grown to mid-logarithmic phase with incubation at 37°C and shaking at 200 rpm. Cells were collected by centrifugation at 2500 x *g* for 10 min at 4°C and washed thrice with PBS, pH 7.4. The cell pellet (~ 0.5g) was weighed and resuspended in 0.5ml of PBS (pH 7.4) containing complete protease inhibitor cocktail (EDTA-free, Roche), benzonase (1:100 v/v, Sigma) and equal amounts of acid washed glass beads (Sigma Aldrich). Cells were lysed by bead-beating thrice at 4.5 Throw for 20 seconds using a FastPrep FP120 bead-beater apparatus (Savant) with 10 min breaks on ice. Cell debris and unbroken cells were removed by centrifugation at 2500 x *g* for 8 min at 4°C. Proteins in the supernatant were precipitated by the addition of ice-cold acetone in the ratio of 1:9 (v/v) and incubated overnight at -20°C followed by centrifugation at 2500 x *g* for 10 min at 4°C. The pellet was washed twice and resuspended in 1% (w/v) SDS in deionized water. Total protein content was measured using Pierce BCA Protein Assay Kit (Thermo Scientific).

### 1D SDS-PAGE and in-gel digestion

Thirty micrograms of protein from each sample was diluted using NuPAGE Laemmli loading buffer (Life Technologies) containing 50 mM DTT and denatured at 95°C for 5 min. Samples were spun down and loaded into a NuPAGE (4–12% T Bis-Tris) precast ready gel. Electrophoresis was performed using NuPAGE MOPS SDS running buffer to run the sample 10 mm into the gel. After electrophoresis the gels were stained with colloidal Coomassie blue (Sigma Aldrich) and the band was cut into 1 mm^3^ cubes and transferred into 1.5 ml microcentrifuge tubes to perform in-gel tryptic digestion.

Trypsin in-gel digestion and peptide extraction were performed as previously described [22]. Briefly, gel bands were destained using acetonitrile (ACN) and 100 mM ammonium bicarbonate (ABC) in the 1:1 ratio, followed by reduction with 10 mM DTT prepared in 100 mM ABC at 56°C for 30 min and alkylation with 55 mM iodoacetamide prepared in 100 mM ABC for 20 min in the dark. The gel plugs were washed twice with ACN, with 5 minutes of incubation during each wash. The gel plugs were subsequently dried in a vacuum centrifuge, and digested overnight with sequencing-grade modified trypsin (Promega) in the ratio 1:30 at 37°C. Products of digestion were collected in 1.5 ml microcentrifuge tubes and combined with extracts from a consecutive extraction with ACN and 5% (v/v) formic acid at 37°C for 30 min. The total extract was then concentrated in a vacuum centrifuge and reconstituted with loading buffer (2% v/v ACN, 0.1% v/v FA) for mass spectrometry.

### Mass spectrometry

Peptides were analysed using a data-independent acquisition method known as SWATH-MS [23]. Five spectral ion libraries were generated, one for each strain by pooling biological replicates and separating the peptides into five salt fractions by online strong cation exchange chromatography (SCX) These libraries were later used for proteomics data analysis. Fractionated samples were analysed using a nanoLC ultra 2D cHiPLC system (Eksigent, part of SCIEX) in conjunction with a TripleTOF® 5600 (ABSciex) using positive nanoflow electrospray analysis and an information-dependent acquisition (IDA) mode. In data dependent MS/MS acquisition 20 most intense m/z values exceeding a threshold > 150 counts per second (cps) with charge stages between 2+ and 4+ were selected for analysis following a full MS survey scan and excluded for 20 sec to minimize redundant precursor sampling.

For SWATH-MS, each biological triplicate was analyzed within a 60 min increasing ACN RP gradient (5% to 45% using 90% v/v ACN 0.1% v/v FA) using 60 variable window m/z ranges (400–1250 m/z) selected based on intensity distribution of precursor m/z in the IDA data sets. Collision energies were calculated for 2+ precursors with m/z values of lowest m/z in window + 5 m/z and a collision energy spread of 5 eV was used.

### Protein identification

Spectral libraries for SWATH-MS quantitation were generated with ProteinPilot^TM^ software 4.2 using the Paragon^TM^ algorithm (ABSciex) thorough ID mode including biological modifications [23]. MS/MS data were searched against the *Pseudomonas aeruginosa* strain PAO1 protein sequence database retrieved from GenBank (January 2013) and Pseudomonas Genome Database (www.pseudomonas.com) [24] and *in-silico* translated genome databases of PASS1-4 strains. Carbamidomethylation of Cys residues was selected as a fixed modification. An Unused Score cut-off was set to 2.0 (99% confidence), equivalent to a protein false discovery rate (FDR) < 1%.

### Proteomic data analysis

Generated protein libraries for each strain were imported into PeakView^TM^ software 2.1 using the SWATH MicroApp 2.0 (release 27 November 2013) and matched against SWATH-MS data for each individual replicate. After retention time calibration with endogenous peptides, data were processed using the following settings; a maximum of 100 peptides per protein, maximal 6 transitions per peptide, peptide confidence threshold of 60%, transition FDR < 1%, 10 min extraction window and fragment extraction tolerance of 75 ppm.

Expressed proteins were determined by comparing summed protein areas from extracted ion chromatograms between PAO1 and PASS1-4 strains to identify those with a +/- 5-fold change and ANOVA p < 0.01.

The mass spectrometry proteomics data have been deposited to the ProteomeXchange Consortium via the PRIDE partner repository [25] with the dataset identifier PXD002865.

In order to reveal similarities and differences in the proteomics profiles of *P*. *aeruginosa* strains tested, the reciprocal Blast searches and orthologue analyses were performed as was previously described for the predicted proteome.

Proteins identified as being expressed by *P*. *aeruginosa* strains during the growth in LB medium were mapped onto the *P*. *aeruginosa* PAO1 curated database in BioCyc [26] and metabolic pathways visualized using the Pathway Tools 17.5 cellular overview diagram and Omics Viewer [27].

## Results and Discussion

### Colony morphology of isolated CF strains

Four *P*. *aeruginosa* isolates were obtained from the sputum of CF patients at the Westmead Hospital (Sydney, Australia) and were minimally passaged. Isolates displayed significant diversity in their colony morphology: PASS1 formed pale-green non-mucoid colonies when grown on LB agar, PASS2 –light brown non-mucoid colonies, PASS3 –mainly white mucoid colonies and PASS4 –dark green-blue non-mucoid colonies.

### Whole genome sequencing and phylogeny

**Fig 2:**
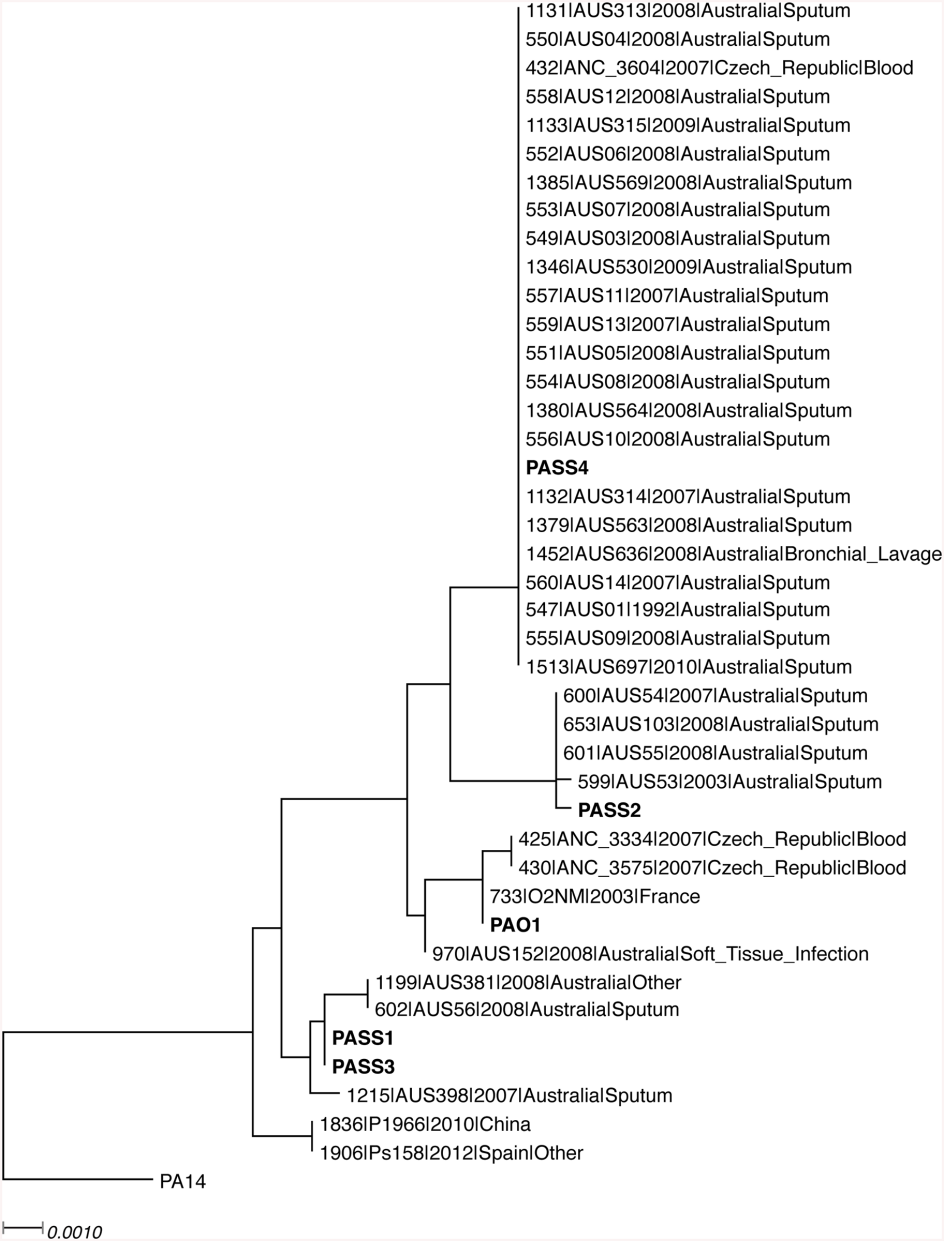
The maximum likelihood phylogenetic tree generated using concatenated sequences of MLST genes in *P*. *aeruginosa*. Isolates mentioned in this study are shown in bold; all the other closely related MLST sequences are retrieved from *P*. *aeruginosa* PubMLST database (http://pubmlst.org/paeruginosa). *P*. *aeruginosa* PA14 is used as an outgroup. The scale bar indicates the number of substitutions per nucleotide position.

*De novo* assembly of the sequencing reads obtained from Illumina HiSeq platform yielded draft genomes as per Table 1. Strain PASS3 was found to have the largest genome, and PASS2 genome was the smallest. Based on the MLST phylogeny, strains PASS1 and PASS3 were found to have an identical MLST sequence, while PASS2 and PASS4 were in different MLST clusters distinct from PASS1, PASS3 and PAO1 (Fig 2). Consistent with their isolation source, all CF isolates (PASS1-4) clustered with other Australian sputum isolates, while PAO1 was closely related to a more diverse set of isolates from various sources, including tissue infection strains.

**Table 1:** Genome assembly statistics for PASS1-4 strains.

Strain	Number of contigs (> 1 kb)	N50	Genome size (Mbp)	Number of ORFs
PASS1	81	205938	6.3	5792
PASS2	96	186035	6.1	5795
PASS3	76	255936	6.4	5847
PASS4	93	171309	6.3	5936

Further MLST analysis revealed that strains PASS1-3 do not have an exact match in the MLST database (http://pubmlst.org/paeruginosa), and therefore represent new strain types, while PASS4 belongs to the strain type 649 (S1 Table). The latter currently comprises of 23 isolates one of which originates from the blood sample in Czech Republic and the rest are epidemic isolates obtained from lungs of CF patients in Australia.

### Comparative genomics and the analysis of predicted proteins

**Fig 3:**
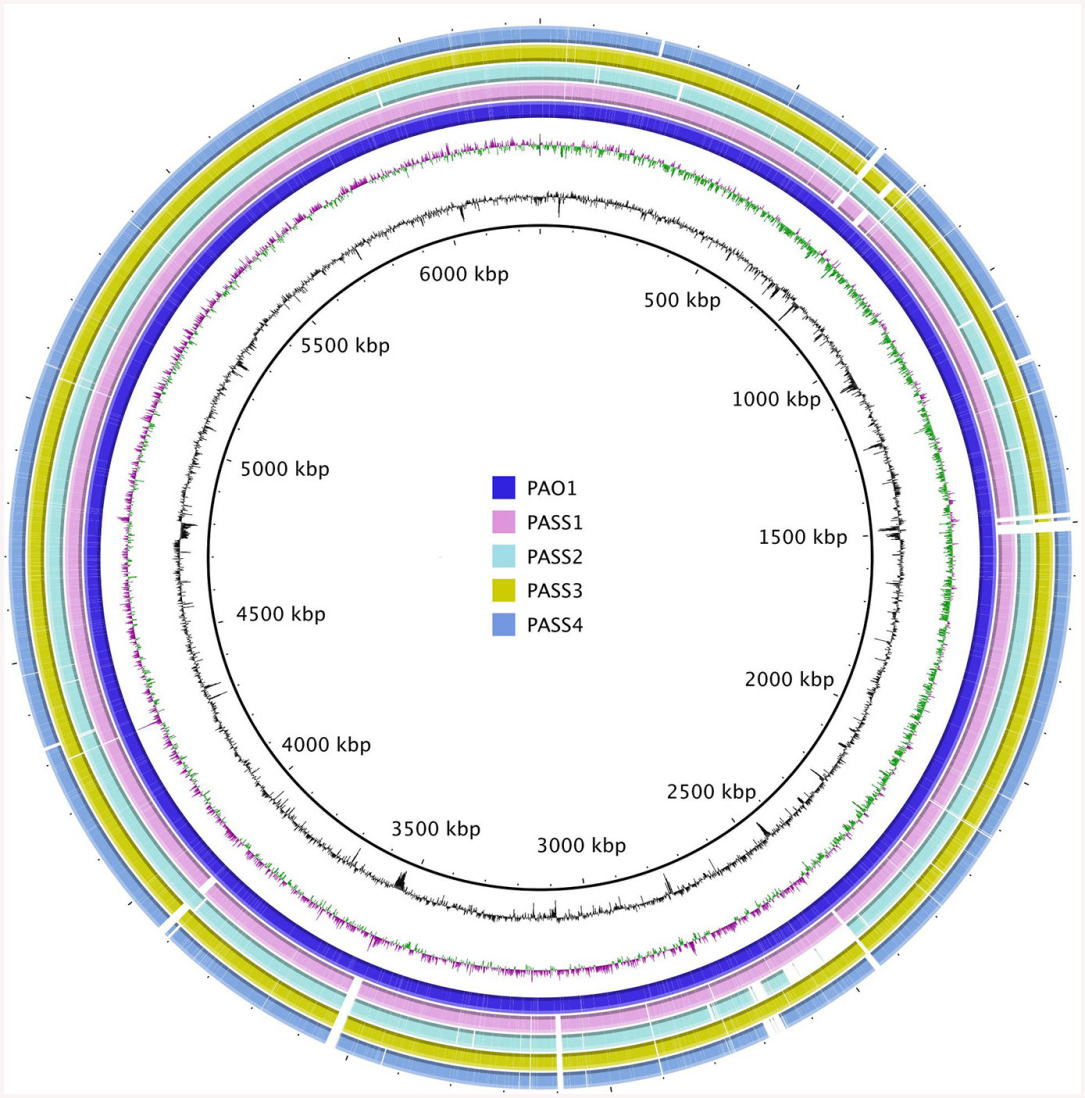
Circular representation of *P*. *aeruginosa* PASS1-4 draft genomes aligned against the reference genome of PAO1, generated using BRIG software. The two innermost circles represent the GC Content and the GC Skew respectively.

Multiple genome alignments using BRIG [13] and MAUVE [14] tools show extensive gene conservation and synteny between PAO1 and the PASS1-4 genomes (Fig 3). The analysis of strain PASS2 draft genome also revealed an absence of a ~ 160 kB genomic region corresponding to the nucleotide positions between 2439150 and 2602350 in the PAO1 genome and containing genes with locus tags PA2218-PA2354 (Fig 3). This region, absent in PASS2, contains a number of potential virulence and colonization determinants, including the *psl* cluster of genes responsible for the production of biofilm matrix component Psl, as well as genes encoding chitinase and those involved in biosynthesis of L-2-amino-4-methoxy-trans-3-butenoic acid (AMB) toxin (Fig 3). This may represent an adaptation to the CF lung via genome reduction and the loss of several virulence factors. Decreased virulence has been previously described among the characteristics of *P*. *aeruginosa* isolates from chronic lung infections as a way of minimizing the host immune response and limiting bacterial resource expenditure [28]. A similar large deletion of ~ 180 kB spanning from PA2272 to PA2410 has been previously reported in a different CF isolate [29].

**Fig 4:**
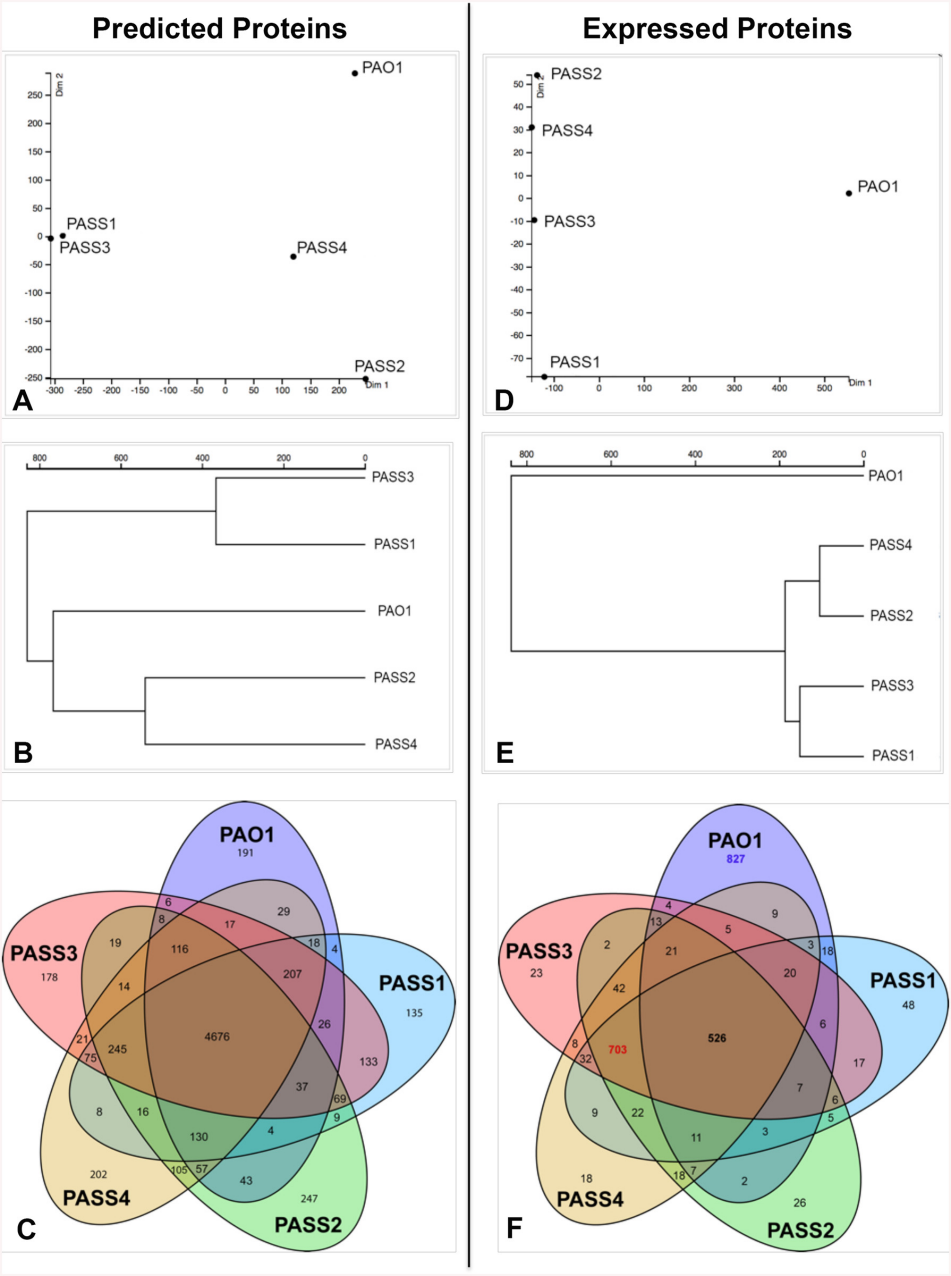
Comparison of predicted and expressed proteins in CF isolates PASS1-4 and the reference strain PAO1. The 2-dimensional plot (A) and clustering representation (B) of all predicted proteins of corresponding strains analysed via Proteinortho and FriPan software; (C)–Venn diagram representing the results of reciprocal BLAST searches of all predicted proteins in corresponding strains. The 2-dimensional plot (D) and clustering representation (E) of all expressed proteins of corresponding strains as assessed by proteomics and analysed via Proteinortho and FriPan software; (F)–Venn diagram representing the results of reciprocal BLAST searches of expressed proteins detected via proteomics; the number of proteins uniquely expressed by PAO1 is shown in blue, the number of proteins shared by PASS1-4 and not expressed by PAO1 is in red.

BLAST searches of predicted proteins of PASS1-4 strains and the standard laboratory strain PAO1 revealed 4676 shared protein sequences between all 5 strains (Fig 4C). Ortholog analysis of all predicted proteins performed using Proteinortho5, followed by visualization of results via FriPan demonstrated distinct clustering of PAO1 at a significant distance from CF isolates PASS1-4. Interestingly, significant differences were also observed among PASS1-4 strains, *i*.*e*. PASS1 and PASS3 showed high degree of similarity, while PASS2 and PASS4 clustered together with a lower level of similarity (Fig 4A and 4B), in agreement with the MLST phylogeny (Fig 2).

### Carbon source utilization

Genomic analysis revealed that PASS2 lacks genes essential for the utilization of various carbon sources. Many of these genes were located in the ~160 kB region of DNA absent in strain PASS2. These include genes involved in transport and catabolism of mannitol (PA2337-PA2344); the *bkd* operon for utilization of valine, leucine and isoleucine; and genes involved in the utilization of gluconate and glycerol (PA2321-2322 and PA2352 respectively). Minimal media growth experiments confirmed that PASS2 was unable to grow in the minimal medium in the presence of the above-mentioned compounds as sole carbon sources (data not shown).

### Biofilm formation

**Fig 5:**
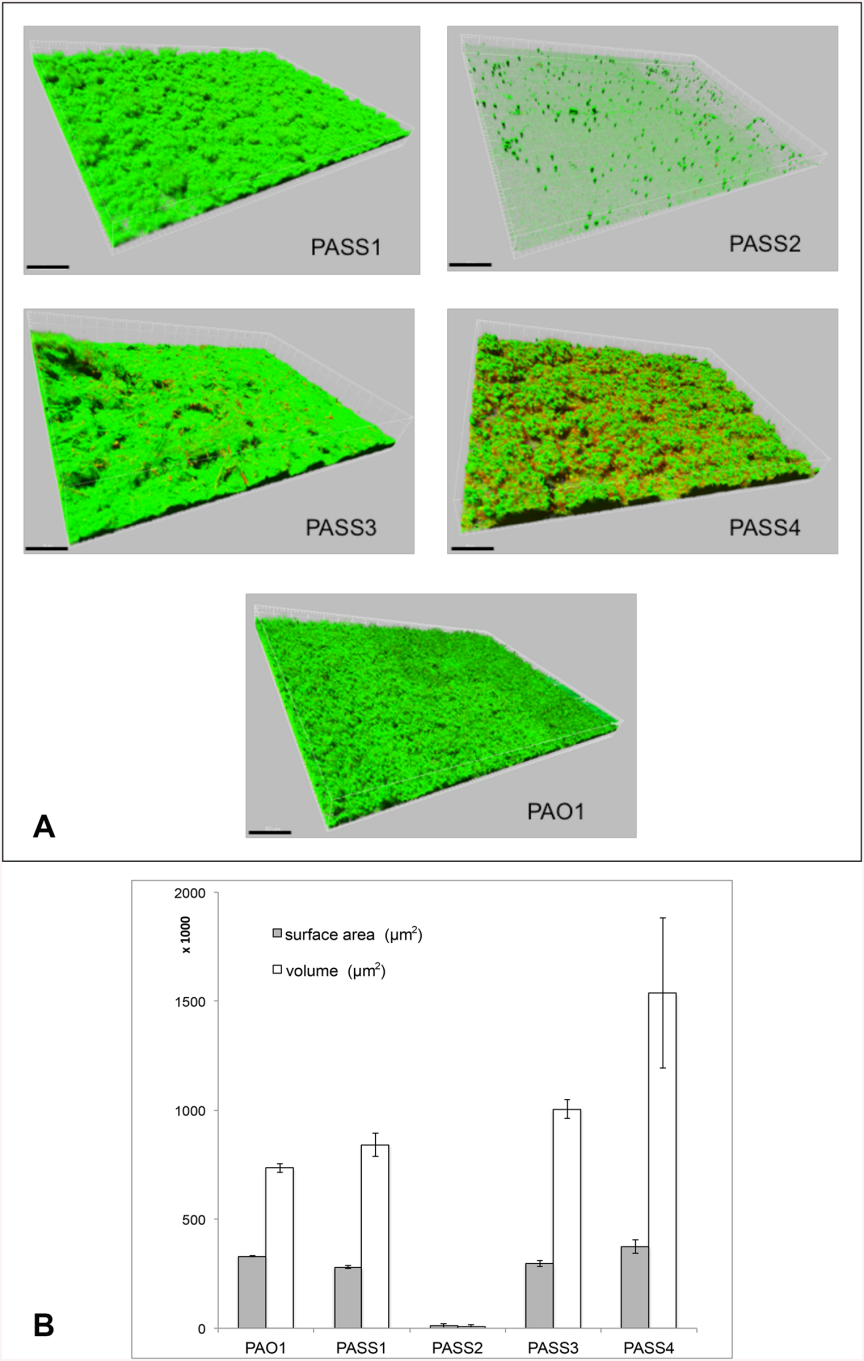
(A) Representative images of biofilms formed in flow cells by strains PASS1-4 and PAO1 after 48 hours of growth in LB medium at 37°C. (B) Quantitative analysis of quadruplicate LSCM images (derived from two independent flow cell chambers that were inoculated from separate overnight cultures, and two distant fields of view in each chamber) was performed using Imaris software. Images were taken using Olympus FV1000 confocal laser-scanning microscopy (LSCM) system, 3D pictures were built using Imaris software package (Bitplane). Cells are stained using BacLight Live/Dead stain (Molecular Probes); live cells are presented in green, dead cells—in red. Scale bars on LSCM images represent 50 μm.

Biofilm formation in flow chambers highlighted differences in the biofilm forming ability and biofilm architecture of the *P*. *aeruginosa* strains. PASS1, PASS3 and PASS4 were shown to form thicker biofilms compared to PAO1 (Fig 5A and 5B). Conversely, strain PASS2 was not able to form a biofilm on the surface of glass coverslips in the flow chambers. This is likely due to the loss of the *psl* cluster of genes responsible for the biosynthesis of exopolysacharide Psl located within the PA2218-PA2354 region absent in this strain (Fig 3). Psl is one of the major exopolysaccharides involved in the formation of biofilm matrix in *P*. *aeruginosa*, along with alginate and Pel. Non-mucoid strains, such as PASS2, primarily utilize either the Psl or Pel polysaccharides for biofilm formation [30, 31]. Previous mutational analyses demonstrated that Psl plays an important role in surface attachment for most isolates and the Psl deficiency often leads to the lack of biofilm formation [30].

Extracellular substances like exopolysaccharide Psl, are often regarded as “public goods” as they can benefit the overall population and not only the single producer strain [32]. Production of such metabolites is often metabolically expensive. This inevitably leads to the emergence of “social cheaters”–phenotypes that do not produce certain public goods, and, therefore, do not bear the cost of their production, but still benefit from metabolites produced by other members of the community [33, 34]. Hence, it is possible that strain PASS2 may have evolved as a “cheater” phenotype and may still be able to form biofilms in the mixed communities with other strains present in the CF lung, benefiting from Psl produced by other strains. PASS2 was the only *P*. *aeruginosa* strain isolated from that particular CF patient, so it is not possible to directly investigate this “cheater” hypothesis.

### Flagella and pili

Flagella and pili are important factors in biofilm formation, especially in the initial stages of attachment. Thus, flagella, besides being motility organelles, also play a direct role in virulence as major antigenic determinants for the immune response to *P*. *aeruginosa* infection [35]. PASS1, PASS3 and PAO1 shared a similar organization of flagellar biogenesis genes, while PASS2 and PASS4 showed distinct differences (S1A Fig). Based on the molecular weight and serological properties of flagellin, *P*. *aeruginosa* have been previously classified into two groups carrying A- and B- type flagellins [36, 37]. PASS1 and PASS3 share four genes orthologous to PA1088-PA1091 of PAO1, typical of B-type flagellins [38]. In contrast, the same region in PASS2 and PASS4 had a more complex polymorphic organisation characteristic of strains with a highly glycosylated and more heterogenous type A flagellin The duplication of the *fliS* gene observed in PASS2 and PASS4 was also characteristic for type A flagella [38].

It is largely accepted that glycosylation of flagellins is important for virulence and host specificity, however the precise physiological activity remains unclear [39, 40]. Strains PASS2 and PASS4 exhibited little or no flagellum-dependent swimming motility as opposed to PAO1, PASS1 and PASS3 (S1B Fig); whether this is a general feature of type A flagellin has yet to be elucidated.

**Fig 6:**
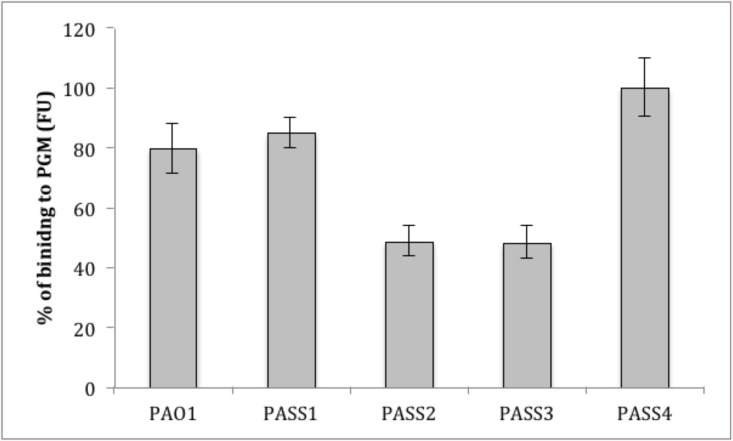
Binding of isolates PASS1-4 and PAO1 to porcine gastric mucine (PGM). The Y-axis represents the binding to mucin by the different bacterial strains as arbitrary fluorescent units (FU) normalised against the maximum bacterial binding of PASS4.

Flagellar components have been shown to be important for adhesion to mucins [41]. Mucins are major macromolecular glycoprotein components of the mucus that line the surface of lung epithelium and that vary in their composition and bacterial adhesion properties in CF [42, 43]. Mucins are overproduced in the lungs of CF patients and binding of *P*. *aeruginosa* to lung epithelial mucins in CF has been previously reported [44]. The mucin-binding assay showed variations amongst the isolates, with PASS4 showing the highest level of binding to mucin. Notably, PASS2 binding to mucin was comparable to that of PASS3 (Fig 6) despite the decreased biofilm formation in flow cells (Fig 5A and 5B). This data suggests that adhesion to biological surfaces, such as to the mucins on the epithelium, is multifactorial and bacteria may employ various tools for attachment to surfaces that may allow them to at least partially compensate for a loss of another mechanism.

Bacterial type IV pili (T4P) have been extensively studied for their contribution to motility, biofilm formation and virulence [45]. T4P may, therefore, play a major role in the colonization, survival and virulence in the CF lung. Based on genomic context of T4P genes, pili are classified into groups I-V [46]. PAO1 has been shown to encode a group II pilin [46], this is also true for strains PASS1 and PASS3 based on our analyses, whereas strains PASS2 and PASS4 were found to encode group I pili. Interestingly, Kus et al. [46] have described the high prevalence of group I pili in isolates from CF patients, possibly representing a specific adaptation to the CF lung environment. Nevertheless, the significance and specific role of group I T4P in CF is currently unclear.

### Phenazine production

Phenazines are secondary metabolites produced by a variety of bacteria, most notably pseudomonads, and have been studied intensively because of their broad-spectrum antibiotic properties and proven role in virulence. Many phenazine-producing bacteria are commonly found associated with host organisms [47]. The well-known phenazines produced by *P*. *aeruginosa* include phenazine-carboxylic acid (PCA), phenazine-carboxamide (PCN) and hydroxyl-phenazine (1-OH-PHZ), and especially pyocyanin (PYO). The latter is produced in concentrations close to 100 μmol/L during infection in CF [48, 49]; and its presence is associated with high morbidity and mortality in CF patients [50, 51].

**Fig 7:**
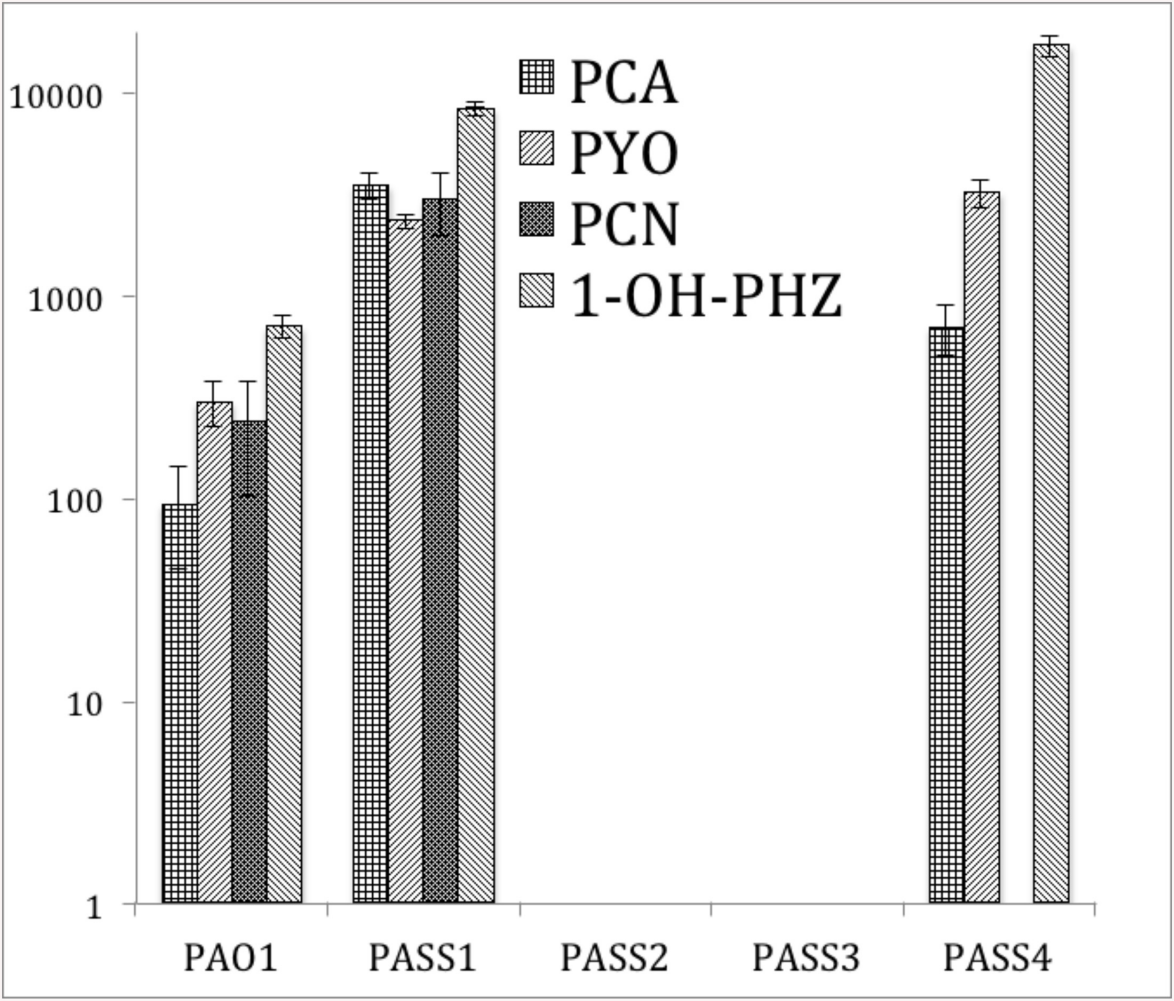
Production of the phenazines phenazine-carboxylic acid (PCA), pyocyanin (PYO), phenazine-carboxamide (PCN) and hydroxyl-phenazine (1-OH-PHZ) as assessed by UHPLC. The Y-axis represents arbitrary values based on chromatographic peak areas representative for each compound as observed at 257 nm, in logarithmic scale. Error bars represent standard deviations between three biological replicates.

**Fig 8:**
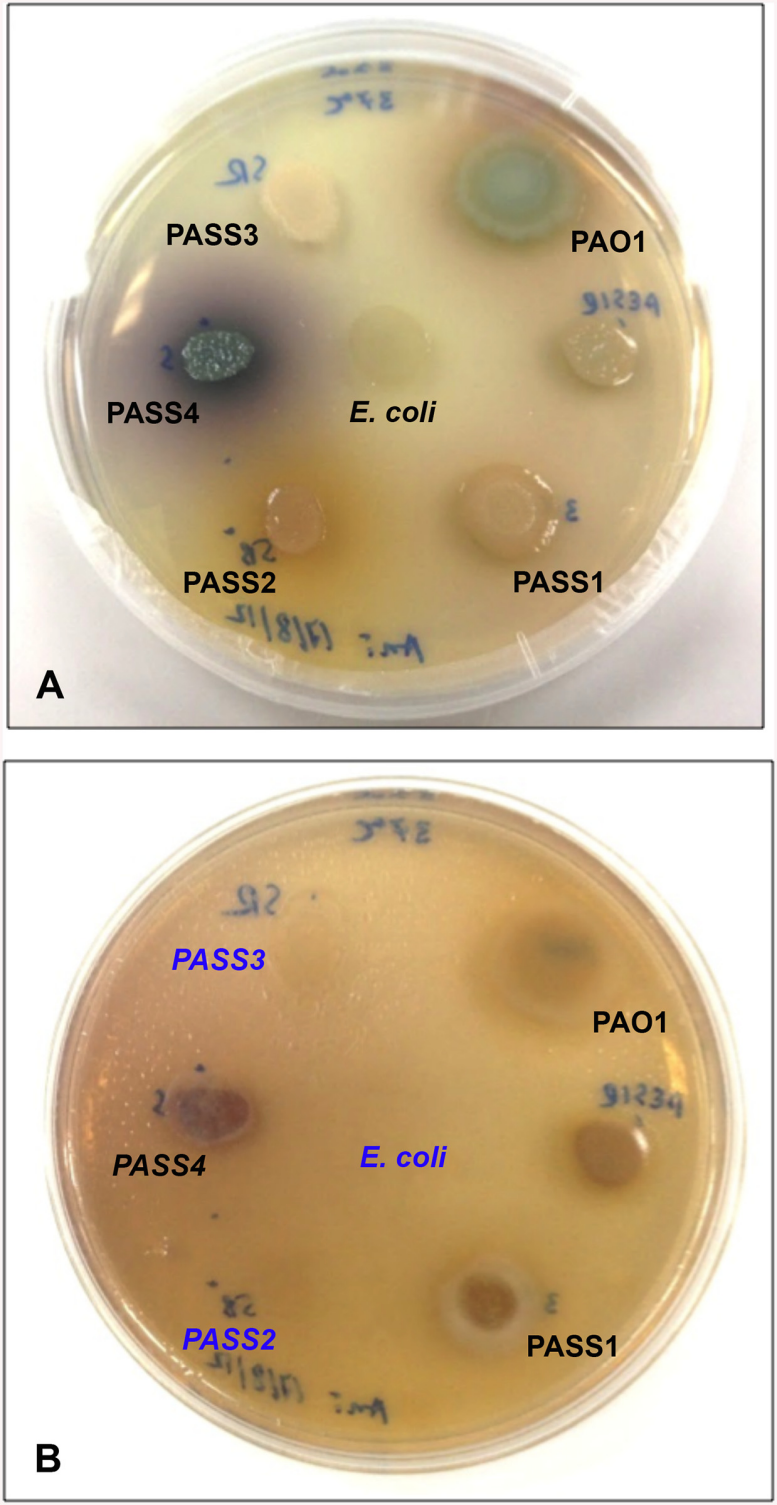
Nematode selective grazing assay using *Caenorhabditis elegans*. A representative Petri dish with colonies of various *P*. *aeruginosa* isolates before *C*. *elegans* inoculation (A) and after the 2-week incubation with *C*. *elegans* (B). Colonies of isolates that have been completely grazed by nematodes, and, thus represent the less-toxic/less-virulent phenotypes, are shown in blue. *Escherichia coli* OP50 was used as a positive control for *C*. *elegans* grazing.

Lau et al, using PYO-deficient mutants, have provided direct evidence that PYO is among the most potent virulence factors in the arsenal of *P*. *aeruginosa*. Mutants lacking PYO production were attenuated in their ability to infect mouse lungs in an acute pneumonia model of infection when compared with isogenic wild-type bacteria [52]. Ultra High Performance Liquid Chromatography (UHPLC) analyses revealed that PASS2 and PASS3 did not produce detectable phenazines (Fig 7). This was confirmed in a *Caenorhabditis elegans* selective grazing assay, where PASS2 and PASS3 were completely grazed by nematodes, indicative of a less-toxic/less-virulent phenotype, whereas all other *P*. *aeruginosa* colonies remained intact (Fig 8). The activity of phenazines as anti-nematode compounds against *C*. *elegans* has been recently demonstrated [53]. Production of the phenazine carboxamide was not observed in the strain PASS4. There is no evidence for absence or a significant disruption of genes involved in the production and modification of phenazines in any of the PASS1-4 genomes.

### Production of pyoverdine

**Fig 9:**
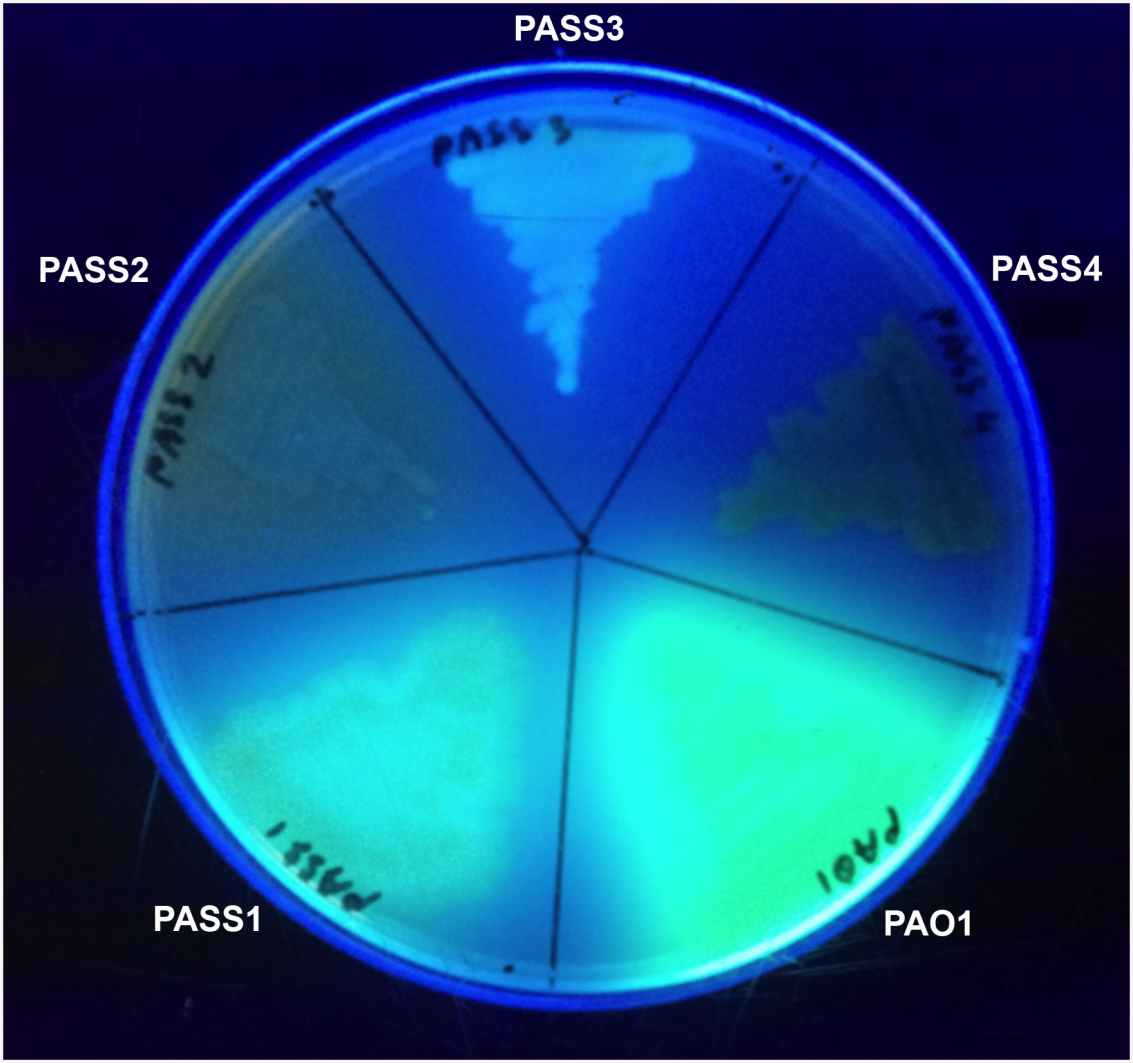
Representative image showing the production of fluorescent siderophore pyoverdine as assessed under the UV light.

One of the main characteristics of fluorescent pseudomonads is the production a fluorescent yellow-green siderophore, pyoverdine, important for iron acquisition in low iron environments [54]. PASS2 and PASS4 did not produce pyoverdine on iron-limited King’s medium, while PASS3 showed only trace amounts of pyoverdine as compared to PAO1 and PASS1 based on a pyoverdine production assay (Fig 9).

Genomic analysis revealed the loss of several genes in the pyoverdine biosynthesis pathway in both strains PASS2 and PASS4 including either complete or partial loss of genes orthologous to PA2398-PA2402 encoding the ferripyoverdine receptor FpvA, pyoverdine synthetase PvdD, protein PvdJ, and the peptide synthase, respectively; which would explain the lack of pyoverdine production in strains PASS 2 and PASS4. Longitudinal studies of CF patients have reported an increased occurrence of pyoverdine-negative isolates with chronic *P*. *aeruginosa* colonization [55, 56]. The increased availability of heme in CF lungs may select for the use of hemophores rather than siderophores by *P*. *aeruginosa* in the CF lung environment [56, 57].

The LasR quorum sensing (QS) regulator has a role in regulation of the production of virulence factors such as phenazines and pyoverdine, and *lasR* mutants have been frequently described among *P*. *aeruginosa* CF isolates [58, 59]. Sequence analysis of *lasR* in PASS1-4 strains revealed a single nucleotide polymorphism (SNP) in PASS1 and a 6-nucleotide insertion in PASS2 (Table 2). The same SNP observed in PASS1 was also present in the *lasR* gene in PASS3, as well as an additional deletion leading to a frameshift error. The *lasR* gene in PASS4 was identical to that of PAO1. From this analysis, PASS1-2 strains may be attenuated in the LasR regulator, while PASS3 would most certainly be LasR deficient. This may explain the decreased pyoverdine production by PASS3, despite it carrying an intact pyoverdine biosynthetic gene cluster. The inability of PASS2 and PASS4 to produce pyoverdine is almost certainly due to the loss of several pyoverdine biosynthetic genes. The lack of phenazine production by PASS2 and PASS3 might be due to these two strains having the most severe mutations in the *lasR* regulator.

**Table 2:** Summary of mutations in the *lasR* regulator gene correlated with the observed production of phenazines (PCA, PYO, PCN, 1-OH-PHZ) and pyoverdine (PVD), and the integrity of the pyoverdine biosynthesis gene cluster.

Strain	*lasR* variant	Predicted LasR effect	Production of phenazines	Production of PVD	PVD biosynthesis genomic deficiency (PA2398-PA2402)
PASS1	c.61G>A	p.(A21T)	positive	positive	non-deficient
PASS2	c.693_694insATGGCC	p.M231_A232insMA	negative	negative	deficient
PASS3	c.[61G>A; 124del]	p.[(A21T; D43fs*114)]	negative	decreased	non-deficient
PASS4	No changes	No changes	positive (no PCN)	negative	deficient

The deficiency in *lasR*, in conjunction with the deficiency in the *psl* cluster of genes responsible for the production of a major exopolysaccharide, may aid to the lifestyle of PASS2 as a potential “social cheater”. LasR deficiency may further render PASS2 “deaf” to QS signals and, therefore, limit its response in production of various metabolically costly QS-controlled virulence factors and toxins benefiting the bacterial population [60, 61].

### Other virulence factors

Several other virulence factor genes were missing in PASS2 and PASS4, compared to the genome of PAO1. The colicin-like pyocin S5 and its cognate immunity protein (encoded by PA0985 and PA0984 in PAO1 genome respectively), pyocin S4 (PA3866), a probable non-ribosomal peptide synthetase (PA2402), and phospholipase D (PA3487) were absent in both PASS2 and PASS4. In addition, PASS2 also lacks genes for the production of paerucumarin (PA2254-PA2257), chitinase (PA2300), and AMB toxin (PA2302-PA2306).

### Distinct *P*. *aeruginosa* lifestyle strategies in the CF lung

Based on genomic and phenotypic comparisons the four CF isolates obtained directly from the sputum of CF patients revealed significant differences in lifestyle strategy. PASS2 and PASS4 shared some common features with the loss of various virulence determinants, and similar organization of their pili and flagellar loci. PASS2 showed significant genome reduction, with the loss of additional virulence factors, narrowing of potential carbon substrates that can be utilized and even significant reduction in biofilm forming capacity due to the lack of polysaccharide Psl production. The defect in the LasR QS regulator in PASS2 probably also impacts its virulence potential. These adaptations in PASS2 may limit the host immune response as well as reducing expenditure of cellular resources, possibly representing a passive, “cheater” strategy [28].

In contrast, based on the increased production of phenazines such as pyocyanin and increased biofilm forming capacity, isolates PASS1 and PASS4 (the latter even despite the loss of several other virulence genes) may have taken a different adaptation path by enhancing their virulence and colonization potential, possibly representing more aggressive phenotypes. The virulence of PASS1 and PASS4 was also demonstrated against the eukaryotic model *C*. *elegans* (Fig 8). The virulent phenotype of PASS4 in the *C*. *elegans* model, despite the clear loss of known virulence factors, highlights an issue in making virulence predictions based on genomic analyses alone.

PASS3 is closely related to PASS1 based on MLST and genomic comparisons, but differs significantly in phenotype. PASS3 displays low toxicity in the *C*. *elegans* model and is deficient in phenazine production, possibly as a result of mutations in *lasR*. However, PASS3 shows enhanced biofilm formation and conversion to a mucoid phenotype, which likely enhances its survival and persistence, and therefore may have adopted a more “defensive” strategy.

Table 3 summarizes the overall genetic and phenotypic differences observed between PASS1-4 strains and PAO1. The diversity seen amongst the CF isolates in this and previous studies [62, 63] suggests that there is no single preferred adaptation pathway for success in a CF lung.

**Table 3:** Summary of genomic and phenotypic characteristics of *P*. *aeruginosa* CF isolates PASS1-4 and the model strain PAO1.

	PASS1	PASS2	PASS3	PASS4	PAO1
**Colony morphology on LB solid medium**	pale-green non-mucoid	light brown non-mucoid	white mucoid	green-blue non-mucoid	green non-mucoid
**Biofilm formation in flow cells**	positive	negative	positive	positive	positive
**Flagella**	type B	type A	type B	type A	type B
**Type IV pili**	group II	group I	group II	group I	group II
**Swimming motility**	positive	deficient	positive	deficient	positive
**Binding to mucin**	high (80–100%)	low (≤50%)	low (≤50%)	high (80–100%)	medium (50–80%)
**Phenazines production**	positive	negative	negative	positive (no PCN)	positive
**Pyoverdine production**	positive	negative	decreased	negative	positive
**LasR deficiency**	uncertain	possibly deficient	possibly deficient	non-deficient	non-deficient
**Loss of additional virulence factors**		pyocin S5, pyocin S4, NRPS, phospholipase D, paerucumarin, chitinase, AMD toxin		pyocin S5, pyocin S4, NRPS, phospholipase D,	
**Toxicity/ virulence** *	positive	negative	negative	positive	positive

* as assessed in *C*. *elegans* selective grazing assay

In order to enable researchers to broaden *P*. *aeruginosa* research beyond the limitations of model laboratory strains, recently a *P*. *aeruginosa* reference panel of 43 strains has been suggested, also based on the origin, in order to reflect its diversity [64]. Nevertheless, the diversity seen among our 4 isolates with each of these strains equipped with a unique set of defensive and offensive tools, suggests that differences that exist between strains that originate even from the same environment like CF can make it impossible to pinpoint a single phenotype/strain with a set of defined characteristics that would be representative for that environment.

### CF isolates share a core proteomic signature distinct from PAO1

A shotgun proteomic analysis was undertaken to investigate protein expression profiles of the four clinical isolates and the model *P*. *aeruginosa* strain. There were striking differences in protein expression between the common laboratory model organism PAO1 and the CF isolates PASS1-4. With ~ 1300–1400 proteins identified for each of these organisms, only 526 were shared among all five, while PAO1 showed 827 unique proteins not detected in any of the CF isolates when cultured in a common laboratory medium LB. Conversely, PASS1-4 strains shared 703 commonly expressed proteins, none of which were detected in PAO1 (Fig 4F, S2 Table). This trend can also be seen on FriPan 2D plot where PAO1 appears at a considerable distance from PASS1-4 strains, while CF strains PASS1-4 among themselves retained the overall similarity profiles with PASS1 and PASS3, and PASS2 and PASS4 clustering together (Fig 4D and 4E), consistent with the overall pattern observed in bioinformatics analysis for all predicted proteins (Fig 4A and 4B).

**Fig 10:**
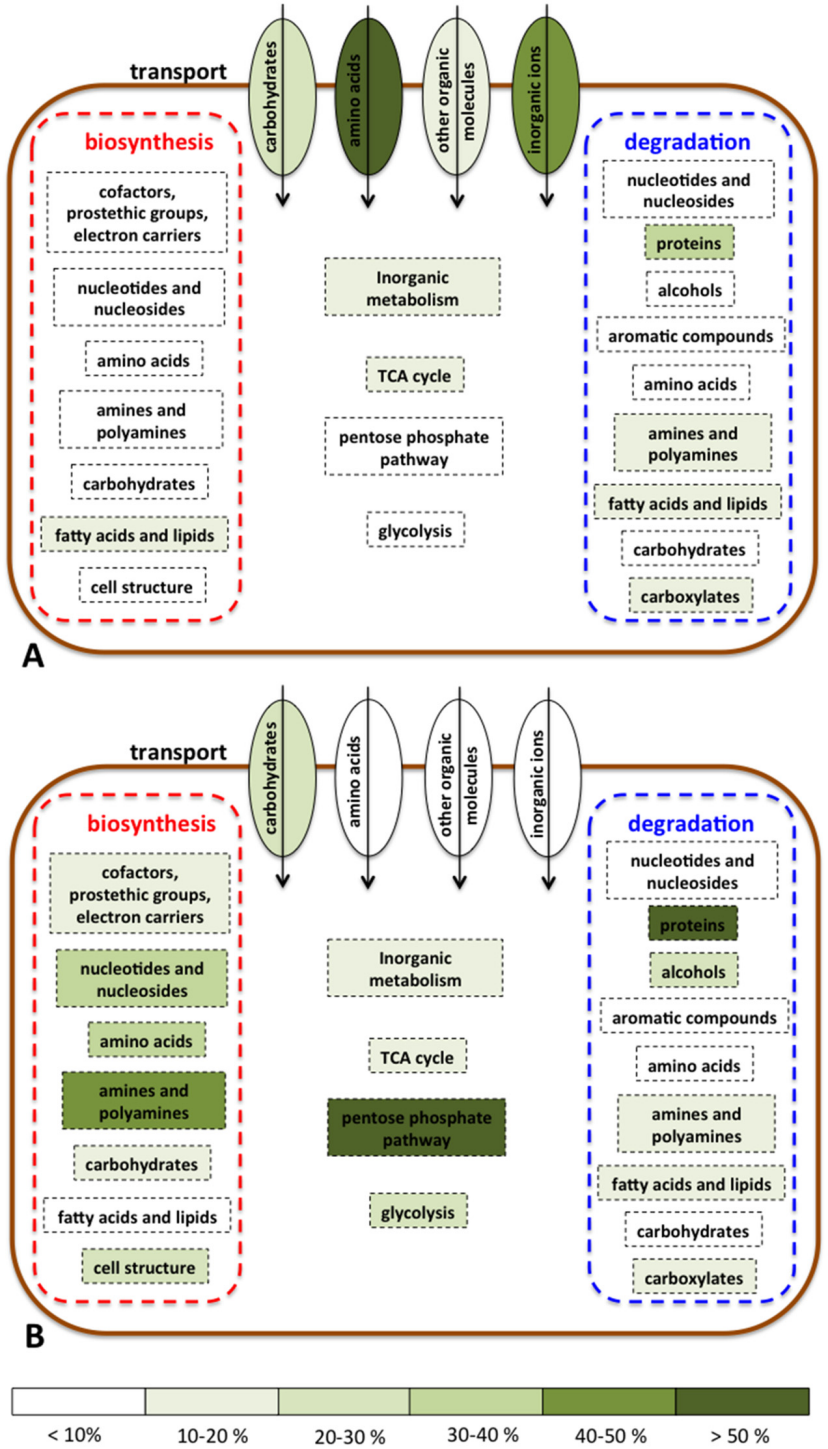
Schematic metabolic overview showing differences in protein abundance between PAO1 (A) and the CF isolates PASS1-4 (B) as assessed via proteomics. Shading represents the percentage of expressed proteins within each metabolic category. Assignment to metabolic categories is based on analysis using Pathway Tools [27]. Full details of the Pathway Tools analysis are shown in S2 and S3 Figs, and the full list of express proteins for each strain is provided in S2 Table.

The proteins uniquely expressed by PAO1 (827 in total) and those uniquely shared by PASS1-4 strains (703 in total), were mapped onto the metabolic pathways of PAO1 using Pathway Tools 17.5 (Fig 10, S2 and S3 Figs). The analysis revealed distinct differences in cell physiology between the CF strains and the model strain PAO1. PAO1 expressed an array of membrane transport proteins involved in the uptake of a broad range of amino acids, polyamines, carbohydrates and other organic nutrients, whose expression was not detected in any of the PASS1-4 strains. There was essentially no expression of biosynthetic genes involved in amino acid, polyamine, carbohydrates, nucleoside or nucleotide biosynthesis (Fig 10A, S2 Fig).

In contrast, PASS1-4 expressed only a small select group of transporters for compounds such as sugars, dipeptides, and heme. Instead the PASS1-4 strains expressed many proteins involved in biosynthetic pathways for compounds such as nucleosides and nucleotides, amino acids, carbohydrates and polyamines (Fig 10B, S3 Fig).

The proteomic data indicates that in a conventional laboratory medium PAO1 may transport a diverse set of “ready-made” nutrients from the rich LB medium, whereas CF isolates PASS1-4 require only a limited number of nutrients from the LB medium, relying mainly on their own metabolism for synthesis of other essential nutrients. Interestingly, despite dramatic differences in the proteins involved in biosynthesis, in general, the expression of proteins involved in the catabolism of various compound groups were similar. This might indicate that PAO1 and PASS1-4 strains all have access to a variety of compound classes, but, as mentioned above, may vary significantly in the means of obtaining these compounds: PAO1 –via transport, and PASS1-4 –via intracellular biosynthesis. This probably reflects specialization in the CF strains geared towards utilization of select types of nutrients that might be abundant in CF lungs, such as highly glycosylated mucins, and further highlights the preference for heme as a possible source of iron in CF isolates. Conversely, PAO1, having been grown and maintained in laboratories worldwide for decades, may have adapted to using the broad variety of nutrients present in common growth media, such as LB. The proteomics data reveals significant differences in the metabolic strategies of CF isolates compared with PAO1 and emphasises the limitations presented by the use of laboratory model organisms for studying processes in specific hosts or environments.

### Concluding remarks

Significant phenotypic differences were observed amongst the *P*. *aeruginosa* CF isolates PASS1-4 including differences in traits important for successful CF lung colonization and survival. Phenotypic diversity has been previously shown among the *P*. *aeruginosa* CF isolates [62, 63], particularly with respect to the decreased virulence in chronic infections [65–67]. While the classical reduction of virulence seen in many CF isolates was observed in PASS2, and the conversion to mucoid phenotype by PASS3, a more aggressive phenotype was shown by PASS1 and PASS4 with increased production of known virulence factors such as phenazines, and increased biofilm formation. This suggests that there is no single pathway of adaptation to an environment such as the CF lung; instead, strains acquire adaptations that allow them to pursue different lifestyles ranging from passive, defensive to aggressive. This makes it extremely difficult to predict the development of certain traits in a particular environment or host, and, hence, make assumptions for the suitable treatment strategies.

Relying on genomic data to make predictions about phenotypes can be problematic. *In silico* analysis of genomes of all 5 isolates in this study revealed a high degree of similarity between the strains with 80% of shared predicted proteome, which is in the range commonly observed for strains in the same species implying possible phenotypic similarities. However, actual protein expression by these strains grown in a common laboratory medium revealed dramatically different expression profiles suggesting distinct physiological and metabolic states that were not predictable at the genomic level via *in silico* analysis. This further highlights the limitations of model laboratory strains and the need to complement analyses in model organisms with direct experimental work on isolates from the relevant host or environment.

## Supporting Information

S1 Fig Genomic alignment of flagella biogenesis genes in strains PASS1-4 as compared to PAO1 using MAUVE. Sequences conserved among all 5 isolates are presented in mauve, sequences shared between isolates PAO1, PASS1 and PASS3 are in green, sequences shared between isolates PASS2 and PASS4 are presented in blue **(A)**. **Flagella-mediated swimming motility assay for strains PASS1-4 and PAO1 (B).**
(TIF)

S2 Fig Pathway Tools 17.5 cellular overview diagram showing proteins/pathways expressed uniquely by PAO1, in red. The shapes on the diagram represent specific types of compounds as follows: square—carbohydrate, triangle—amino acid, upside down triangle—cofactor, ellipse (horizontal)—purine, ellipse (vertical)—pyrimidine, diamond—proteins, T-shape—tRNA, circle—all other compounds. The shading of the icon indicates the phosphorylation state of the compound: shaded compounds are phosphorylated; unshaded compounds are unphosphorylated. Shapes/paths located on outer rectangles represent transporters/membrane proteins. The arrows indicate the direction of the transport: pointing in—uptake, pointing out—export.
(TIF)

S3 Fig Pathway Tools 17.5 cellular overview diagram showing proteins/pathways expressed uniquely by PASS1-4 strains, in red. The shapes on the diagram represent specific types of compounds as follows: square—carbohydrate, triangle—amino acid, upside down triangle—cofactor, ellipse (horizontal)—purine, ellipse (vertical)—pyrimidine, diamond—proteins, T-shape—tRNA, circle—all other compounds. The shading of the icon indicates the phosphorylation state of the compound: shaded compounds are phosphorylated; unshaded compounds are unphosphorylated. Shapes/paths located on outer rectangles represent transporters/membrane proteins. The arrows indicate the direction of the transport: pointing in—uptake, pointing out—export.
(TIF)

S1 Table MLST allelic profiles and strain types of *P*. *aeruginosa* isolates obtained from the CF sputum in this study. (DOCX)

S2 Table Proteins identified in strains PASS1-4 and PAO1 grown in LB medium via proteomics (p < 0.01). + protein identified in the strain culture (shaded),—protein not identified in the strain culture (unshaded), NA—protein does not have an ortholog in the PAO1 genome or did not map to an ortholog in PAO1 due to differences in the sequence.
(DOCX)
