# Supplementary figures and images for: Genetically and Phenotypically Distinct Pseudomonas aeruginosa Cystic Fibrosis Isolates Share a Core Proteomic Signature
Source: PLoS One. 2015 Oct 2;10(10):e0138527. doi: 10.1371/journal.pone.0138527 (PMC4592193; doi:10.1371/journal.pone.0138527)

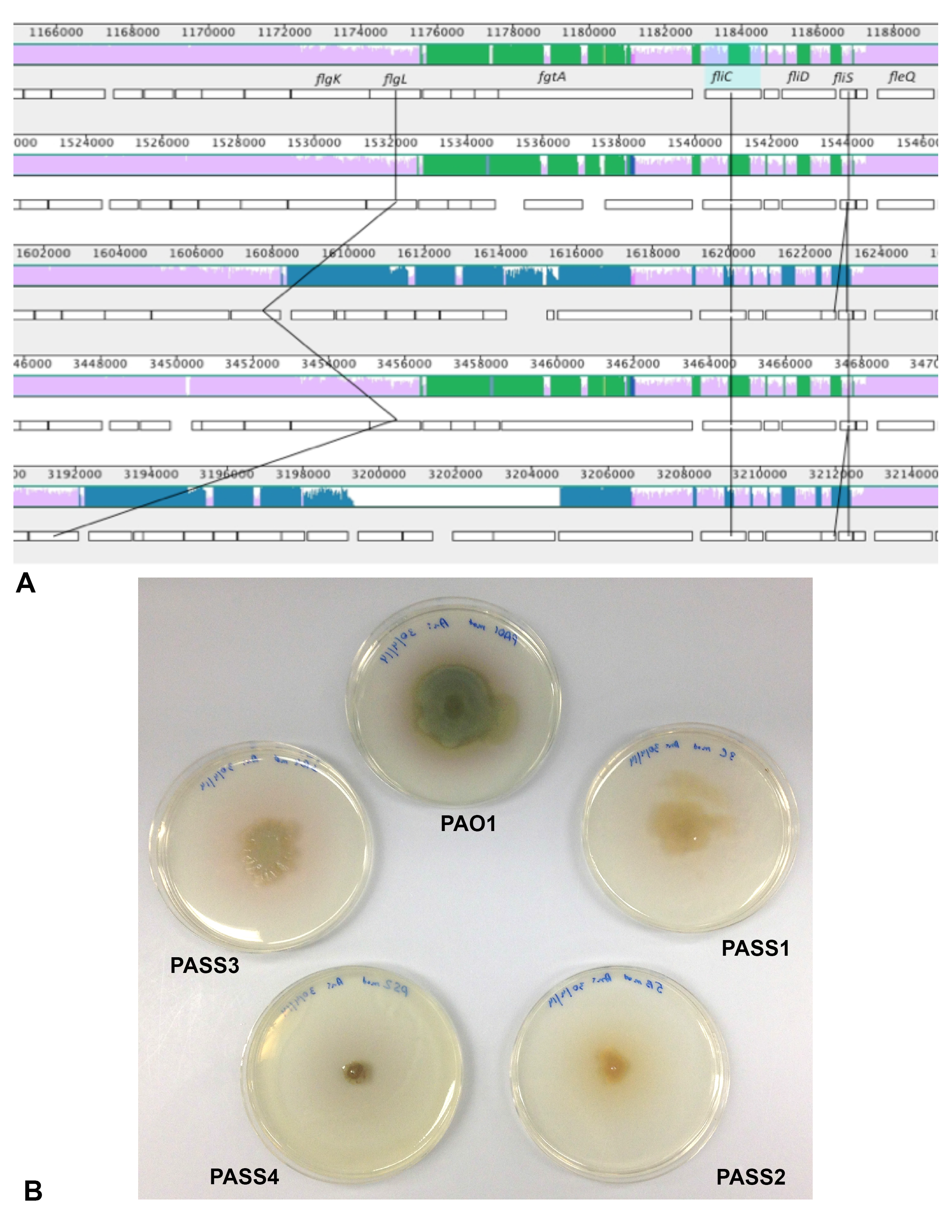

Supplement: S1 Fig — Sequences conserved among all 5 isolates are presented in mauve, sequences shared between isolates PAO1, PASS1 and PASS3 are in green, sequences shared between isolates PASS2 and PASS4 are presented in blue (A). Flagella-mediated swimming motility assay for strains PASS1-4 and PAO1 (B). (TIF) [file pone.0138527.s001.tif]

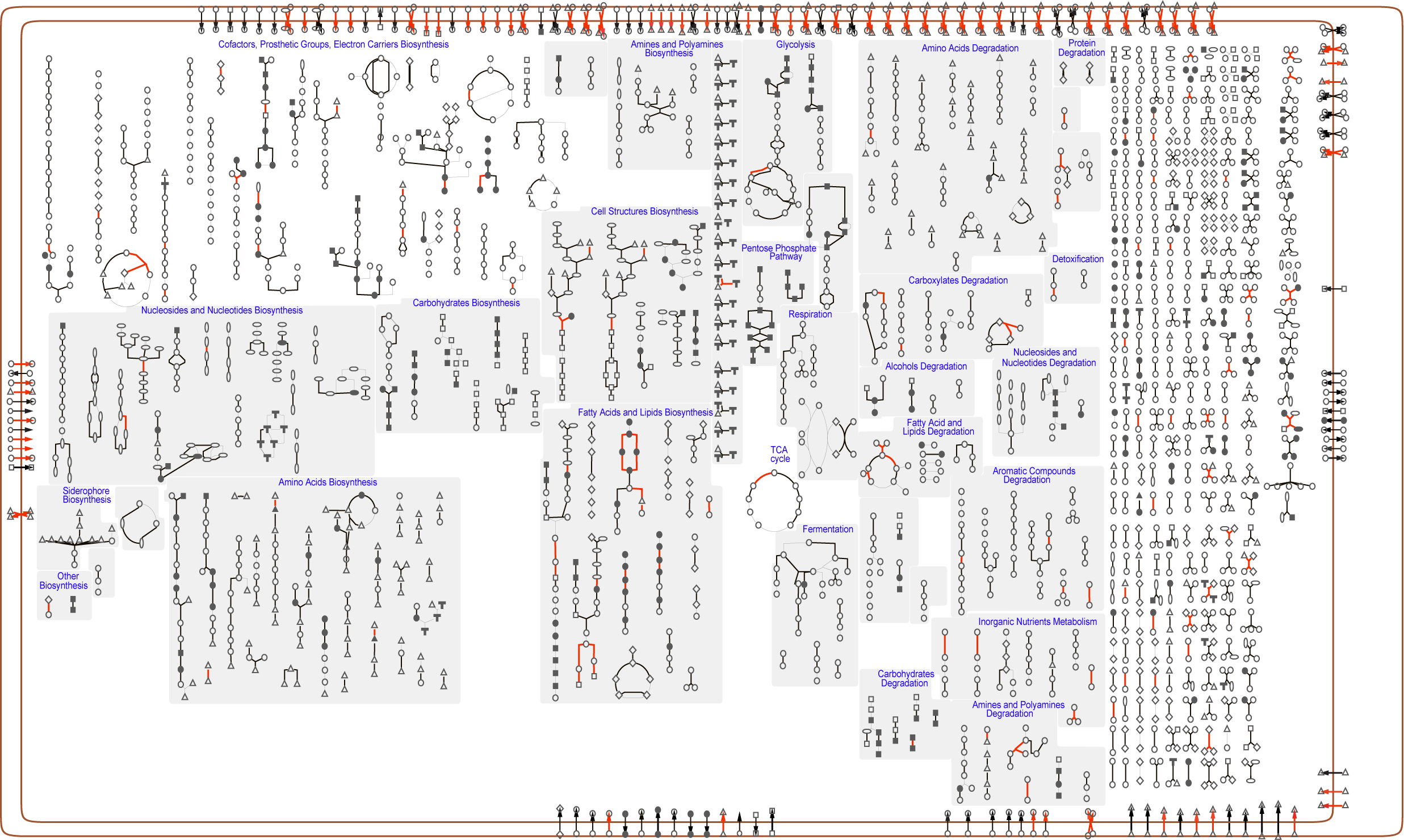

Supplement: S2 Fig — The shapes on the diagram represent specific types of compounds as follows: square—carbohydrate, triangle—amino acid, upside down triangle—cofactor, ellipse (horizontal)—purine, ellipse (vertical)—pyrimidine, diamond—proteins, T-shape—tRNA, circle—all other compounds. The shading of the icon indicates the phosphorylation state of the compound: shaded compounds are phosphorylated; unshaded compounds are unphosphorylated. Shapes/paths located on outer rectangles represent transporters/membrane proteins. The arrows indicate the direction of the transport: pointing in—uptake, pointing out—export. (TIF) [file pone.0138527.s002.tif]

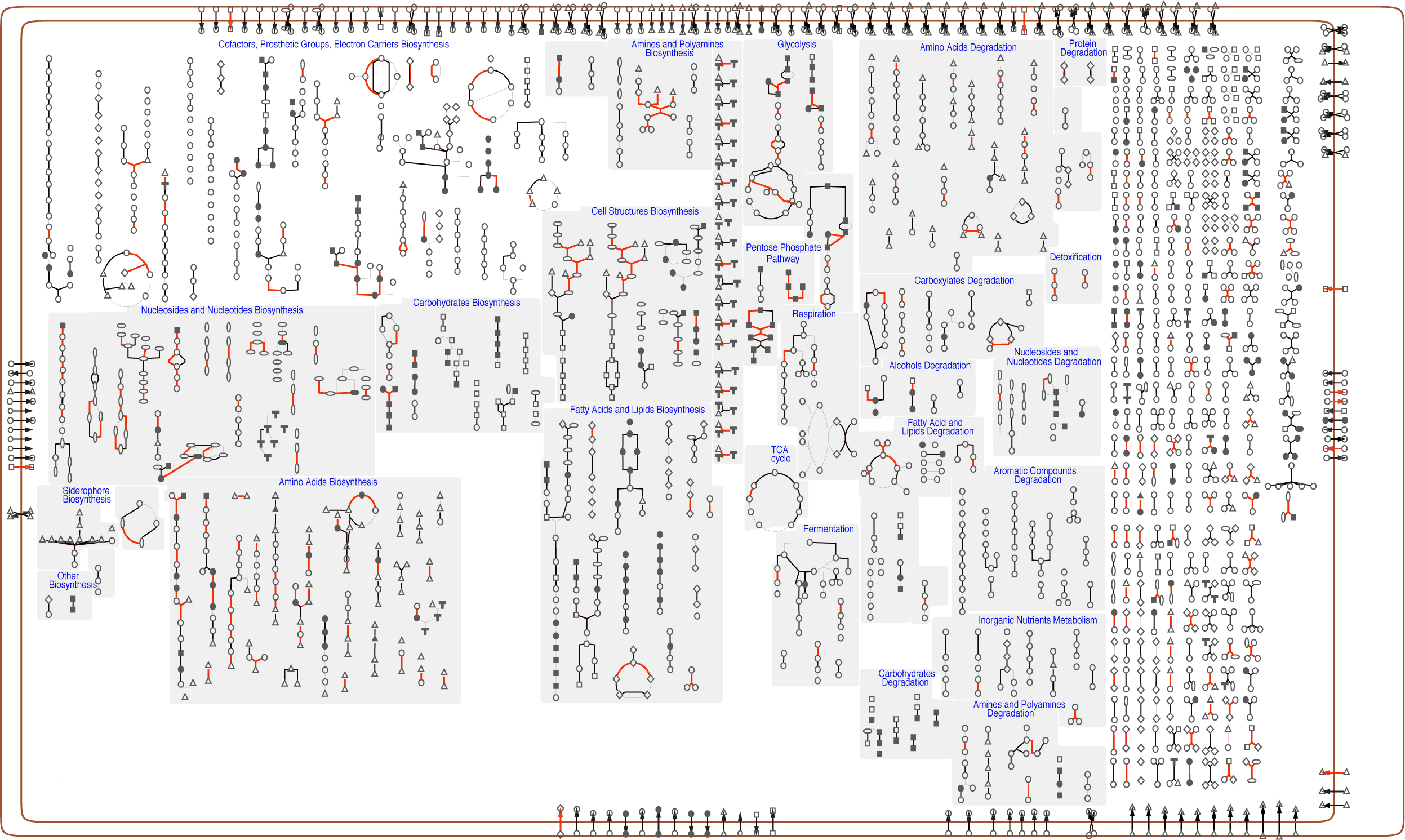

Supplement: S3 Fig — The shapes on the diagram represent specific types of compounds as follows: square—carbohydrate, triangle—amino acid, upside down triangle—cofactor, ellipse (horizontal)—purine, ellipse (vertical)—pyrimidine, diamond—proteins, T-shape—tRNA, circle—all other compounds. The shading of the icon indicates the phosphorylation state of the compound: shaded compounds are phosphorylated; unshaded compounds are unphosphorylated. Shapes/paths located on outer rectangles represent transporters/membrane proteins. The arrows indicate the direction of the transport: pointing in—uptake, pointing out—export. (TIF) [file pone.0138527.s003.tif]
