# Supplementary material for: Genetically and Phenotypically Distinct Pseudomonas aeruginosa Cystic Fibrosis Isolates Share a Core Proteomic Signature
Source: PLoS One. 2015 Oct 2;10(10):e0138527. doi: 10.1371/journal.pone.0138527 (PMC4592193; doi:10.1371/journal.pone.0138527)
Supplement: S1 Table — (DOCX) [file pone.0138527.s004.docx]

**S1 Table. MLST allelic profiles and strain types (ST) of *P. aeruginosa* isolates obtained from the CF sputum in this study.**

|  | acs | aro | gua | mut | nuo | pps | trp | ST |
| --- | --- | --- | --- | --- | --- | --- | --- | --- |
| PASS1 | 17 | 5 | 11 | 72 | 3 | 4 | 3 | new |
| PASS2 | 16 | 5 | 30 | 72 | 4 | 13 |  | new |
| PASS3 | 17 | 5 | 11 | 72 | 3 | 4 | 3 | new |
| PASS4 | 11 | 84 | 11 | 3 | 4 | 4 | 7 | 649 |
