# Supplementary material for: Genetically and Phenotypically Distinct Pseudomonas aeruginosa Cystic Fibrosis Isolates Share a Core Proteomic Signature
Source: PLoS One. 2015 Oct 2;10(10):e0138527. doi: 10.1371/journal.pone.0138527 (PMC4592193; doi:10.1371/journal.pone.0138527)
Supplement: S2 Table — + protein identified in the strain culture (shaded),—protein not identified in the strain culture (unshaded), NA—protein does not have an ortholog in the PAO1 genome or did not map to an ortholog in PAO1 due to differences in the sequence. (DOCX) [file pone.0138527.s005.docx]

**S2 Table. Proteins identified in strains PASS1-4 and PAO1 grown in LB medium via proteomics (p < 0.01).**

+ protein identified in the strain culture (shaded),

- protein not identified in the strain culture (unshaded)

NA - protein does not have an ortholog in the PAO1 genome or did not map to an ortholog in PAO1 due to differences in the sequence

| PAO1 LOCUS TAG | DESCRIPTION | PASS1 | PASS2 | PASS3 | PASS4 | PAO1 |
| --- | --- | --- | --- | --- | --- | --- |
| PA0001 | chromosomal replication initiator protein DnaA | + | + | + | + | + |
| PA0002 | DNA polymerase III, beta chain | + | + | + | + | - |
| PA0003 | RecF protein | - | - | - | - | + |
| PA0004 | DNA gyrase subunit B | + | + | + | + | - |
| PA0005 | lysophosphatidic acid acyltransferase, LptA | - | - | - | - | + |
| PA0006 | conserved hypothetical protein | + | + | + | + | + |
| PA0007 | hypothetical protein | + | + | + | + | + |
| PA0008 | glycyl-tRNA synthetase beta chain | + | + | + | + | + |
| PA0009 | glycyl-tRNA synthetase alpha chain | + | + | + | + | - |
| PA0017 | conserved hypothetical protein | - | - | - | + | + |
| PA0018 | methionyl-tRNA formyltransferase | + | + | + | - | + |
| PA0019 | polypeptide deformylase | + | + | + | + | + |
| PA0020 | T4P secretin-associated protein TsaP | + | + | + | + | - |
| PA0022 | conserved hypothetical protein | - | - | - | - | + |
| PA0023 | quinone oxidoreductase | + | + | + | + | + |
| PA0024 | coproporphyrinogen III oxidase, aerobic | + | + | + | + | + |
| PA0025 | shikimate dehydrogenase | + | + | + | + | - |
| PA0035 | tryptophan synthase alpha chain | + | - | - | - | + |
| PA0036 | tryptophan synthase beta chain | + | + | + | + | - |
| PA0037 | transcriptional regulator TrpI | - | - | - | - | + |
| PA0038 | hypothetical protein | + | + | + | + | + |
| PA0039 | hypothetical protein | + | + | + | + | - |
| PA0041 | probable hemagglutinin | - | + | - | - | - |
| PA0056 | probable transcriptional regulator | - | - | - | - | + |
| PA0057 | hypothetical protein | + | + | + | + | - |
| PA0058 | DsbM | - | - | - | - | + |
| PA0059 | osmotically inducible protein OsmC | + | + | + | + | - |
| PA0064 | hypothetical protein | - | - | - | - | + |
| PA0065 | hypothetical protein | + | + | + | + | + |
| PA0066 | conserved hypothetical protein | + | + | + | + | + |
| PA0067 | oligopeptidase A | + | + | + | + | - |
| PA0069 | conserved hypothetical protein | - | - | - | - | + |
| PA0070 | TagQ1 | + | + | + | + | - |
| PA0074 | serine/threonine protein kinase PpkA | - | - | - | - | + |
| PA0075 | PppA | + | + | + | + | - |
| PA0082 | TssA1 | - | - | - | - | + |
| PA0083 | TssB1 | + | + | + | + | + |
| PA0084 | TssC1 | + | + | + | + | + |
| PA0085 | Hcp1 | + | + | + | + | + |
| PA0086 | TagJ1 | + | + | + | + | - |
| PA0089 | TssG1 | - | - | - | - | + |
| PA0090 | ClpV1 | + | + | + | + | - |
| PA0093 | Tse6 | - | - | - | - | + |
| PA0094 | hypothetical protein | - | - | + | - | - |
| PA0099 | hypothetical protein | - | - | - | - | + |
| PA0101 | hypothetical protein | - | - | - | - | + |
| PA0102 | probable carbonic anhydrase | + | + | + | + | - |
| PA0114 | SenC | - | - | - | - | + |
| PA0115 | conserved hypothetical protein | + | + | + | + | - |
| PA0117 | probable short chain dehydrogenase | - | - | - | - | + |
| PA0118 | hypothetical protein | + | + | + | + | - |
| PA0121 | hypothetical protein | - | - | + | - | + |
| PA0122 | rahU | + | + | + | + | - |
| PA0127 | hypothetical protein | - | - | - | - | + |
| PA0128 | conserved hypothetical protein | + | + | + | + | - |
| PA0129 | Amino acid permease | - | - | - | - | + |
| PA0130 | 3-Oxopropanoate dehydrogenase | + | + | + | + | - |
| PA0131 | BauB | - | - | - | - | + |
| PA0138 | probable permease of ABC transporter | - | - | - | - | + |
| PA0139 | alkyl hydroperoxide reductase subunit C | + | + | + | + | + |
| PA0140 | alkyl hydroperoxide reductase subunit F | + | + | + | + | + |
| PA0141 | conserved hypothetical protein | + | + | + | + | - |
| PA0142 | hypothetical protein | - | - | - | - | + |
| PA0143 | purine nucleosidase Nuh | + | + | + | + | - |
| PA0147 | probable oxidoreductase | - | - | - | - | + |
| PA0148 | adenine deaminase | + | + | + | + | - |
| PA0161 | hypothetical protein | - | - | - | - | + |
| PA0162 | histidine porin OpdC | + | + | + | + | - |
| PA0164 | probable gamma-glutamyltranspeptidase | - | - | - | - | + |
| PA0169 | SiaD | - | - | - | - | + |
| PA0170 | hypothetical protein | + | + | + | + | - |
| PA0175 | probable chemotaxis protein methyltransferase | - | - | - | - | + |
| PA0176 | aerotaxis transducer Aer2 | + | + | + | + | - |
| PA0195 | putative NAD(P) transhydrogenase, subunit alpha part 1 | + | - | - | - | - |
| PA0224 | probable aldolase | - | + | - | - | - |
| PA0225 | probable transcriptional regulator | + | - | - | - | - |
| PA0230 | 3-carboxy-cis,cis-muconate cycloisomerase | - | - | - | - | + |
| PA0231 | beta-ketoadipate enol-lactone hydrolase | + | - | - | - | - |
| PA0249 | probable acetyltransferase | - | - | - | - | + |
| PA0250 | conserved hypothetical protein | + | + | + | + | - |
| PA0260 | Tle3 | - | - | - | - | + |
| PA0264 | hypothetical protein | - | - | - | - | + |
| PA0265 | succinate-semialdehyde dehydrogenase | + | + | + | + | + |
| PA0266 | 4-aminobutyrate aminotransferase | + | + | + | + | - |
| PA0268 | probable transcriptional regulator | - | - | - | - | + |
| PA0269 | conserved hypothetical protein | + | + | + | + | + |
| PA0270 | hypothetical protein | - | + | - | + | - |
| PA0276 | hypothetical protein | - | - | - | - | + |
| PA0277 | conserved hypothetical protein | + | + | + | + | - |
| PA0282 | sulfate transport protein CysT | - | - | - | - | + |
| PA0283 | sulfate-binding protein precursor | + | + | + | + | + |
| PA0284 | hypothetical protein | + | + | + | + | - |
| PA0290 | hypothetical protein | - | - | - | - | + |
| PA0291 | Anaerobically-induced outer membrane porin OprE precursor | + | + | + | + | + |
| PA0292 | agmatine deiminase | + | + | + | + | + |
| PA0293 | N-carbamoylputrescine amidohydrolase | + | + | + | + | - |
| PA0294 | transcriptional regulator AguR | - | - | - | - | + |
| PA0295 | probable periplasmic polyamine binding protein | + | + | + | + | + |
| PA0297 | probable glutamine amidotransferase | + | + | + | + | + |
| PA0298 | Glutamylpolyamine synthetase | + | + | + | + | + |
| PA0299 | Polyamine:pyruvate transaminase | + | + | + | + | + |
| PA0300 | polyamine transport protein | + | + | + | + | + |
| PA0301 | polyamine transport protein | + | + | + | + | - |
| PA0308 | hypothetical protein | - | - | - | - | + |
| PA0309 | hypothetical protein | + | + | + | + | - |
| PA0313 | L-cysteine transporter of ABC system YecS | - | - | - | - | + |
| PA0314 | L-cysteine transporter of ABC system FliY | + | + | + | + | + |
| PA0315 | hypothetical protein | + | + | + | + | + |
| PA0316 | D-3-phosphoglycerate dehydrogenase | + | + | + | + | + |
| PA0317 | hypothetical protein | + | + | + | + | + |
| PA0318 | conserved hypothetical protein | + | + | + | + | - |
| PA0322 | probable transporter | - | - | - | - | + |
| PA0323 | probable binding protein component of ABC transporter | + | + | + | + | - |
| PA0328 | arginine-specific autotransporter of Pseudomonas aeruginosa, AaaA | - | - | - | - | + |
| PA0329 | conserved hypothetical protein | + | + | + | + | + |
| PA0330 | ribose 5-phosphate isomerase | + | + | + | + | + |
| PA0331 | threonine dehydratase, biosynthetic | + | + | + | + | - |
| PA0334 | probable major facilitator superfamily (MFS) transporter | - | - | - | - | + |
| PA0335 | hypothetical protein | + | + | + | + | - |
| PA0337 | phosphoenolpyruvate-protein phosphotransferase PtsP | + | + | + | + | - |
| PA0341 | prolipoprotein diacylglyceryl transferase | - | - | - | - | + |
| PA0342 | thymidylate synthase | + | + | + | + | - |
| PA0349 | hypothetical protein | - | - | - | - | + |
| PA0350 | dihydrofolate reductase | + | + | + | + | - |
| PA0352 | probable transporter | - | - | - | - | + |
| PA0353 | dihydroxy-acid dehydratase | + | + | + | + | + |
| PA0355 | protease PfpI | + | + | + | + | - |
| PA0360 | hypothetical protein | - | - | - | - | + |
| PA0361 | probable gamma-glutamyltranspeptidase precursor | + | + | + | + | - |
| PA0363 | phosphopantetheine adenylyltransferase | + | - | - | - | - |
| PA0365 | hypothetical protein | - | - | - | - | + |
| PA0366 | probable aldehyde dehydrogenase | + | + | + | + | - |
| PA0371 | hypothetical protein | - | - | - | - | + |
| PA0372 | probable zinc protease | + | + | + | + | + |
| PA0373 | Signal recognition particle receptor protein FtsY (=alpha subunit) (TC 3.A.5.1.1) | + | + | + | + | - |
| PA0380 | conserved hypothetical protein | - | - | - | - | + |
| PA0381 | thiamine biosynthesis protein, thiazole moiety | + | + | + | + | + |
| PA0382 | DNA mismatch repair protein MicA | + | + | + | + | - |
| PA0386 | probable oxidase | - | - | - | - | + |
| PA0387 | conserved hypothetical protein | + | + | + | + | + |
| PA0388 | hypothetical protein | + | + | + | + | - |
| PA0392 | conserved hypothetical protein | - | - | - | - | + |
| PA0393 | pyrroline-5-carboxylate reductase | + | + | + | + | + |
| PA0395 | twitching motility protein PilT | + | + | + | + | - |
| PA0398 | hypothetical protein | - | - | - | - | + |
| PA0399 | cystathionine beta-synthase | + | + | + | + | + |
| PA0400 | probable cystathionine gamma-lyase | + | + | + | + | + |
| PA0401 | noncatalytic dihydroorotase-like protein | + | + | + | + | + |
| PA0402 | aspartate carbamoyltransferase | + | + | + | + | + |
| PA0403 | transcriptional regulator PyrR | + | + | + | + | - |
| PA0406 | TonB3 | - | - | - | - | + |
| PA0407 | glutathione synthetase | + | + | + | + | + |
| PA0408 | twitching motility protein PilG | + | + | + | + | + |
| PA0409 | twitching motility protein PilH | - | - | + | + | - |
| PA0410 | twitching motility protein PilI | - | - | - | - | + |
| PA0411 | twitching motility protein PilJ | + | + | + | + | - |
| PA0412 | methyltransferase PilK | - | - | - | - | + |
| PA0413 | component of chemotactic signal transduction system | - | + | + | + | - |
| PA0418 | hypothetical protein | + | - | - | - | - |
| PA0419 | conserved hypothetical protein | - | - | - | - | + |
| PA0420 | adenosylmethionine-8-amino-7-oxononanoate aminotransferase | + | + | + | + | + |
| PA0421 | hypothetical protein | + | + | + | + | - |
| PA0422 | conserved hypothetical protein | - | - | - | - | + |
| PA0423 | PasP | + | + | + | + | - |
| PA0424 | multidrug resistance operon repressor MexR | - | - | - | - | + |
| PA0425 | Resistance-Nodulation-Cell Division (RND) multidrug efflux membrane fusion protein MexA precursor | + | + | + | + | + |
| PA0427 | Major intrinsic multiple antibiotic resistance efflux outer membrane protein OprM precursor | + | + | + | + | + |
| PA0429 | hypothetical protein | - | - | - | - | + |
| PA0430 | 5,10-methylenetetrahydrofolate reductase | + | + | + | + | - |
| PA0431 | hypothetical protein | - | - | - | - | + |
| PA0432 | S-adenosyl-L-homocysteine hydrolase | + | + | + | + | - |
| PA0436 | probable transcriptional regulator | - | - | - | - | + |
| PA0437 | cytosine deaminase | + | + | + | + | - |
| PA0443 | probable transporter | - | - | - | - | + |
| PA0444 | N-carbamoyl-beta-alanine amidohydrolase | + | + | + | + | - |
| PA0445 | probable transposase | - | - | - | - | + |
| PA0446 | conserved hypothetical protein | + | + | + | + | + |
| PA0447 | glutaryl-CoA dehydrogenase | + | + | + | + | - |
| PA0448 | probable transcriptional regulator | - | - | - | - | + |
| PA0454 | conserved hypothetical protein | - | - | - | - | + |
| PA0455 | RNA helicase DbpA | + | + | + | + | + |
| PA0456 | probable cold-shock protein | + | + | + | + | - |
| PA0458 | probable major facilitator superfamily (MFS) transporter | - | - | - | - | + |
| PA0459 | probable ClpA/B protease ATP binding subunit | + | + | + | + | - |
| PA0461 | conserved hypothetical protein | - | - | - | - | + |
| PA0462 | hypothetical protein | + | + | + | + | + |
| PA0463 | two-component response regulator CreB | + | + | + | + | - |
| PA0467 | conserved hypothetical protein | - | - | - | - | + |
| PA0468 | hypothetical protein | + | + | + | + | + |
| PA0469 | hypothetical protein | + | + | + | + | - |
| PA0472 | FiuI | - | - | - | - | + |
| PA0473 | probable glutathione S-transferase | - | + | + | + | - |
| PA0477 | probable transcriptional regulator | - | - | - | - | + |
| PA0478 | probable N-acetyltransferase | - | + | + | - | - |
| PA0481 | hypothetical protein | - | - | - | - | + |
| PA0482 | malate synthase G | + | + | + | + | - |
| PA0486 | conserved hypothetical protein | + | - | - | - | - |
| PA0494 | probable acyl-CoA carboxylase subunit | - | - | + | - | - |
| PA0496 | conserved hypothetical protein | + | + | + | + | - |
| PA0499 | probable pili assembly chaperone | - | - | - | - | + |
| PA0500 | biotin synthase | + | + | + | + | + |
| PA0501 | 8-amino-7-oxononanoate synthase | - | - | - | - | + |
| PA0502 | probable biotin biosynthesis protein bioH | - | - | + | + | - |
| PA0504 | dethiobiotin synthase | + | - | - | - | - |
| PA0505 | hypothetical protein | - | - | - | - | + |
| PA0506 | probable acyl-CoA dehydrogenase | + | + | + | + | + |
| PA0507 | probable acyl-CoA dehydrogenase | + | + | + | + | + |
| PA0508 | probable acyl-CoA dehydrogenase | + | + | + | + | + |
| PA0509 | NirN | + | + | + | + | - |
| PA0514 | heme d1 biosynthesis protein NirL | - | - | - | - | + |
| PA0515 | probable transcriptional regulator | + | + | + | + | - |
| PA0517 | probable c-type cytochrome precursor | - | - | - | - | + |
| PA0518 | cytochrome c-551 precursor | + | + | + | + | + |
| PA0519 | nitrite reductase precursor | + | + | + | + | + |
| PA0520 | regulatory protein NirQ | + | + | + | + | - |
| PA0526 | hypothetical protein | - | - | - | - | + |
| PA0527 | transcriptional regulator Dnr | + | + | + | + | - |
| PA0536 | hypothetical protein | - | - | - | - | + |
| PA0541 | hypothetical protein | - | - | - | - | + |
| PA0542 | conserved hypothetical protein | + | + | + | + | - |
| PA0545 | hypothetical protein | - | - | - | - | + |
| PA0546 | methionine adenosyltransferase | - | + | + | + | - |
| PA0547 | probable transcriptional regulator | - | - | - | - | + |
| PA0548 | transketolase | + | + | + | + | - |
| PA0549 | hypothetical protein | - | - | - | - | + |
| PA0550 | conserved hypothetical protein | + | + | + | + | + |
| PA0551 | D-erythrose 4-phosphate dehydrogenase | + | + | + | + | + |
| PA0552 | phosphoglycerate kinase | + | + | + | + | - |
| PA0554 | hypothetical protein | - | - | - | - | + |
| PA0555 | fructose-1,6-bisphosphate aldolase | + | + | + | + | - |
| PA0557 | hypothetical protein | - | - | - | - | + |
| PA0558 | conserved hypothetical protein | + | + | + | + | - |
| PA0561 | hypothetical protein | - | - | - | - | + |
| PA0562 | probable hydrolase | + | + | + | + | - |
| PA0567 | conserved hypothetical protein | - | - | - | - | + |
| PA0575 | conserved hypothetical protein | - | - | - | - | + |
| PA0576 | sigma factor RpoD | + | + | + | + | - |
| PA0578 | conserved hypothetical protein | - | - | - | - | + |
| PA0579 | 30S ribosomal protein S21 | + | + | + | + | - |
| PA0583 | hypothetical protein | - | - | - | - | + |
| PA0584 | tRNA nucleotidyl transferase | + | + | + | + | - |
| PA0585 | hypothetical protein | - | - | - | - | + |
| PA0586 | conserved hypothetical protein | + | + | + | + | + |
| PA0587 | conserved hypothetical protein | + | + | + | + | + |
| PA0588 | conserved hypothetical protein | + | + | + | + | - |
| PA0589 | conserved hypothetical protein | - | - | - | - | + |
| PA0590 | bis(5'-nucleosyl)-tetraphosphatase | + | + | + | + | - |
| PA0591 | conserved hypothetical protein | - | - | - | - | + |
| PA0592 | rRNA (adenine-N6,N6)-dimethyltransferase | + | + | + | + | + |
| PA0593 | pyridoxal phosphate biosynthetic protein PdxA | + | + | + | + | + |
| PA0594 | peptidyl-prolyl cis-trans isomerase SurA | + | + | + | + | - |
| PA0595 | organic solvent tolerance protein OstA precursor | - | - | - | - | + |
| PA0596 | hypothetical protein | + | + | + | + | + |
| PA0597 | probable nucleotidyl transferase | + | + | + | + | - |
| PA0598 | hypothetical protein | - | - | - | - | + |
| PA0599 | hypothetical protein | + | + | + | + | - |
| PA0600 | two-component sensor, AgtS | - | - | - | - | + |
| PA0601 | two-component response regulator, AgtR | + | + | + | + | + |
| PA0602 | probable binding protein component of ABC transporter | + | + | + | + | - |
| PA0603 | AgtA | - | - | - | - | + |
| PA0604 | AgtB | + | + | + | + | - |
| PA0606 | AgtD | - | - | - | - | + |
| PA0607 | ribulose-phosphate 3-epimerase | + | + | + | + | - |
| PA0608 | probable phosphoglycolate phosphatase | - | - | - | - | + |
| PA0609 | anthranilate synthetase component I | + | + | + | + | - |
| PA0619 | probable bacteriophage protein | - | - | - | - | + |
| PA0620 | probable bacteriophage protein | + | - | - | - | - |
| PA0621 | conserved hypothetical protein | - | - | - | - | + |
| PA0622 | probable bacteriophage protein | + | + | + | + | - |
| PA0648 | hypothetical protein | - | - | - | - | + |
| PA0649 | anthranilate synthase component II | + | + | + | + | + |
| PA0650 | anthranilate phosphoribosyltransferase | + | + | + | + | + |
| PA0651 | indole-3-glycerol-phosphate synthase | + | + | + | + | + |
| PA0652 | transcriptional regulator Vfr | + | + | + | + | - |
| PA0653 | conserved hypothetical protein | - | - | - | - | + |
| PA0654 | S-adenosylmethionine decarboxylase proenzyme | + | + | + | + | - |
| PA0659 | hypothetical protein | - | - | - | - | + |
| PA0660 | hypothetical protein | + | + | + | + | - |
| PA0661 | conserved hypothetical protein | - | - | - | - | + |
| PA0662 | N-acetyl-gamma-glutamyl-phosphate reductase | + | + | + | + | - |
| PA0667 | conserved hypothetical protein | - | - | - | - | + |
| PA0668 | tyrosyl-tRNA synthetase 2 | + | + | + | + | - |
| PA0712 | hypothetical protein | - | - | - | - | + |
| PA0713 | hypothetical protein | - | + | - | + | - |
| PA0731 | hypothetical protein | - | - | - | - | + |
| PA0732 | hypothetical protein | + | + | + | + | - |
| PA0740 | SDS hydrolase SdsA1 | - | - | - | - | + |
| PA0741 | Rrf2-linked NADH-flavin reductase | + | + | + | + | - |
| PA0743 | probable 3-hydroxyisobutyrate dehydrogenase | - | - | - | - | + |
| PA0744 | probable enoyl-CoA hydratase/isomerase | + | + | + | + | + |
| PA0745 | probable enoyl-CoA hydratase/isomerase | + | + | + | + | + |
| PA0746 | probable acyl-CoA dehydrogenase | + | + | + | + | - |
| PA0747 | probable aldehyde dehydrogenase | + | - | - | - | - |
| PA0749 | hypothetical protein | - | - | - | - | + |
| PA0750 | uracil-DNA glycosylase | + | + | + | + | - |
| PA0753 | hypothetical protein | - | - | - | - | + |
| PA0754 | hypothetical protein | + | + | + | + | - |
| PA0757 | probable two-component sensor | - | - | - | - | + |
| PA0758 | hypothetical protein | + | + | + | + | + |
| PA0759 | conserved hypothetical protein | + | + | + | + | - |
| PA0761 | L-aspartate oxidase | - | - | - | - | + |
| PA0762 | sigma factor AlgU | + | + | + | + | - |
| PA0763 | anti-sigma factor MucA | - | - | - | - | + |
| PA0764 | negative regulator for alginate biosynthesis MucB | + | + | + | + | - |
| PA0766 | serine protease MucD precursor | - | - | - | - | + |
| PA0767 | GTP-binding protein LepA | + | + | + | + | + |
| PA0769 | hypothetical protein | - | - | - | - | + |
| PA0770 | ribonuclease III | + | + | + | + | + |
| PA0771 | GTP-binding protein Era | + | + | + | + | - |
| PA0772 | DNA repair protein RecO | - | - | - | - | + |
| PA0773 | pyridoxal phosphate biosynthetic protein PdxJ | + | + | + | + | - |
| PA0775 | conserved hypothetical protein | + | - | - | - | - |
| PA0778 | inhibitor of cysteine peptidase | - | - | - | - | + |
| PA0779 | AsrA | - | + | + | + | - |
| PA0781 | hypothetical protein | - | - | - | - | + |
| PA0782 | proline dehydrogenase PutA | + | + | + | + | - |
| PA0791 | probable transcriptional regulator | - | - | - | - | + |
| PA0792 | propionate catabolic protein PrpD | + | + | + | + | + |
| PA0795 | citrate synthase 2 | + | + | + | + | + |
| PA0796 | carboxyphosphonoenolpyruvate phosphonomutase | + | + | + | + | + |
| PA0797 | probable transcriptional regulator | + | + | + | + | - |
| PA0803 | hypothetical protein | - | - | - | - | + |
| PA0804 | probable oxidoreductase | + | + | + | + | - |
| PA0806 | hypothetical protein | - | - | - | - | + |
| PA0807 | AmpDh3 | + | + | + | + | + |
| PA0808 | hypothetical protein | - | + | + | + | - |
| PA0817 | probable ring-cleaving dioxygenase | - | + | - | - | - |
| PA0831 | transcriptional regulator OruR | - | - | - | - | + |
| PA0832 | conserved hypothetical protein | + | + | + | + | + |
| PA0833 | hypothetical protein | + | + | + | + | - |
| PA0834 | conserved hypothetical protein | - | - | - | - | + |
| PA0835 | phosphate acetyltransferase | + | + | + | + | + |
| PA0836 | acetate kinase | + | + | + | + | + |
| PA0837 | peptidyl-prolyl cis-trans isomerase SlyD | - | + | + | + | - |
| PA0838 | probable glutathione peroxidase | + | - | - | - | - |
| PA0839 | probable transcriptional regulator | - | - | - | - | + |
| PA0840 | probable oxidoreductase | + | + | + | + | - |
| PA0848 | alkyl hydroperoxide reductase , AhpB | + | + | + | + | + |
| PA0850 | hypothetical protein | - | - | - | - | + |
| PA0851 | hypothetical protein | + | + | - | - | + |
| PA0852 | chitin-binding protein CbpD precursor | + | + | + | + | + |
| PA0853 | probable oxidoreductase | + | + | + | + | + |
| PA0854 | fumarate hydratase | + | + | + | + | - |
| PA0855 | hypothetical protein | - | - | - | - | + |
| PA0856 | hypothetical protein | + | + | + | + | - |
| PA0857 | morphogene protein BolA | - | - | - | - | + |
| PA0858 | conserved hypothetical protein | + | + | + | + | - |
| PA0862 | hypothetical protein | - | - | - | - | + |
| PA0863 | probable oxidoreductase | - | - | + | - | - |
| PA0864 | probable transcriptional regulator | - | - | - | - | + |
| PA0865 | 4-hydroxyphenylpyruvate dioxygenase | + | + | + | + | - |
| PA0866 | aromatic amino acid transport protein AroP2 | - | - | - | - | + |
| PA0867 | membrane-bound lysozyme inhibitor of c-type lysozyme MliC | + | + | + | + | - |
| PA0869 | D-alanyl-D-alanine-endopeptidase | - | - | - | - | + |
| PA0870 | aromatic amino acid aminotransferase | + | + | + | + | + |
| PA0871 | pterin-4-alpha-carbinolamine dehydratase | + | + | + | + | + |
| PA0872 | phenylalanine-4-hydroxylase | + | + | + | + | - |
| PA0875 | conserved hypothetical protein | - | - | - | - | + |
| PA0877 | probable transcriptional regulator | - | - | - | - | + |
| PA0878 | hypothetical protein | - | + | + | + | - |
| PA0879 | probable acyl-CoA dehydrogenase | - | - | - | - | + |
| PA0880 | probable ring-cleaving dioxygenase | + | + | + | + | - |
| PA0883 | probable acyl-CoA lyase beta chain | - | - | - | - | + |
| PA0884 | probable C4-dicarboxylate-binding periplasmic protein | - | + | + | + | - |
| PA0886 | probable C4-dicarboxylate transporter | - | - | - | - | + |
| PA0887 | acetyl-coenzyme A synthetase | + | + | + | + | + |
| PA0888 | arginine/ornithine binding protein AotJ | + | + | + | + | - |
| PA0890 | arginine/ornithine transport protein AotM | - | - | - | - | + |
| PA0891 | hypothetical protein | + | + | + | + | - |
| PA0894 | hypothetical protein | - | - | - | - | + |
| PA0895 | N-succinylglutamate 5-semialdehyde dehydrogenase | + | + | + | + | + |
| PA0896 | arginine/ornithine succinyltransferase AI subunit | + | + | + | + | + |
| PA0897 | arginine/ornithine succinyltransferase AII subunit | + | + | + | + | + |
| PA0898 | succinylglutamate 5-semialdehyde dehydrogenase | + | + | + | + | + |
| PA0899 | succinylarginine dihydrolase | + | + | + | + | + |
| PA0900 | hypothetical protein | + | + | + | + | + |
| PA0903 | alanyl-tRNA synthetase | + | + | + | + | + |
| PA0904 | aspartate kinase alpha and beta chain | - | - | - | - | + |
| PA0905 | RsmA | + | + | + | + | - |
| PA0915 | conserved hypothetical protein | + | - | - | - | + |
| PA0916 | conserved hypothetical protein | + | + | + | + | - |
| PA0925 | hypothetical protein | - | - | - | - | + |
| PA0926 | hypothetical protein | + | + | + | + | - |
| PA0931 | ferric enterobactin receptor PirA | - | - | - | - | + |
| PA0932 | cysteine synthase B | + | + | + | + | - |
| PA0933 | probable RNA methyltransferase | - | - | - | - | + |
| PA0934 | GTP pyrophosphokinase | + | + | + | + | + |
| PA0935 | conserved hypothetical protein | + | + | + | + | + |
| PA0936 | lipopolysaccharide biosynthetic protein LpxO2 | + | + | + | + | + |
| PA0937 | conserved hypothetical protein | - | - | - | + | - |
| PA0940 | hypothetical protein | - | - | - | - | + |
| PA0941 | hypothetical protein | + | + | + | + | - |
| PA0942 | probable transcriptional regulator | - | - | - | - | + |
| PA0943 | hypothetical protein | + | + | + | + | + |
| PA0944 | phosphoribosylaminoimidazole synthetase | + | + | + | + | + |
| PA0945 | phosphoribosylaminoimidazole synthetase | + | + | + | + | - |
| PA0946 | hypothetical protein | - | - | - | - | + |
| PA0947 | conserved hypothetical protein | + | + | + | + | - |
| PA0949 | Trp repressor binding protein WrbA | - | - | - | - | + |
| PA0950 | probable arsenate reductase | + | + | + | + | - |
| PA0953 | probable thioredoxin | - | - | - | - | + |
| PA0955 | hypothetical protein | - | - | - | - | + |
| PA0956 | prolyl-tRNA synthetase | + | + | + | + | - |
| PA0957 | hypothetical protein | - | - | - | - | + |
| PA0958 | Basic amino acid, basic peptide and imipenem outer membrane porin OprD precursor | + | + | + | + | + |
| PA0961 | probable cold-shock protein | + | + | + | + | + |
| PA0962 | DNA-binding protein from starved cells, Dps | + | + | + | + | + |
| PA0963 | aspartyl-tRNA synthetase | + | + | + | + | + |
| PA0964 | pqsR-mediated PQS regulator, PmpR | + | + | + | + | - |
| PA0968 | conserved hypothetical protein | - | - | - | - | + |
| PA0969 | TolQ protein | + | + | + | + | - |
| PA0971 | TolA protein | - | - | - | - | + |
| PA0972 | TolB protein | + | + | + | + | + |
| PA0973 | Peptidoglycan associated lipoprotein OprL precursor | + | + | + | + | + |
| PA0974 | conserved hypothetical protein | - | + | + | + | - |
| PA0980 | hypothetical protein | - | - | - | - | + |
| PA0995 | methylated-DNA--protein-cysteine methyltransferase | - | - | - | - | + |
| PA0996 | probable coenzyme A ligase | + | + | + | + | + |
| PA0997 | PqsB | + | + | + | + | + |
| PA0998 | PqsC | + | + | + | + | + |
| PA0999 | 3-oxoacyl-[acyl-carrier-protein] synthase III | + | + | + | + | + |
| PA1000 | Quinolone signal response protein | + | + | + | + | - |
| PA1002 | anthranilate synthase component II | + | - | - | - | - |
| PA1003 | Transcriptional regulator MvfR | - | - | - | - | + |
| PA1004 | quinolinate synthetase A | + | + | + | + | + |
| PA1005 | conserved hypothetical protein | + | + | + | + | - |
| PA1007 | conserved hypothetical protein | - | - | - | - | + |
| PA1008 | bacterioferritin comigratory protein | + | + | + | + | + |
| PA1009 | hypothetical protein | + | + | + | + | + |
| PA1010 | dihydrodipicolinate synthase | + | + | + | + | + |
| PA1011 | hypothetical protein | + | + | + | + | - |
| PA1012 | conserved hypothetical protein | - | - | - | - | + |
| PA1013 | phosphoribosylaminoimidazole-succinocarboxamide synthase | + | + | + | + | - |
| PA1032 | QuiP | - | - | - | - | + |
| PA1033 | Glutathione S-transferase (EC 2.5.1.18) | + | + | + | + | - |
| PA1040 | hypothetical protein | - | - | - | - | + |
| PA1041 | probable outer membrane protein precursor | + | + | + | + | - |
| PA1042 | conserved hypothetical protein | - | - | - | - | + |
| PA1043 | hypothetical protein | + | + | + | + | - |
| PA1046 | hypothetical protein | - | - | - | - | + |
| PA1047 | probable esterase | + | + | + | + | - |
| PA1048 | probable outer membrane protein precursor | - | - | - | - | + |
| PA1049 | pyridoxine 5'-phosphate oxidase | + | + | + | + | - |
| PA1052 | conserved hypothetical protein | - | - | - | - | + |
| PA1053 | conserved hypothetical protein | + | + | + | + | - |
| PA1060 | hypothetical protein | - | - | - | - | + |
| PA1065 | conserved hypothetical protein | - | - | - | - | + |
| PA1066 | probable short-chain dehydrogenase | - | + | - | + | - |
| PA1067 | probable transcriptional regulator | - | - | - | - | + |
| PA1068 | probable heat shock protein (hsp90 family) | + | + | + | + | + |
| PA1069 | hypothetical protein | + | + | + | + | - |
| PA1073 | branched-chain amino acid transport protein BraD | - | - | - | - | + |
| PA1074 | branched-chain amino acid transport protein BraC | + | + | + | + | - |
| PA1075 | hypothetical protein | - | - | - | - | + |
| PA1076 | hypothetical protein | + | + | + | + | - |
| PA1079 | flagellar basal-body rod modification protein FlgD | - | - | - | - | + |
| PA1080 | flagellar hook protein FlgE | + | + | + | + | - |
| PA1085 | flagellar protein FlgJ | - | - | - | - | + |
| PA1086 | flagellar hook-associated protein 1 FlgK | + | - | + | - | - |
| PA1091 | flagellar glycosyl transferase, FgtA | - | - | - | - | + |
| PA1092 | flagellin type B | + | - | + | - | - |
| PA1093 | hypothetical protein | - | - | - | - | + |
| PA1094 | flagellar capping protein FliD | + | + | + | + | - |
| PA1096 | hypothetical protein | - | - | - | - | + |
| PA1097 | transcriptional regulator FleQ | + | + | + | + | - |
| PA1111 | hypothetical protein | - | - | - | - | + |
| PA1112 | conserved hypothetical protein | + | + | + | + | - |
| PA1121 | YfiR | - | - | - | - | + |
| PA1122 | probable peptide deformylase | + | + | + | + | + |
| PA1123 | hypothetical protein | + | + | + | + | - |
| PA1124 | deoxyguanosinetriphosphate triphosphohydrolase | + | - | - | - | - |
| PA1126 | hypothetical protein | - | - | - | - | + |
| PA1127 | probable oxidoreductase | + | + | + | + | - |
| PA1134 | hypothetical protein | - | - | - | - | + |
| PA1135 | conserved hypothetical protein | + | + | + | + | - |
| PA1138 | probable transcriptional regulator | - | - | - | - | + |
| PA1140 | conserved hypothetical protein | + | + | + | + | - |
| PA1154 | conserved hypothetical protein | - | - | - | - | + |
| PA1155 | NrdB, tyrosyl radical-harboring component of class Ia ribonucleotide reductase | + | + | + | + | + |
| PA1156 | NrdA, catalytic component of class Ia ribonucleotide reductase | + | + | + | + | - |
| PA1158 | probable two-component sensor | - | - | - | - | + |
| PA1160 | hypothetical protein | - | - | - | - | + |
| PA1161 | rRNA methyltransferase | - | + | - | - | + |
| PA1162 | succinyl-diaminopimelate desuccinylase | + | + | + | + | - |
| PA1165 | PcpS | - | - | - | - | + |
| PA1166 | hypothetical protein | + | + | + | + | - |
| PA1170 | conserved hypothetical protein | - | - | - | - | + |
| PA1173 | cytochrome c-type protein NapB precursor | - | - | - | - | + |
| PA1174 | periplasmic nitrate reductase protein NapA | + | + | + | + | - |
| PA1177 | periplasmic nitrate reductase protein NapE | - | - | - | - | + |
| PA1178 | PhoP/Q and low Mg2+ inducible outer membrane protein H1 precursor | + | + | + | + | + |
| PA1179 | two-component response regulator PhoP | + | + | + | + | - |
| PA1199 | probable lipoprotein | - | - | - | - | + |
| PA1201 | probable transcriptional regulator | - | - | - | - | + |
| PA1202 | probable hydrolase | + | + | + | + | + |
| PA1203 | hypothetical protein | + | + | + | + | + |
| PA1204 | NAD(P)H quinone oxidoreductase | + | + | + | + | + |
| PA1205 | conserved hypothetical protein | + | + | + | + | + |
| PA1206 | hypothetical protein | + | + | + | + | - |
| PA1215 | hypothetical protein | - | - | - | - | + |
| PA1216 | hypothetical protein | + | + | + | + | - |
| PA1224 | probable NAD(P)H dehydrogenase | - | - | - | - | + |
| PA1225 | probable NAD(P)H dehydrogenase | + | + | + | + | - |
| PA1233 | hypothetical protein | - | - | - | - | + |
| PA1234 | hypothetical protein | + | + | + | + | - |
| PA1243 | probable sensor/response regulator hybrid | - | - | - | - | + |
| PA1244 | hypothetical protein | + | + | + | + | - |
| PA1249 | alkaline metalloproteinase precursor | - | - | - | - | + |
| PA1250 | alkaline proteinase inhibitor AprI | + | + | + | + | - |
| PA1270 | hypothetical protein | - | - | - | - | + |
| PA1271 | probable tonB-dependent receptor | + | + | + | - | + |
| PA1272 | cob(I)alamin adenosyltransferase | + | + | + | + | - |
| PA1275 | cobalamin biosynthetic protein CobD | - | - | - | - | + |
| PA1276 | L-threonine 3-O-phosphate decarboxylase (EC 4.1.1.81) | + | + | + | + | - |
| PA1277 | cobyric acid synthase | - | - | - | - | + |
| PA1284 | probable acyl-CoA dehydrogenase | - | - | - | - | + |
| PA1286 | probable major facilitator superfamily (MFS) transporter | - | - | - | - | + |
| PA1287 | probable glutathione peroxidase | + | + | + | + | + |
| PA1288 | probable outer membrane protein precursor | + | + | + | + | - |
| PA1291 | hypothetical protein | - | - | - | - | + |
| PA1292 | probable 3-mercaptopyruvate sulfurtransferase | + | + | + | + | - |
| PA1293 | hypothetical protein | - | - | - | - | + |
| PA1294 | ribonuclease D | + | + | + | + | - |
| PA1295 | conserved hypothetical protein | - | - | - | - | + |
| PA1296 | probable 2-hydroxyacid dehydrogenase | + | + | + | + | - |
| PA1303 | signal peptidase | + | + | + | + | + |
| PA1304 | probable oligopeptidase | + | + | + | + | - |
| PA1306 | probable HIT family protein | - | - | - | - | + |
| PA1307 | conserved hypothetical protein | + | + | + | + | - |
| PA1323 | hypothetical protein | - | - | - | - | + |
| PA1324 | hypothetical protein | + | + | + | + | - |
| PA1329 | conserved hypothetical protein | - | - | - | - | + |
| PA1330 | probable short-chain dehydrogenase | + | + | + | + | - |
| PA1336 | AauS | - | - | - | - | + |
| PA1337 | glutaminase-asparaginase | + | + | + | + | + |
| PA1338 | gamma-glutamyltranspeptidase precursor | + | + | + | + | - |
| PA1341 | AatQ | - | - | - | - | + |
| PA1342 | AatJ | - | + | + | + | - |
| PA1343 | hypothetical protein | - | - | - | - | + |
| PA1344 | probable short-chain dehydrogenase | + | + | + | + | - |
| PA1371 | hypothetical protein | - | - | - | - | + |
| PA1372 | hypothetical protein | - | - | - | - | + |
| PA1373 | 3-oxoacyl-acyl carrier protein synthase II | + | + | + | + | - |
| PA1375 | erythronate-4-phosphate dehydrogenase | - | - | + | + | - |
| PA1420 | hypothetical protein | - | - | - | - | + |
| PA1421 | guanidinobutyrase | + | + | + | + | - |
| PA1429 | probable cation-transporting P-type ATPase | - | - | - | - | + |
| PA1439 | conserved hypothetical protein | - | - | - | - | + |
| PA1440 | hypothetical protein | + | + | + | + | - |
| PA1442 | conserved hypothetical protein | - | - | - | - | + |
| PA1443 | flagellar motor switch protein FliM | + | + | + | + | + |
| PA1444 | flagellar motor switch protein FliN | + | + | + | + | - |
| PA1453 | flagellar biosynthesis protein FlhF | - | - | - | - | + |
| PA1454 | flagellar synthesis regulator FleN | + | + | + | + | - |
| PA1455 | sigma factor FliA | - | + | - | - | - |
| PA1458 | probable two-component sensor | - | - | - | - | + |
| PA1459 | probable methyltransferase | + | - | - | + | - |
| PA1463 | hypothetical protein | - | - | - | - | + |
| PA1464 | probable purine-binding chemotaxis protein | + | + | + | + | - |
| PA1469 | hypothetical protein | - | - | - | - | + |
| PA1470 | probable short-chain dehydrogenase | + | + | + | + | - |
| PA1478 | hypothetical protein | - | - | - | - | + |
| PA1479 | cytochrome C-type biogenesis protein CcmE | - | + | + | + | - |
| PA1482 | cytochrome C-type biogenesis protein CcmH | - | - | - | - | + |
| PA1483 | cytochrome c-type biogenesis protein | - | - | + | + | - |
| PA1492 | hypothetical protein | - | - | - | - | + |
| PA1493 | sulfate-binding protein of ABC transporter | + | + | + | + | + |
| PA1494 | mucoidy inhibitor gene A | + | + | + | + | - |
| PA1515 | allantoicase | - | - | - | - | + |
| PA1516 | hypothetical protein | + | + | + | + | + |
| PA1517 | conserved hypothetical protein | + | + | + | + | + |
| PA1518 | conserved hypothetical protein | - | + | + | + | - |
| PA1519 | probable transporter | - | - | - | - | + |
| PA1525 | alkane-1-monooxygenase 2 | - | - | - | - | + |
| PA1526 | probable transcriptional regulator | + | + | + | + | - |
| PA1527 | conserved hypothetical protein | - | - | - | - | + |
| PA1528 | cell division protein ZipA | + | + | + | + | + |
| PA1529 | DNA ligase | + | + |  |  | - |
| PA1543 | adenine phosphoribosyltransferase | - | - | - | - | + |
| PA1544 | transcriptional regulator Anr | + | + | + | + | - |
| PA1545 | hypothetical protein | - | - | - | - | + |
| PA1546 | oxygen-independent coproporphyrinogen III oxidase | + | + | + | + | - |
| PA1549 | probable cation-transporting P-type ATPase | - | - | - | - | + |
| PA1550 | hypothetical protein | + | + | + | + | + |
| PA1551 | probable ferredoxin | + | + | + | + | + |
| PA1552 | Cytochrome c oxidase, cbb3-type, CcoP subunit | - | + | + | + | - |
| PA1552.1 | Cytochrome c oxidase, cbb3-type, CcoQ subunit | - | - | - | - | + |
| PA1553 | Cytochrome c oxidase, cbb3-type, CcoO subunit | + | + | + | + | - |
| PA1554 | Cytochrome c oxidase, cbb3-type, CcoN subunit | - | - | - | - | + |
| PA1555 | Cytochrome c oxidase, cbb3-type, CcoP subunit | + | + | + | + | - |
| PA1555.1 | Cytochrome c oxidase, cbb3-type, CcoQ subunit | - | - | - | - | + |
| PA1556 | Cytochrome c oxidase, cbb3-type, CcoO subunit | + | + | + | + | - |
| PA1560 | hypothetical protein | - | - | - | - | + |
| PA1561 | aerotaxis receptor Aer | - | - | - | + | + |
| PA1562 | aconitate hydratase 1 | + | + | + | + | + |
| PA1563 | conserved hypothetical protein | - | - | + | + | - |
| PA1573 | conserved hypothetical protein | - | - | - | - | + |
| PA1574 | conserved hypothetical protein | + | + | + | + | - |
| PA1575 | hypothetical protein | - | - | - | - | + |
| PA1576 | probable 3-hydroxyisobutyrate dehydrogenase | + | + | + | + | - |
| PA1578 | hypothetical protein | - | - | - | - | + |
| PA1579 | hypothetical protein | + | + | + | + | + |
| PA1580 | citrate synthase | + | + | + | + | - |
| PA1582 | succinate dehydrogenase (D subunit) | - | - | - | - | + |
| PA1583 | succinate dehydrogenase (A subunit) | + | + | + | + | + |
| PA1584 | succinate dehydrogenase (B subunit) | + | + | + | + | + |
| PA1585 | 2-oxoglutarate dehydrogenase (E1 subunit) | + | + | + | + | + |
| PA1586 | dihydrolipoamide succinyltransferase (E2 subunit) | + | + | + | + | + |
| PA1587 | dihydrolipoamide dehydrogenase Lpd | + | + | + | + | + |
| PA1588 | succinyl-CoA synthetase beta chain | + | + | + | + | + |
| PA1589 | succinyl-CoA synthetase alpha chain | + | + | + | + | - |
| PA1595 | hypothetical protein | - | - | - | - | + |
| PA1596 | heat shock protein HtpG | + | + | + | + | + |
| PA1597 | hypothetical protein | + | + | + | + | - |
| PA1600 | probable cytochrome c | + | - | - | - | + |
| PA1601 | probable aldehyde dehydrogenase | + | + | + | + | - |
| PA1603 | probable transcriptional regulator | - | - | - | - | + |
| PA1604 | hypothetical protein | + | + | + | + | + |
| PA1605 | hypothetical protein | + | + | + | + | - |
| PA1608 | probable chemotaxis transducer | - | - | + | - | + |
| PA1609 | beta-ketoacyl-ACP synthase I | + | + | + | + | + |
| PA1610 | beta-hydroxydecanoyl-ACP dehydrase | + | + | + | + | - |
| PA1613 | hypothetical protein | - | - | - | - | + |
| PA1614 | glycerol-3-phosphate dehydrogenase, biosynthetic | + | + | + | + | - |
| PA1615 | probable lipase | - | - | - | - | + |
| PA1616 | conserved hypothetical protein | + | + | + | + | - |
| PA1622 | probable hydrolase | - | - | - | - | + |
| PA1623 | conserved hypothetical protein | + | + | + | + | + |
| PA1624 | hypothetical protein | + | + | + | + | - |
| PA1641 | hypothetical protein | - | - | - | - | + |
| PA1642 | selenophosphate synthetase | + | + | + | + | - |
| PA1643 | conserved hypothetical protein | - | - | - | - | + |
| PA1644 | conserved hypothetical protein | + | + | + | + | - |
| PA1645 | hypothetical protein | + | - | - | - | - |
| PA1647 | probable sulfate transporter | - | - | - | - | + |
| PA1648 | probable oxidoreductase | - | + | + | + | - |
| PA1653 | probable transcriptional regulator | - | - | - | - | + |
| PA1654 | probable aminotransferase | + | + | + | + | + |
| PA1655 | probable glutathione S-transferase | + | + | + | + | - |
| PA1656 | HsiA2 | - | - | - | - | + |
| PA1657 | HsiB2 | + | + | + | + | + |
| PA1658 | HsiC2 | + | + | + | + | - |
| PA1661 | HsiH2 | - | - | - | - | + |
| PA1662 | clpV2 | + | + | + | + | - |
| PA1672 | hypothetical protein | - | - | - | - | + |
| PA1673 | hypothetical protein | + | + | + | + | - |
| PA1676 | hypothetical protein | - | - | - | - | + |
| PA1680 | hypothetical protein | - | - | - | - | + |
| PA1681 | chorismate synthase | + | + | + | + | - |
| PA1683 | probable sugar aldolase | + | - | - | - | + |
| PA1685 | enolase-phosphatase E-1 | + | + | + | + | - |
| PA1687 | spermidine synthase | + | + | + | + | - |
| PA1728 | hypothetical protein | - | - | - | - | + |
| PA1729 | conserved hypothetical protein | + | + | + | + | - |
| PA1741 | hypothetical protein | - | - | - | - | + |
| PA1742 | Glutamine amidotransferase class I | + | + | + | + | - |
| PA1745 | hypothetical protein | - | - | - | - | + |
| PA1746 | hypothetical protein | + | + | + | + | - |
| PA1747 | hypothetical protein | - | - | - | - | + |
| PA1748 | probable enoyl-CoA hydratase/isomerase | + | + | + | + | + |
| PA1749 | hypothetical protein | + | + | + | + | + |
| PA1750 | phospho-2-dehydro-3-deoxyheptonate aldolase | + | + | + | + | - |
| PA1753 | conserved hypothetical protein | - | - | - | - | + |
| PA1754 | transcriptional regulator CysB | + | + | + | + | - |
| PA1755 | hypothetical protein | - | - | - | - | + |
| PA1757 | homoserine kinase | + | + | + | + | + |
| PA1758 | para-aminobenzoate synthase component I | + | + | + | + | - |
| PA1766 | hypothetical protein | - | - | - | - | + |
| PA1767 | hypothetical protein | + | + | + | + | + |
| PA1768 | hypothetical protein | + | + | + | + | + |
| PA1770 | phosphoenolpyruvate synthase | + | + | + | + | - |
| PA1771 | EstX | - | - | - | - | + |
| PA1772 | probable methyltransferase | + | + | + | + | - |
| PA1776 | ECF sigma factor SigX | - | - | - | - | + |
| PA1777 | Major porin and structural outer membrane porin OprF precursor | + | + | + | + | - |
| PA1786 | NasS | - | - | - | - | + |
| PA1787 | aconitate hydratase 2 | + | + | + | + | - |
| PA1788 | hypothetical protein | - | - | - | - | + |
| PA1789 | hypothetical protein | + | + | + | + | - |
| PA1792 | conserved hypothetical protein | - | - | - | - | + |
| PA1793 | peptidyl-prolyl cis-trans isomerase B | + | + | + | + | + |
| PA1794 | glutaminyl-tRNA synthetase | + | + | + | + | + |
| PA1795 | cysteinyl-tRNA synthetase | + | + | + | + | + |
| PA1796 | 5,10-methylene-tetrahydrofolate dehydrogenase / cyclohydrolase | + | + | + | + | - |
| PA1798 | two-component sensor, ParS | - | - | - | - | + |
| PA1799 | two-component response regulator, ParR | + | + | + | + | + |
| PA1800 | trigger factor | + | + | + | + | + |
| PA1801 | ClpP | + | + | + | + | + |
| PA1802 | ClpX | + | + | + | + | + |
| PA1803 | Lon protease | + | + | + | + | + |
| PA1804 | DNA-binding protein HU | + | + | + | + | + |
| PA1805 | peptidyl-prolyl cis-trans isomerase D | + | + | + | + | + |
| PA1809 | probable permease of ABC transporter | - | - | - | - | + |
| PA1810 | probable binding protein component of ABC transporter | + | + | + | + | - |
| PA1812 | membrane-bound lytic murein transglycosylase D precursor | - | - | - | - | + |
| PA1813 | probable hydroxyacylglutathione hydrolase | + | + | + | + | - |
| PA1814 | KerV | - | - | - | - | + |
| PA1817 | hypothetical protein | - | - | - | - | + |
| PA1818 | lysine-specific pyridoxal 5'-phosphate-dependent carboxylase, LdcA | + | + | + | + | - |
| PA1820 | sodium/proton antiporter NhaB | - | - | - | - | + |
| PA1821 | probable enoyl-CoA hydratase/isomerase | + | + | + | + | + |
| PA1822 | hypothetical protein | + | + | + | + | + |
| PA1827 | probable short-chain dehydrogenase | - | - | - | - | + |
| PA1828 | probable short-chain dehydrogenase | + | + | + | + | + |
| PA1829 | hypothetical protein | + | + | + | + | + |
| PA1830 | hypothetical protein | + | + | + | + | - |
| PA1832 | probable protease | - | - | - | - | + |
| PA1833 | probable oxidoreductase | - | + | + | + | - |
| PA1836 | probable transcriptional regulator | - | - | - | - | + |
| PA1837 | hypothetical protein | + | + | + | + | + |
| PA1838 | sulfite reductase | + | + | + | + | - |
| PA1841 | hypothetical protein | - | - | - | - | + |
| PA1842 | hypothetical protein | - | + | - | - | - |
| PA1845 | Tsi1 | + | + | + | + | + |
| PA1847 | NfuA | + | + | + | + | - |
| PA1851 | hypothetical protein | - | - | - | - | + |
| PA1852 | hypothetical protein | + | + | + | + | - |
| PA1862 | molybdenum transport protein ModB | - | - | - | - | + |
| PA1863 | molybdate-binding periplasmic protein precursor ModA | + | + | + | + | - |
| PA1879 | hypothetical protein | - | - | - | - | + |
| PA1880 | probable oxidoreductase | + | + | + | + | - |
| PA1889 | hypothetical protein | - | - | - | - | + |
| PA1890 | probable glutathione S-transferase | + | + | + | + | - |
| PA1898 | quorum-sensing control repressor | - | - | - | - | + |
| PA1913 | hypothetical protein | + | - | + | - | - |
| PA1926 | conserved hypothetical protein | - | - | - | - | + |
| PA1927 | 5-methyltetrahydropteroyltriglutamate-homocysteine S-methyltransferase | + | + | + | + | - |
| PA1933 | probable hydroxylase large subunit | - | - | - | - | + |
| PA1934 | hypothetical protein | + | + | + | + | - |
| PA1943 | hypothetical protein | - | - | - | - | + |
| PA1944 | hypothetical protein | + | + | + | + | - |
| PA1945 | probable transcriptional regulator | - | - | - | - | + |
| PA1946 | binding protein component precursor of ABC ribose transporter | + | + | + | + | - |
| PA1949 | ribose operon repressor RbsR | - | - | - | - | + |
| PA1950 | ribokinase | + | + | + | + | - |
| PA1965 | hypothetical protein | - | - | - | - | + |
| PA1966 | hypothetical protein | + | + | + | - | - |
| PA1968 | hypothetical protein | - | - | - | - | + |
| PA1969 | hypothetical protein | - | + | + | + | - |
| PA1985 | pyrroloquinoline quinone biosynthesis protein A | - | - | - | - | + |
| PA1998 | DhcR, transcriptional regulator | - | - | - | - | + |
| PA1999 | DhcA, dehydrocarnitine CoA transferase, subunit A | + | + | + | + | + |
| PA2000 | DhcB, dehydrocarnitine CoA transferase, subunit B | - | - | + | - | + |
| PA2001 | acetyl-CoA acetyltransferase | + | + | + | + | - |
| PA2002 | conserved hypothetical protein | - | - | - | - | + |
| PA2003 | 3-hydroxybutyrate dehydrogenase | + | + | + | + | - |
| PA2006 | probable major facilitator superfamily (MFS) transporter | - | - | - | - | + |
| PA2007 | maleylacetoacetate isomerase | + | + | + | + | + |
| PA2008 | fumarylacetoacetase | + | + | + | + | + |
| PA2009 | homogentisate 1,2-dioxygenase | + | + | + | + | + |
| PA2011 | 3-hydroxy-3-methylglutaryl-CoA lyase | + | + | + | + | + |
| PA2012 | methylcrotonyl-CoA carboxylase, alpha-subunit (biotin-containing) | - | - | - | + | - |
| PA2013 | putative 3-methylglutaconyl-CoA hydratase | - | - | - | - | + |
| PA2014 | methylcrotonyl-CoA carboxylase, beta-subunit | + | + | + | + | + |
| PA2015 | putative isovaleryl-CoA dehydrogenase | + | + | + | + | + |
| PA2016 | regulator of liu genes | + | + | + | + | - |
| PA2018 | Resistance-Nodulation-Cell Division (RND) multidrug efflux transporter MexY | - | - | - | - | + |
| PA2019 | Resistance-Nodulation-Cell Division (RND) multidrug efflux membrane fusion protein MexX precursor | + | + | + | + | - |
| PA2022 | probable nucleotide sugar dehydrogenase | - | - | - | - | + |
| PA2023 | UTP--glucose-1-phosphate uridylyltransferase | + | + | + | + | - |
| PA2024 | probable ring-cleaving dioxygenase | - | - | - | - | + |
| PA2025 | glutathione reductase | + | + | + | + | - |
| PA2039 | hypothetical protein | - | - | - | - | + |
| PA2043 | hypothetical protein | - | - | - | - | + |
| PA2044 | hypothetical protein | + | + | + | + | - |
| PA2068 | probable major facilitator superfamily (MFS) transporter | - | - | - | - | + |
| PA2069 | probable carbamoyl transferase | + | + | + | + | - |
| PA2070 | hypothetical protein | - | - | - | - | + |
| PA2071 | elongation factor G | + | + | + | + | - |
| PA2079 | probable amino acid permease | - | - | - | - | + |
| PA2080 | kynureninase KynU | + | + | + | + | + |
| PA2081 | kynurenine formamidase, KynB | + | + | + | + | - |
| PA2111 | hypothetical protein | - | - | - | - | + |
| PA2112 | conserved hypothetical protein | + | + | + | + | - |
| PA2115 | probable transcriptional regulator | - | - | - | - | + |
| PA2116 | conserved hypothetical protein | + | + | + | + | + |
| PA2117 | hypothetical protein | + | + | + | + | - |
| PA2118 | O6-methylguanine-DNA methyltransferase | - | - | - | - | + |
| PA2119 | alcohol dehydrogenase (Zn-dependent) | + | + | + | + | - |
| PA2133 | Cyclic-guanylate-specific phosphodiesterase | - | - | - | - | + |
| PA2134 | hypothetical protein | + | + | + | + | - |
| PA2146 | conserved hypothetical protein | - | - | - | - | + |
| PA2147 | catalase HPII | + | + | + | + | - |
| PA2170 | hypothetical protein | - | - | - | - | + |
| PA2171 | hypothetical protein | + | + | + | + | - |
| PA2183 | hypothetical protein | - | - | - | - | + |
| PA2193 | hydrogen cyanide synthase HcnA | - | - | - | - | + |
| PA2194 | hydrogen cyanide synthase HcnB | + | + | + | + | + |
| PA2195 | hydrogen cyanide synthase HcnC | + | + | + | + | - |
| PA2196 | TetR family transcriptional regulator | - | - | - | - | + |
| PA2197 | conserved hypothetical protein | + | + | + | + | - |
| PA2198 | hypothetical protein | - | - | - | - | + |
| PA2199 | probable dehydrogenase | + | + | + | + | - |
| PA2203 | probable amino acid permease | - | - | - | - | + |
| PA2204 | probable binding protein component of ABC transporter | + | + | + | + | - |
| PA2222 | hypothetical protein | - | - | - | - | + |
| PA2223 | hypothetical protein | + | - | + | - | - |
| PA2228 | hypothetical protein | - | - | - | - | + |
| PA2231 | PslA | - | - | - | - | + |
| PA2234 | PslD | - | - | - | - | + |
| PA2246 | transcriptional regulator BkdR | + | + | + | + | + |
| PA2252 | probable AGCS sodium/alanine/glycine symporter | - | - | - | - | + |
| PA2289 | conserved hypothetical protein | - | - | - | - | + |
| PA2290 | glucose dehydrogenase | + | - | - | + | - |
| PA2299 | probable transcriptional regulator | - | - | - | - | + |
| PA2301 | hypothetical protein | + | - | - | - | + |
| PA2303 | AmbD | - | - | - | - | + |
| PA2305 | AmbB | - | - | + | - | - |
| PA2322 | gluconate permease | - | - | - | - | + |
| PA2323 | probable glyceraldehyde-3-phosphate dehydrogenase | - | - | + | + | - |
| PA2327 | probable permease of ABC transporter | - | - | - | - | + |
| PA2329 | probable ATP-binding component of ABC transporter | - | - | - | + | + |
| PA2351 | probable permease of ABC transporter | - | - | - | - | + |
| PA2377 | hypothetical protein | - | - | - | - | + |
| PA2378 | probable aldehyde dehydrogenase | + | + | + | + | + |
| PA2385 | 3-oxo-C12-homoserine lactone acylase PvdQ | - | - | - | - | + |
| PA2386 | L-ornithine N5-oxygenase | - | - | - | + | - |
| PA2406 | hypothetical protein | - | - | - | - | + |
| PA2407 | probable adhesion protein | + | + | + | + | - |
| PA2409 | probable permease of ABC transporter | - | - | - | - | + |
| PA2410 | hypothetical protein | + | + | + | + | - |
| PA2431 | hypothetical protein | - | - | - | - | + |
| PA2432 | bistable expression regulator, BexR | + | + | + | + | + |
| PA2433 | hypothetical protein | + | + | + | + | - |
| PA2441 | hypothetical protein | - | - | - | - | + |
| PA2442 | glycine cleavage system protein T2 | + | + | + | + | + |
| PA2443 | L-serine dehydratase | - | + | + | + | - |
| PA2444 | serine hydroxymethyltransferase | + | + | + | + | + |
| PA2445 | glycine cleavage system protein P2 | + | + | + | + | + |
| PA2446 | glycine cleavage system protein H2 | - | + | + | + | - |
| PA2447 | probable transcriptional regulator | - | - | - | - | + |
| PA2448 | hypothetical protein | - | + | + | + | - |
| PA2452 | hypothetical protein | - | - | - | - | + |
| PA2453 | hypothetical protein | + | + | + | + | - |
| PA2461 | hypothetical protein | - | - | - | - | + |
| PA2463 | hypothetical protein | - | - | - | - | + |
| PA2464 | hypothetical protein | + | + | + | + | - |
| PA2477 | probable thiol:disulfide interchange protein | - | - | - | - | + |
| PA2482 | probable cytochrome c | + | + | + | + | + |
| PA2483 | conserved hypothetical protein | + | - | - | - | - |
| PA2490 | conserved hypothetical protein | - | - | - | - | + |
| PA2491 | MexS | + | + | + | + | - |
| PA2503 | hypothetical protein | - | - | - | + | + |
| PA2504 | hypothetical protein | + | + | + | + | - |
| PA2513 | anthranilate dioxygenase small subunit | - | - | - | - | + |
| PA2514 | anthranilate dioxygenase reductase | + | + | + | + | - |
| PA2529 | hypothetical protein | - | - | - | - | + |
| PA2530 | hypothetical protein | - | + | + | + | - |
| PA2531 | probable aminotransferase | - | - | - | - | + |
| PA2532 | thiol peroxidase | + | + | + | + | - |
| PA2544 | hypothetical protein | - | - | - | - | + |
| PA2545 | exodeoxyribonuclease III | + | + | + | + | + |
| PA2546 | probable ring-cleaving dioxygenase | + | + | + | + | - |
| PA2551 | probable transcriptional regulator | - | - | - | - | + |
| PA2552 | probable acyl-CoA dehydrogenase | + | + | + | + | + |
| PA2553 | probable acyl-CoA thiolase | + | + | + | + | + |
| PA2554 | probable short-chain dehydrogenase | + | + | + | + | - |
| PA2561 | CtpH |  | - | | + | + |
| PA2562 | hypothetical protein | + | + | + | + | - |
| PA2569 | hypothetical protein | - | - | - | - | + |
| PA2571 | probable two-component sensor | - | - | - | - | + |
| PA2572 | probable two-component response regulator | + | + | + | + | - |
| PA2574 | alkane-1-monooxygenase | - | - | - | - | + |
| PA2575 | hypothetical protein | + | + | + | + | - |
| PA2578 | probable acetyltransferase | - | - | - | - | + |
| PA2579 | L-Tryptophan:oxygen 2,3-oxidoreductase (decyclizing) KynA | + | + | + | + | - |
| PA2581 | hypothetical protein | - | - | - | - | + |
| PA2582 | hypothetical protein | + | + | + | + | - |
| PA2586 | response regulator GacA | - | - | - | + | + |
| PA2587 | probable FAD-dependent monooxygenase | + | + | + | + | - |
| PA2591 | VqsR |  | - | | + | + |
| PA2592 | probable periplasmic spermidine/putrescine-binding protein | + | + | + | + | - |
| PA2608 | conserved hypothetical protein | - | - | - | - | + |
| PA2609 | hypothetical protein | + | + | + | + | - |
| PA2610 | conserved hypothetical protein | - | - | - | - | + |
| PA2611 | siroheme synthase | + | + | + | + | + |
| PA2612 | seryl-tRNA synthetase | + | + | + | + | - |
| PA2613 | conserved hypothetical protein | - | - | - | - | + |
| PA2614 | periplasmic chaperone LolA | + | + | + | + | - |
| PA2615 | cell division protein FtsK | - | - | - | - | + |
| PA2616 | thioredoxin reductase 1 | + | + | + | + | - |
| PA2618 | hypothetical protein | - | - | - | - | + |
| PA2619 | initiation factor | + | + | + | + | + |
| PA2620 | ATP-binding protease component ClpA | + | + | + | + | - |
| PA2621 | ClpS | - | - | - | - | + |
| PA2622 | cold-shock protein CspD | + | + | + | + | + |
| PA2623 | isocitrate dehydrogenase | + | + | + | + | + |
| PA2624 | isocitrate dehydrogenase | + | + | + | + | + |
| PA2625 | conserved hypothetical protein | + | + | + | + | + |
| PA2626 | tRNA methyltransferase | + | + | + | + | - |
| PA2628 | hypothetical protein | - | - | - | - | + |
| PA2629 | adenylosuccinate lyase | + | + | + | + | - |
| PA2630 | conserved hypothetical protein | - | - | - | - | + |
| PA2631 | probable acetyl transferase | + | + | + | + | - |
| PA2632 | hypothetical protein | - | - | - | - | + |
| PA2634 | isocitrate lyase AceA | + | + | + | + | - |
| PA2637 | NADH dehydrogenase I chain A | - | + | - | - | + |
| PA2638 | NADH dehydrogenase I chain B | + | + | + | + | + |
| PA2639 | NADH dehydrogenase I chain C,D | + | + | + | + | - |
| PA2640 | NADH dehydrogenase I chain E | - | - | - | - | + |
| PA2641 | NADH dehydrogenase I chain F | + | + | + | + | + |
| PA2642 | NADH dehydrogenase I chain G | + | + | + | + | - |
| PA2644 | NADH Dehydrogenase I chain I | + | - | - | - | - |
| PA2658 | hypothetical protein | + | + | + | + | + |
| PA2663 | psl and pyoverdine operon regulator, PpyR | - | - | - | - | + |
| PA2664 | flavohemoprotein | + | + | + | + | - |
| PA2665 | Transcriptional activator of P. aeruginosa flavohemoglobin, FhpR | - | - | - | - | + |
| PA2667 | MvaU | + | + | - | - | + |
| PA2678 | probable permease of ABC-2 transporter | - | - | - | - | + |
| PA2679 | hypothetical protein | + | + | + | + | - |
| PA2697 | hypothetical protein | - | - | - | - | + |
| PA2698 | probable hydrolase | + | + | + | + | + |
| PA2699 | hypothetical protein | - | + | - | + | - |
| PA2708 | hypothetical protein | - | - | - | - | + |
| PA2709 | cysteine synthase A | + | + | + | + | - |
| PA2710 | hypothetical protein | - | - | - | - | + |
| PA2711 | probable periplasmic spermidine/putrescine-binding protein | + | + | + | + | - |
| PA2716 | probable FMN oxidoreductase | - | - | - | - | + |
| PA2717 | chloroperoxidase precursor | + | + | + | + | - |
| PA2720 | hypothetical protein | + | + | + | + | - |
| PA2728 | hypothetical protein | - | - | - | - | + |
| PA2729 | hypothetical protein | - | + | + | + | - |
| PA2734 | hypothetical protein | - | - | - | - | + |
| PA2736 | hypothetical protein | - | - | - | - | + |
| PA2737 | conserved hypothetical protein | + | + | + | + | + |
| PA2738 | integration host factor, alpha subunit | + | + | + | + | + |
| PA2739 | phenylalanyl-tRNA synthetase, beta subunit | + | + | + | + | + |
| PA2740 | phenylalanyl-tRNA synthetase, alpha-subunit | + | + | + | + | + |
| PA2741 | 50S ribosomal protein L20 | + | + | + | + | - |
| PA2743 | translation initiation factor IF-3 | - | - | - | - | + |
| PA2744 | threonyl-tRNA synthetase | + | + | + | + | + |
| PA2754 | conserved hypothetical protein | + | + | + | + | + |
| PA2755 | ecotin precursor | + | + | + | + | - |
| PA2759 | hypothetical protein | - | - | - | - | + |
| PA2760 | OprQ | + | + | + | - | - |
| PA2763 | hypothetical protein | - | - | - | - | + |
| PA2769 | hypothetical protein | - | - | - | - | + |
| PA2770 | hypothetical protein | + | + | + | + | + |
| PA2771 | conserved hypothetical protein | - | + | + | + | - |
| PA2775 | Tsi4 | - | - | - | - | + |
| PA2776 | FAD-dependent oxidoreductase | + | + | + | + | - |
| PA2786 | hypothetical protein | - | - | - | - | + |
| PA2787 | carboxypeptidase G2 precursor | + | - | + | - | - |
| PA2794 | pseudaminidase | - | - | - | - | + |
| PA2795 | conserved hypothetical protein | + | + | + | + | + |
| PA2796 | transaldolase | + | + | + | + | + |
| PA2797 | hypothetical protein | + | + | + | + | - |
| PA2799 | hypothetical protein | - | - | - | - | + |
| PA2801 | hypothetical protein | + | + | + | + | - |
| PA2805 | hypothetical protein | - | - | - | - | + |
| PA2806 | conserved hypothetical protein | + | + | + | + | - |
| PA2812 | probable ATP-binding component of ABC transporter | - | - | - | - | + |
| PA2813 | probable glutathione S-transferase | + | + | + | + | - |
| PA2814 | hypothetical protein | - | - | - | - | + |
| PA2815 | probable acyl-CoA dehydrogenase | + | + | + | + | - |
| PA2816 | hypothetical protein | - | - | - | - | + |
| PA2817 | hypothetical protein | - | + | - | + | - |
| PA2820 | hypothetical protein | - | - | - | - | + |
| PA2821 | probable glutathione S-transferase | + | + | + | + | + |
| PA2822 | conserved hypothetical protein | + | + | + | + | + |
| PA2823 | conserved hypothetical protein | + | + | + | + | - |
| PA2825 | OspR | - | - | - | - | + |
| PA2826 | probable glutathione peroxidase | + | + | + | + | + |
| PA2827 | conserved hypothetical protein | + | + | + | + | + |
| PA2828 | probable aminotransferase | + | + | + | + | - |
| PA2830 | heat shock protein HtpX | - | - | - | - | + |
| PA2831 | conserved hypothetical protein | + | + | + | + | + |
| PA2832 | thiopurine methyltransferase | + | + | + | + | - |
| PA2840 | probable ATP-dependent RNA helicase | + | + | + | + | + |
| PA2841 | probable enoyl-CoA hydratase/isomerase | - | + | + | + | - |
| PA2843 | probable aldolase | + | + | - | - | - |
| PA2848 | probable transcriptional regulator | - | - | - | - | + |
| PA2849 | OhrR | + | + | + | + | + |
| PA2850 | organic hydroperoxide resistance protein | + | - | - | - | + |
| PA2851 | translation elongation factor P | + | + | + | + | - |
| PA2852 | hypothetical protein | - | - | - | + | + |
| PA2853 | Outer membrane lipoprotein OprI precursor | + | + | + | + | + |
| PA2854 | conserved hypothetical protein | + | + | + | + | - |
| PA2855 | hypothetical protein | - | - | - | - | + |
| PA2856 | lysophospholipase A | + | + | + | + | - |
| PA2858 | conserved hypothetical protein | - | - | - | - | + |
| PA2870 | diguanylate cyclase | - | - | - | - | + |
| PA2871 | hypothetical protein | + | + | + | + | - |
| PA2876 | orotidine 5'-phosphate decarboxylase | + | + | + | + | - |
| PA2905 | precorrin isomerase CobH | - | - | - | + | - |
| PA2912 | probable ATP-binding component of ABC transporter | - | - | - | - | + |
| PA2926 | histidine transport protein HisP | - | - | - | - | + |
| PA2927 | hypothetical protein | + | + | + | + | - |
| PA2938 | probable transporter | - | - | - | - | + |
| PA2939 | probable aminopeptidase | + | + | + | + | - |
| PA2944 | cobalamin biosynthetic protein CobN | - | - | - | + | + |
| PA2945 | conserved hypothetical protein | + | + | + | + | - |
| PA2949 | probable lipase | - | - | - | - | + |
| PA2950 | proton motive force protein, PMF | + | + | + | + | + |
| PA2951 | electron transfer flavoprotein alpha-subunit | + | + | + | + | + |
| PA2952 | electron transfer flavoprotein beta-subunit | + | + | + | + | + |
| PA2953 | electron transfer flavoprotein-ubiquinone oxidoreductase | + | + | + | + | - |
| PA2956 | conserved hypothetical protein | - | - | - | - | + |
| PA2957 | probable transcriptional regulator | + | + | + | + | + |
| PA2958 | hypothetical protein | + | + | + | + | + |
| PA2959 | conserved hypothetical protein | + | + | + | + | - |
| PA2960 | type 4 fimbrial biogenesis protein PilZ | - | - | - | - | + |
| PA2961 | DNA polymerase III, delta prime subunit | + | + | + | + | + |
| PA2962 | thymidylate kinase | + | + | + | + | - |
| PA2964 | 4-amino-4-deoxychorismate lyase | + | + | + | + | + |
| PA2965 | beta-ketoacyl-acyl carrier protein synthase II | + | + | + | + | + |
| PA2966 | acyl carrier protein | + | + | + | + | + |
| PA2967 | 3-oxoacyl-[acyl-carrier-protein] reductase | + | + | + | + | + |
| PA2968 | malonyl-CoA-[acyl-carrier-protein] transacylase | + | + | + | + | - |
| PA2969 | fatty acid biosynthesis protein PlsX | - | - | - | - | + |
| PA2970 | 50S ribosomal protein L32 | + | + | + | + | + |
| PA2971 | conserved hypothetical protein | + | + | + | + | + |
| PA2972 | conserved hypothetical protein | + | + | + | + | + |
| PA2977 | UDP-N-acetylpyruvoylglucosamine reductase | + | + | + | + | - |
| PA2990 | probable phosphodiesterase | - | - | - | - | + |
| PA2991 | soluble pyridine nucleotide transhydrogenase | + | + | + | + | - |
| PA2998 | Na+-translocating NADH:ubiquinone oxidoreductase subunit Nrq2 | - | - | - | - | + |
| PA2999 | Na+-translocating NADH:ubiquinone oxidoreductase subunit Nrq1 | + | + | + | + | - |
| PA3000 | aromatic amino acid transport protein AroP1 | - | - | - | - | + |
| PA3001 | probable glyceraldehyde-3-phosphate dehydrogenase | + | + | + | + | + |
| PA3002 | transcription-repair coupling protein Mfd | + | + | + | + | + |
| PA3003 | hypothetical protein | + | + | + | + | + |
| PA3004 | 5-methylthioadenosine phosphorylase MtnP | + | + | + | + | + |
| PA3005 | beta-N-acetyl-D-glucosaminidase | + | + | + | + | - |
| PA3006 | transcriptional regulator PsrA | - | - | - | - | + |
| PA3007 | repressor protein LexA | + | + | + | + | - |
| PA3011 | DNA topoisomerase I | - | - | - | - | + |
| PA3012 | hypothetical protein | + | + | + | + | + |
| PA3013 | fatty-acid oxidation complex beta-subunit | + | + | + | + | + |
| PA3019 | probable ATP-binding component of ABC transporter | + | + | + | + | + |
| PA3020 | probable soluble lytic transglycosylase | + | + | + | + | + |
| PA3023 | conserved hypothetical protein | + | + | + | + | - |
| PA3027 | probable transcriptional regulator | - | - | - | - | + |
| PA3029 | molybdopterin biosynthetic protein B2 | + | + | + | + | - |
| PA3030 | molybdopterin-guanine dinucleotide biosynthesis protein MobA | - | - | - | - | + |
| PA3031 | hypothetical protein | + | + | + | + | - |
| PA3037 | hypothetical protein | - | - | - | - | + |
| PA3039 | probable transporter | - | - | - | - | + |
| PA3045 | Two-component response regulator, RocA2 | - | - | - | - | + |
| PA3046 | conserved hypothetical protein | + | + | + | + | - |
| PA3047 | probable D-alanyl-D-alanine carboxypeptidase | - | - | - | - | + |
| PA3048 | conserved hypothetical protein | + | + | + | + | + |
| PA3051 | hypothetical protein | - | - | - | - | + |
| PA3053 | probable hydrolytic enzyme | + | + | + | + | - |
| PA3054 | hypothetical protein | + | - | - | - | - |
| PA3067 | probable transcriptional regulator | - | - | - | - | + |
| PA3068 | NAD-dependent glutamate dehydrogenase | + | - | - | + | - |
| PA3080 | hypothetical protein | - | - | - | - | + |
| PA3081 | conserved hypothetical protein | + | + | + | + | - |
| PA3082 | glycine betaine transmethylase | - | - | - | - | + |
| PA3083 | aminopeptidase N | - | - | - | + | - |
| PA3086 | hypothetical protein | - | - | - | - | + |
| PA3087 | hypothetical protein | - | + | + | + | - |
| PA3091 | hypothetical protein | - | - | - | - | + |
| PA3092 | 2,4-dienoyl-CoA reductase FadH1 | + | + | + | + | - |
| PA3106 | probable short-chain dehydrogenase | - | - | - | - | + |
| PA3107 | o-succinylhomoserine sulfhydrylase | + | + | + | + | + |
| PA3108 | amidophosphoribosyltransferase | + | + | + | + | - |
| PA3110 | hypothetical protein | - | - | - | - | + |
| PA3111 | folylpolyglutamate synthetase | + | + | + | + | + |
| PA3112 | acetyl-CoA carboxylase beta subunit | + | + | + | + | - |
| PA3114 | tRNA-pseudouridine synthase I | - | - | - | - | + |
| PA3115 | Motility protein FimV | + | + | + | - | + |
| PA3116 | probable aspartate-semialdehyde dehydrogenase | + | + | + | + | + |
| PA3117 | aspartate semialdehyde dehydrogenase | + | + | + | + | + |
| PA3118 | 3-isopropylmalate dehydrogenase | + | + | + | + | - |
| PA3119 | conserved hypothetical protein | - | - | - | - | + |
| PA3120 | 3-isopropylmalate dehydratase small subunit | + | + | + | + | - |
| PA3121 | 3-isopropylmalate dehydratase large subunit | + | - | - | + | - |
| PA3122 | probable transcriptional regulator | - | - | - | - | + |
| PA3123 | conserved hypothetical protein | + | + | + | + | - |
| PA3125 | hypothetical protein | - | - | - | - | + |
| PA3126 | heat-shock protein IbpA | + | + | + | + | - |
| PA3129 | conserved hypothetical protein | - | - | - | - | + |
| PA3130 | hypothetical protein | + | + | + | + | + |
| PA3131 | probable aldolase | + | + | + | + | - |
| PA3133 | probable transcriptional regulator | - | - | - | - | + |
| PA3134 | glutamyl-tRNA synthetase | + | + | + | + | - |
| PA3138 | excinuclease ABC subunit B | + | - | - | - | + |
| PA3139 | probable amino acid aminotransferase | + | + | + | + | - |
| PA3141 | nucleotide sugar epimerase/dehydratase WbpM | + | - | - | - | - |
| PA3147 | probable glycosyl transferase WbpJ | - | - | - | - | + |
| PA3150 | LPS biosynthesis protein WbpG | - | - | - | - | + |
| PA3154 | B-band O-antigen polymerase | - | - | - | - | + |
| PA3157 | probable acetyltransferase | - | - | - | - | + |
| PA3158 | UDP-2-acetamido-2-deoxy-d-glucuronic acid 3-dehydrogenase, WbpB | - | - | - | - | + |
| PA3161 | integration host factor beta subunit | - | - | - | - | + |
| PA3162 | 30S ribosomal protein S1 | + | + | + | + | + |
| PA3163 | cytidylate kinase | + | + | + | + | + |
| PA3165 | histidinol-phosphate aminotransferase | + | + | + | + | - |
| PA3166 | chorismate mutase | + | + | + | + | + |
| PA3167 | 3-phosphoserine aminotransferase | + | + | + | + | + |
| PA3168 | DNA gyrase subunit A | + | + | + | + | + |
| PA3169 | 5-methylthioribose-1-phosphate isomerase MtnA | + | + | + | + | + |
| PA3170 | conserved hypothetical protein | + | + | + | + | + |
| PA3171 | 3-demethylubiquinone-9 3-methyltransferase | + | + | + | + | + |
| PA3172 | probable hydrolase | + | + | + | + | + |
| PA3173 | probable short-chain dehydrogenase | + | + | + | + | - |
| PA3178 | hypothetical protein | - | - | - | - | + |
| PA3179 | conserved hypothetical protein | - | - | + | + | - |
| PA3180 | hypothetical protein | - | - | - | - | + |
| PA3181 | 2-keto-3-deoxy-6-phosphogluconate aldolase | + | + | + | + | + |
| PA3182 | 6-phosphogluconolactonase | + | + | + | + | + |
| PA3185 | hypothetical protein | - | - | - | - | + |
| PA3186 | Glucose/carbohydrate outer membrane porin OprB precursor | - | + | - | - | - |
| PA3189 | probable permease of ABC sugar transporter | - | - | - | - | + |
| PA3190 | probable binding protein component of ABC sugar transporter | + | + | + | + | - |
| PA3192 | two-component response regulator GltR | - | - | - | - | + |
| PA3193 | glucokinase | + | + | + | + | + |
| PA3195 | glyceraldehyde 3-phosphate dehydrogenase | + | + | + | + | - |
| PA3198 | conserved hypothetical protein | - | - | - | - | + |
| PA3199 | conserved hypothetical protein | + | + | + | + | - |
| PA3201 | conserved hypothetical protein | - | - | - | - | + |
| PA3202 | conserved hypothetical protein | + | + | + | + | - |
| PA3207 | hypothetical protein | - | - | - | - | + |
| PA3208 | conserved hypothetical protein | + | + | + | + | - |
| PA3220 | probable transcriptional regulator | - | - | - | - | + |
| PA3221 | CsaA protein | + | + | + | + | - |
| PA3224 | hypothetical protein | - | - | - | - | + |
| PA3226 | probable hydrolase | - | - | - | - | + |
| PA3227 | peptidyl-prolyl cis-trans isomerase A | + | + | + | + | - |
| PA3239 | conserved hypothetical protein | - | - | - | - | + |
| PA3240 | conserved hypothetical protein | + | + | + | + | - |
| PA3242 | probable lauroyl acyltransferase | - | - | - | - | + |
| PA3243 | cell division inhibitor MinC | + | + | + | + | + |
| PA3244 | cell division inhibitor MinD | + | + | + | + | + |
| PA3245 | cell division topological specificity factor MinE | + | + | + | + | + |
| PA3247 | hypothetical protein | + | + | + | + | - |
| PA3249 | probable transcriptional regulator | - | - | - | - | + |
| PA3254 | probable ATP-binding component of ABC transporter | - | - | - | - | + |
| PA3255 | hypothetical protein | + | + | + | + | + |
| PA3256 | probable oxidoreductase | + | + | + | + | + |
| PA3257 | periplasmic tail-specific protease | + | + | + | + | - |
| PA3261 | hypothetical protein | - | - | - | - | + |
| PA3262 | probable peptidyl-prolyl cis-trans isomerase, FkbP-type | + | + | + | + | + |
| PA3265 | probable transporter | - | - | - | - | + |
| PA3266 | cold acclimation protein B | + | + | + | + | - |
| PA3269 | probable transcriptional regulator | - | - | - | - | + |
| PA3270 | hypothetical protein | + | + | + | + | - |
| PA3285 | probable sigma-70 factor, ECF subfamily | - | - | - | - | + |
| PA3286 | hypothetical protein | + | + | + | + | - |
| PA3294 | VgrG4a | - | - | - | - | + |
| PA3295 | probable HIT family protein | + | + | + | + | - |
| PA3298 | hypothetical protein | - | - | - | - | + |
| PA3299 | long-chain-fatty-acid--CoA ligase | + | + | + | + | - |
| PA3301 | hypothetical protein | - | - | - | - | + |
| PA3302 | conserved hypothetical protein | + | + | + | + | - |
| PA3307 | hypothetical protein | - | - | - | - | + |
| PA3308 | RNA helicase HepA | + | + | + | + | + |
| PA3309 | conserved hypothetical protein | + | + | + | + | - |
| PA3312 | probable 3-hydroxyisobutyrate dehydrogenase | - | - | - | - | + |
| PA3313 | hypothetical protein | + | + | + | + | - |
| PA3325 | conserved hypothetical protein | - | - | - | - | + |
| PA3326 | ClpP2 | + | + | + | + | + |
| PA3327 | probable non-ribosomal peptide synthetase | + | + | + | + | - |
| PA3328 | probable FAD-dependent monooxygenase | + | - | - | - | + |
| PA3330 | probable short chain dehydrogenase | + | + | + | + | + |
| PA3331 | cytochrome P450 | + | + | - | - | + |
| PA3332 | conserved hypothetical protein | + | + | + | + | + |
| PA3333 | 3-oxoacyl-[acyl-carrier-protein] synthase III | + | + | + | + | - |
| PA3336 | probable major facilitator superfamily (MFS) transporter | - | - | - | - | + |
| PA3337 | ADP-L-glycero-D-mannoheptose 6-epimerase | + | + | + | + | + |
| PA3338 | hypothetical protein | - | + | + | + | - |
| PA3340 | hypothetical protein | - | - | - | - | + |
| PA3341 | Transcriptional regulator SlyA | + | + | + | + | - |
| PA3346 | two-component response regulator | - | - | - | - | + |
| PA3347 | hypothetical protein | + | + | + | + | - |
| PA3348 | probable chemotaxis protein methyltransferase | - | - | - | - | + |
| PA3349 | probable chemotaxis protein | + | + | + | + | - |
| PA3353 | hypothetical protein | - | - | - | - | + |
| PA3354 | hypothetical protein | + | + | + | + | - |
| PA3355 | hypothetical protein | - | - | - | - | + |
| PA3356 | Glutamylpolyamine synthetase | + | + | + | + | + |
| PA3357 | D-serine dehydratase | + | + | + | + | - |
| PA3365 | probable chaperone | - | - | - | - | + |
| PA3366 | aliphatic amidase | + | + | + | + | - |
| PA3384 | ATP-binding component of ABC phosphonate transporter | - | - | - | - | + |
| PA3385 | alginate and motility regulator Z | + | + | + | + | - |
| PA3391 | regulatory protein NosR | - | - | - | - | + |
| PA3392 | nitrous-oxide reductase precursor | + | + | + | + | - |
| PA3396 | NosL protein | - | - | - | - | + |
| PA3397 | NADP+-dependent ferredoxin reductase | + | + | + | + | - |
| PA3417 | probable pyruvate dehydrogenase E1 component, alpha subunit | - | - | - | - | + |
| PA3418 | leucine dehydrogenase | + | + | + | + | - |
| PA3434 | probable transposase | - | - | - | - | + |
| PA3435 | conserved hypothetical protein | + | + | + | + | - |
| PA3438 | GTP cyclohydrolase I precursor | - | - | - | - | + |
| PA3440 | conserved hypothetical protein | + | + | + | + | + |
| PA3441 | probable molybdopterin-binding protein | + | + | + | + | - |
| PA3443 | probable permease of ABC transporter | - | - | - | - | + |
| PA3444 | conserved hypothetical protein | + | + | + | + | + |
| PA3445 | conserved hypothetical protein | + | + | + | + | - |
| PA3449 | conserved hypothetical protein | - | - | - | - | + |
| PA3450 | probable antioxidant protein | + | + | + | + | - |
| PA3452 | malate:quinone oxidoreductase | - | - | - | - | + |
| PA3453 | conserved hypothetical protein | + | + | + | + | - |
| PA3454 | probable acyl-CoA thiolase | - | - | - | - | + |
| PA3455 | conserved hypothetical protein | + | + | + | + | - |
| PA3457 | hypothetical protein | - | - | - | - | + |
| PA3458 | probable transcriptional regulator | + | + | + | + | + |
| PA3459 | probable glutamine amidotransferase | + | + | + | + | - |
| PA3460 | probable acetyltransferase | - | - | - | - | + |
| PA3461 | conserved hypothetical protein | + | + | + | + | - |
| PA3465 | conserved hypothetical protein | - | - | - | - | + |
| PA3470 | hypothetical protein | - | - | - | - | + |
| PA3471 | probable malic enzyme | + | + | + | + | - |
| PA3476 | autoinducer synthesis protein RhlI | - | - | - | - | + |
| PA3477 | transcriptional regulator RhlR | + | + | + | + | + |
| PA3478 | rhamnosyltransferase chain B | + | + | + | + | + |
| PA3479 | rhamnosyltransferase chain A | + | + | + | + | + |
| PA3480 | probable deoxycytidine triphosphate deaminase | + | + | + | + | + |
| PA3481 | conserved hypothetical protein | + | + | + | + | + |
| PA3482 | methionyl-tRNA synthetase | + | + | + | + | - |
| PA3495 | endonuclease III | - | - | - | - | + |
| PA3523 | probable Resistance-Nodulation-Cell Division (RND) efflux membrane fusion protein precursor | - | - | - | - | + |
| PA3524 | lactoylglutathione lyase | + | + | + | + | + |
| PA3525 | argininosuccinate synthase | + | + | + | + | - |
| PA3526 | probable outer membrane protein precursor | - | - | - | - | + |
| PA3527 | dihydroorotase | - | - | - | + | - |
| PA3528 | ribonuclease T | - | - | - | - | + |
| PA3530 | bacterioferritin-associated ferredoxin Bfd | - | - | - | - | + |
| PA3531 | bacterioferritin | + | + | + | + | - |
| PA3532 | hypothetical protein | - | - | - | - | + |
| PA3533 | conserved hypothetical protein | + | + | + | + | - |
| PA3535 | probable serine protease | - | + | - | + | - |
| PA3538 | probable ATP-binding component of ABC transporter | - | - | - | - | + |
| PA3539 | conserved hypothetical protein | + | + | + | + | + |
| PA3540 | GDP-mannose 6-dehydrogenase AlgD | + | + | + | + | - |
| PA3542 | alginate biosynthesis protein Alg44 | + | - | - | - | + |
| PA3543 | alginate biosynthetic protein AlgK precursor | + | + | + | + | - |
| PA3544 | Alginate production outer membrane protein AlgE precursor | - | - | - | - | + |
| PA3545 | alginate-c5-mannuronan-epimerase AlgG | + | + | + | + | + |
| PA3546 | alginate biosynthesis protein AlgX | + | + | + | + | - |
| PA3547 | poly(beta-d-mannuronate) lyase precursor AlgL | + | - | - | + | - |
| PA3549 | alginate o-acetyltransferase AlgJ | - | - | - | - | + |
| PA3551 | phosphomannose isomerase / guanosine 5'-diphospho-D-mannose pyrophosphorylase | + | + | + | + | + |
| PA3552 | ArnB | - | + | + | + | - |
| PA3561 | 1-phosphofructokinase | - | - | - | - | + |
| PA3565 | probable transcriptional regulator | - | - | - | - | + |
| PA3566 | conserved hypothetical protein | + | + | + | + | + |
| PA3567 | probable oxidoreductase | + | + | + | + | + |
| PA3569 | 3-hydroxyisobutyrate dehydrogenase | + | + | + | + | + |
| PA3570 | methylmalonate-semialdehyde dehydrogenase | + | + | + | + | - |
| PA3574 | NalD | - | - | + | - | - |
| PA3578 | conserved hypothetical protein | - | - | - | - | + |
| PA3579 | probable carbohydrate kinase | + | + | + | + | + |
| PA3580 | conserved hypothetical protein | + | + | + | + | - |
| PA3581 | glycerol uptake facilitator protein | - | - | - | - | + |
| PA3582 | glycerol kinase | + | + | + | + | - |
| PA3583 | glycerol-3-phosphate regulon repressor | - | - | - | - | + |
| PA3584 | glycerol-3-phosphate dehydrogenase | - | + | + | + | - |
| PA3603 | diacylglycerol kinase | - | - | - | - | + |
| PA3604 | response regulator ErdR | + | + | + | + | - |
| PA3610 | polyamine transport protein PotD | - | - | - | - | + |
| PA3611 | hypothetical protein | + | + | + | + | - |
| PA3612 | conserved hypothetical protein | - | - | - | - | + |
| PA3613 | hypothetical protein | + | + | + | + | + |
| PA3615 | hypothetical protein | + | + | + | + | - |
| PA3616 | conserved hypothetical protein | - | - | - | - | + |
| PA3617 | RecA protein | + | + | + | + | + |
| PA3618 | conserved hypothetical protein | + | + | + | + | - |
| PA3619 | hypothetical protein | - | - | - | - | + |
| PA3620 | DNA mismatch repair protein MutS | + | + | + | + | + |
| PA3621 | ferredoxin I | + | + | + | + | + |
| PA3622 | sigma factor RpoS | - | + | - | - | - |
| PA3624 | L-isoaspartate protein carboxylmethyltransferase type II | + | - | - | - | + |
| PA3625 | survival protein SurE | + | + | + | + | + |
| PA3626 | conserved hypothetical protein | + | + | + | + | + |
| PA3627 | 2C-methyl-D-erythritol 2,4-cyclodiphosphate synthase | + | + | + | + | - |
| PA3628 | probable esterase | - | - | - | - | + |
| PA3629 | alcohol dehydrogenase class III | + | + | + | + | - |
| PA3631 | conserved hypothetical protein | - | - | - | - | + |
| PA3632 | conserved hypothetical protein | - | + | - | + | - |
| PA3634 | conserved hypothetical protein | - | - | - | - | + |
| PA3635 | enolase |  | + | | + | + |
| PA3636 | 2-dehydro-3-deoxyphosphooctonate aldolase | + | + | + | + | + |
| PA3637 | CTP synthase | - | + | + | + | - |
| PA3638 | conserved hypothetical protein | - | - | - | - | + |
| PA3639 | acetyl-coenzyme A carboxylase carboxyl transferase (alpha subunit) | + | + | + | + | + |
| PA3640 | DNA polymerase III, alpha chain | + | + | + | + | - |
| PA3642 | ribonuclease HII | - | - | - | - | + |
| PA3643 | lipid A-disaccharide synthase | + | + | + | + | + |
| PA3644 | UDP-N-acetylglucosamine acyltransferase | + | + | + | + | + |
| PA3645 | (3R)-hydroxymyristoyl-[acyl carrier protein] dehydratase | + | + | + | + | - |
| PA3646 | UDP-3-O-[3-hydroxylauroyl] glucosamine N-acyltransferase | + | - | - | - | + |
| PA3647 | probable outer membrane protein precursor | + | + | + | + | - |
| PA3650 | 1-deoxy-d-xylulose 5-phosphate reductoisomerase | + | - | - | - | - |
| PA3652 | undecaprenyl pyrophosphate synthetase | - | - | - | - | + |
| PA3653 | ribosome recycling factor | + | + | + | + | + |
| PA3654 | uridylate kinase | + | + | + | + | + |
| PA3655 | elongation factor Ts | + | + | + | + | + |
| PA3656 | 30S ribosomal protein S2 | + | + | + | + | + |
| PA3657 | methionine aminopeptidase | + | + | + | + | - |
| PA3658 | protein-PII uridylyltransferase | - | - | - | - | + |
| PA3659 | probable aminotransferase | + | + | + | + | - |
| PA3663 | hypothetical protein | - | - | - | - | + |
| PA3664 | conserved hypothetical protein | + | + | + | + | - |
| PA3665 | hypothetical protein | - | - | - | - | + |
| PA3666 | tetrahydrodipicolinate succinylase | + | + | + | + | + |
| PA3667 | probable pyridoxal-phosphate dependent enzyme | + | + | + | + | - |
| PA3673 | glycerol-3-phosphate acyltransferase | - | - | - | - | + |
| PA3674 | hypothetical protein | + | + | + | + | - |
| PA3675 | hypothetical protein | + | - | - | - | - |
| PA3677 | MexJ | - | - | - | - | + |
| PA3678 | MexL | + | + | + | + | - |
| PA3679 | hypothetical protein | - | - | - | - | + |
| PA3680 | conserved hypothetical protein | + | + | + | + | - |
| PA3682 | hypothetical protein | + | - | - | + | - |
| PA3684 | hypothetical protein | - | - | - | - | + |
| PA3685 | conserved hypothetical protein | + | + | + | + | + |
| PA3686 | adenylate kinase | + | + | + | + | - |
| PA3689 | probable transcriptional regulator | + | + | + | + | + |
| PA3690 | probable metal-transporting P-type ATPase | - | + | + | + | - |
| PA3691 | hypothetical protein | - | - | - | - | + |
| PA3692 | Lipotoxon F, LptF | + | + | + | + | - |
| PA3697 | hypothetical protein | - | - | - | - | + |
| PA3698 | hypothetical protein | + | + | + | + | + |
| PA3700 | lysyl-tRNA synthetase | + | + | + | + | + |
| PA3701 |  | + | + | + | + | - |
| PA3711 | probable transcriptional regulator | - | - | - | - | + |
| PA3712 | hypothetical protein | + | + | + | + | + |
| PA3713 | spermidine dehydrogenase, SpdH | + | + | + | + | - |
| PA3714 | probable two-component response regulator | - | - | - | - | + |
| PA3715 | hypothetical protein | + | + | + | + | - |
| PA3722 | hypothetical protein | - | - | - | - | + |
| PA3723 | probable FMN oxidoreductase | + | + | + | + | + |
| PA3724 | elastase LasB | + | + | + | + | - |
| PA3725 | single-stranded-DNA-specific exonuclease RecJ | - | - | - | - | + |
| PA3726 | conserved hypothetical protein | + | + | + | + | + |
| PA3727 | hypothetical protein | + | + | + | + | - |
| PA3730 | hypothetical protein | - | - | - | - | + |
| PA3731 | conserved hypothetical protein | + | + | + | + | + |
| PA3733 | hypothetical protein | + | + | - | - | - |
| PA3734 | hypothetical protein | - | - | - | - | + |
| PA3735 | threonine synthase | + | + | + | + | + |
| PA3736 | homoserine dehydrogenase | + | + | + | + | + |
| PA3737 | thiol:disulfide interchange protein DsbC | + | + | + | + | - |
| PA3741 | hypothetical protein | - | - | - | - | + |
| PA3742 | 50S ribosomal protein L19 | + | + | + | + | - |
| PA3744 | 16S rRNA processing protein | - | - | - | - | + |
| PA3745 | 30S ribosomal protein S16 | + | + | + | + | - |
| PA3750 | hypothetical protein | - | - | - | - | + |
| PA3751 | phosphoribosylglycinamide formyltransferase 2 | + | + | + | + | - |
| PA3752 | hypothetical protein | - | - | - | - | + |
| PA3753 | conserved hypothetical protein | + | + | + | + | - |
| PA3762 | hypothetical protein | + | - | - | - | + |
| PA3763 | phosphoribosylformylglycinamidine synthase | + | + | + | + | - |
| PA3768 | probable metallo-oxidoreductase | - | - | - | - | + |
| PA3769 | GMP synthase | + | + | + | + | + |
| PA3778 | probable transcriptional regulator | - | - | - | - | + |
| PA3779 | hypothetical protein | + | + | + | + | - |
| PA3784 | hypothetical protein | - | - | - | - | + |
| PA3785 | conserved hypothetical protein | + | + | + | + | - |
| PA3786 | hypothetical protein | - | - | - | - | + |
| PA3787 | conserved hypothetical protein | + | + | + | + | - |
| PA3789 | hypothetical protein | - | - | - | - | + |
| PA3791 | hypothetical protein | - | - | - | - | + |
| PA3792 | 2-isopropylmalate synthase | + | + | + | + | + |
| PA3793 | hypothetical protein | + | + | + | + | - |
| PA3794 | hypothetical protein | - | - | - | - | + |
| PA3795 | probable oxidoreductase | + | + | + | + | - |
| PA3796 | hypothetical protein | - | - | - | - | + |
| PA3797 | conserved hypothetical protein | + | + | + | + | + |
| PA3798 | probable aminotransferase | + | + | + | + | + |
| PA3799 | conserved hypothetical protein | + | + | + | + | + |
| PA3800 | conserved hypothetical protein | + | + | + | + | - |
| PA3801 | conserved hypothetical protein | - | - | - | - | + |
| PA3802 | histidyl-tRNA synthetase | + | + | + | + | - |
| PA3805 | type 4 fimbrial biogenesis protein PilF | - | - | - | - | + |
| PA3806 | conserved hypothetical protein | + | + | + | + | + |
| PA3807 | nucleoside diphosphate kinase | + | + | + | + | - |
| PA3808 | conserved hypothetical protein | - | - | - | - | + |
| PA3809 | ferredoxin [2Fe-2S] | + | + | + | + | + |
| PA3810 | heat shock protein HscA | + | + | + | + | - |
| PA3811 | heat shock protein HscB | - | - | - | - | + |
| PA3812 | probable iron-binding protein IscA | + | + | + | + | - |
| PA3813 | probable iron-binding protein IscU | - | - | - | - | + |
| PA3814 | L-cysteine desulfurase (pyridoxal phosphate-dependent) | + | + | + | + | - |
| PA3816 | O-acetylserine synthase | - | - | - | - | + |
| PA3817 | probable methyltransferase | + | + | + | + | + |
| PA3818 | extragenic suppressor protein SuhB | + | + | + | + | + |
| PA3819 | conserved hypothetical protein | + | + | + | + | - |
| PA3821 | secretion protein SecD | - | - | - | - | + |
| PA3822 | conserved hypothetical protein | + | + | + | + | + |
| PA3823 | queuine tRNA-ribosyltransferase | + | + | + | + | + |
| PA3824 | S-adenosylmethionine:trna ribosyltransferase-isomerase | + | - | - | - | - |
| PA3830 | probable transcriptional regulator | - | - | - | - | + |
| PA3831 | leucine aminopeptidase | + | + | + | + | - |
| PA3833 | hypothetical protein | - | - | - | - | + |
| PA3834 | valyl-tRNA synthetase | + | + | + | + | - |
| PA3835 | hypothetical protein | - | - | - | - | + |
| PA3836 | hypothetical protein | + | + | + | + | - |
| PA3843 | hypothetical protein | - | - | - | - | + |
| PA3845 | probable transcriptional regulator | - | - | - | - | + |
| PA3846 | hypothetical protein | + | + | + | + | - |
| PA3848 | hypothetical protein | - | - | - | - | + |
| PA3849 | conserved hypothetical protein | + | + | + | + | - |
| PA3857 | phosphatidylcholine synthase | - | - | - | - | + |
| PA3858 | probable amino acid-binding protein | + | + | + | + | + |
| PA3859 | carboxylesterase | + | + | + | + | - |
| PA3861 | ATP-dependent RNA helicase RhlB | - | - | - | - | + |
| PA3862 | NAD(P)H-dependent anabolic L-arginine dehydrogenase, DauB | + | + | + | + | - |
| PA3864 | Transcriptional regulator of the dauBAR operon, DauR | - | - | - | - | + |
| PA3865 | probable amino acid binding protein | + | + | + | + | - |
| PA3873 | respiratory nitrate reductase delta chain | - | - | - | - | + |
| PA3875 | respiratory nitrate reductase alpha chain | + | + | + | + | - |
| PA3881 | hypothetical protein | - | - | - | - | + |
| PA3882 | hypothetical protein | + | + | + | + | - |
| PA3885 | protein tyrosine phosphatase TpbA | - | - | - | - | + |
| PA3886 | hypothetical protein | + | + | + | + | - |
| PA3888 | OpuC ABC transporter, permease protein, OpuCD | - | - | - | - | + |
| PA3889 | OpuC ABC transporter, periplasmic substrate-binding protein, OpuCC | + | + | + | + | - |
| PA3901 | Fe(III) dicitrate transport protein FecA | - | - | - | - | + |
| PA3902 | hypothetical protein | + | + | + | + | + |
| PA3903 | peptide chain release factor 3 | + | + | + | + | - |
| PA3907 | hypothetical protein | - | - | - | - | + |
| PA3908 | hypothetical protein | + | + | + | + | - |
| PA3918 | molybdopterin biosynthetic protein C | - | - | - | - | + |
| PA3919 | conserved hypothetical protein | + | + | + | + | - |
| PA3921 | probable transcriptional regulator | - | - | - | - | + |
| PA3922 | conserved hypothetical protein | + | + | + | + | - |
| PA3924 | probable medium-chain acyl-CoA ligase | - | - | - | - | + |
| PA3925 | probable acyl-CoA thiolase | + | + | + | + | - |
| PA3930 | cyanide insensitive terminal oxidase | - | - | - | - | + |
| PA3931 | conserved hypothetical protein | + | + | + | + | - |
| PA3937 | probable ATP-binding component of ABC taurine transporter | - | - | - | - | + |
| PA3938 | probable periplasmic taurine-binding protein precursor | + | + | + | + | - |
| PA3939 | hypothetical protein | - | - | - | - | + |
| PA3940 | probable DNA binding protein | + | + | + | + | - |
| PA3941 | hypothetical protein | - | - | - | - | + |
| PA3942 | acyl-CoA thioesterase II | + | + | + | + | - |
| PA3944 | conserved hypothetical protein | + | + | + | + | + |
| PA3949 | hypothetical protein | - | - | - | - | + |
| PA3950 | probable ATP-dependent RNA helicase | + | + | + | + | - |
| PA3952 | hypothetical protein | + | + | + | + | - |
| PA3955 | hypothetical protein | - | - | - | - | + |
| PA3956 | hypothetical protein | + | + | + | + | - |
| PA3964 | hypothetical protein | - | - | - | - | + |
| PA3966 | hypothetical protein | - | - | - | - | + |
| PA3967 | hypothetical protein | + | + | + | + | - |
| PA3969 | conserved hypothetical protein | - | - | - | - | + |
| PA3970 | AMP nucleosidase | + | + | + | + | - |
| PA3971 | hypothetical protein | - | - | - | - | + |
| PA3972 | probable acyl-CoA dehydrogenase | + | + | + | + | - |
| PA3974 | Lost Adherence Sensor, LadS | - | - | - | - | + |
| PA3975 | phosphomethylpyrimidine kinase | + | + | + | + | + |
| PA3976 | thiamin-phosphate pyrophosphorylase | + | + | + | + | + |
| PA3977 | glutamate-1-semialdehyde 2,1-aminomutase | + | + | + | + | - |
| PA3979 | hypothetical protein | - | - | - | - | + |
| PA3980 | conserved hypothetical protein | + | + | + | + | + |
| PA3981 | conserved hypothetical protein | - | + | + | + | - |
| PA3982 | conserved hypothetical protein | - | - | - | - | + |
| PA3983 | conserved hypothetical protein | + | + | + | + | - |
| PA3986 | hypothetical protein | - | - | - | - | + |
| PA3991 | hypothetical protein | - | - | - | - | + |
| PA3992 | hypothetical protein | + | + | + | + | - |
| PA3995 | probable transcriptional regulator | - | - | - | - | + |
| PA3996 | lipoate synthase | + | + | + | + | - |
| PA3997 | lipoate-protein ligase B | - | - | - | - | + |
| PA3998 | conserved hypothetical protein | + | + | + | + | + |
| PA3999 | D-ala-D-ala-carboxypeptidase | + | + | + | + | - |
| PA4000 | RlpA | - | - | - | - | + |
| PA4004 | conserved hypothetical protein | - | + | - | - | - |
| PA4005 | conserved hypothetical protein | - | - | - | + | - |
| PA4006 | nicotinic acid mononucleotide adenylyltransferase | - | - | - | - | + |
| PA4007 | gamma-glutamyl phosphate reductase | + | + | + | + | - |
| PA4014 | hypothetical protein | - | - | - | - | + |
| PA4015 | conserved hypothetical protein | + | + | + | + | - |
| PA4016 | hypothetical protein | - | - | - | - | + |
| PA4017 | conserved hypothetical protein | + | + | + | + | - |
| PA4019 | probable aromatic acid decarboxylase | + | - | - | - | + |
| PA4020 | UDP-N-acetylmuramate:L-alanyl-gamma-D-glutamyl-meso-diaminopimelate ligase | + | + | + | + | - |
| PA4021 | probable transcriptional regulator | - | - | - | - | + |
| PA4025 | probable ethanolamine ammonia-lyase light chain | - | - | - | - | + |
| PA4026 | probable acetyltransferase | + | + | + | + | - |
| PA4030 | conserved hypothetical protein | - | - | - | - | + |
| PA4031 | inorganic pyrophosphatase | + | + | + | + | - |
| PA4034 | aquaporin Z | - | - | - | - | + |
| PA4035 | hypothetical protein | - | + | - | - | - |
| PA4042 | exodeoxyribonuclease VII small subunit | - | - | - | - | + |
| PA4046 | hypothetical protein | - | - | - | - | + |
| PA4047 | GTP cyclohydrolase II | + | + | + | + | - |
| PA4051 | thiamine monophosphate kinase | - | - | - | - | + |
| PA4052 | NusB protein | + | + | + | + | + |
| PA4053 | 6,7-dimethyl-8-ribityllumazine synthase | + | + | + | + | + |
| PA4054 | GTP cyclohydrolase II / 3,4-dihydroxy-2-butanone 4-phosphate synthase | + | + | + | + | + |
| PA4055 | riboflavin synthase alpha chain | + | + | + | + | + |
| PA4056 | riboflavin-specific deaminase/reductase | - | - | - | - | + |
| PA4057 | NrdR | + | + | + | + | - |
| PA4058 | hypothetical protein | - | - | - | - | + |
| PA4060 | hypothetical protein | - | - | - | - | + |
| PA4061 | probable thioredoxin | + | + | + | + | - |
| PA4062 | hypothetical protein | - | - | - | - | + |
| PA4063 | hypothetical protein | + | + | + | - | - |
| PA4066 | hypothetical protein | + | + | + | + | + |
| PA4067 | Outer membrane protein OprG precursor | + | + | + | + | + |
| PA4068 | probable epimerase | + | + | + | + | + |
| PA4069 | hypothetical protein | + | + | + | + | - |
| PA4077 | probable transcriptional regulator | - | - | - | - | + |
| PA4078 | probable nonribosomal peptide synthetase | + | + | + | + | + |
| PA4079 | probable dehydrogenase | - | + | + | + | - |
| PA4109 | transcriptional regulator AmpR | - | - | - | - | + |
| PA4110 | beta-lactamase precursor | + | + | + | + | - |
| PA4114 | lysine decarboxylase | - | - | - | - | + |
| PA4115 | conserved hypothetical protein | + | + | + | + | - |
| PA4118 | hypothetical protein | - | - | - | - | + |
| PA4119 | aminoglycoside 3'-phosphotransferase type IIb | + | + | + | + | - |
| PA4128 | conserved hypothetical protein | - | - | - | - | + |
| PA4129 | hypothetical protein | + | + | + | + | + |
| PA4130 | probable sulfite or nitrite reductase | + | + | + | + | + |
| PA4131 | probable iron-sulfur protein | + | + | + | + | - |
| PA4134 | hypothetical protein | - | - | - | - | + |
| PA4135 | probable transcriptional regulator | + | + | + | + | - |
| PA4137 | probable porin | + | + | + | + | + |
| PA4138 | tyrosyl-tRNA synthetase | + | + | + | + | - |
| PA4149 | conserved hypothetical protein | - | - | - | - | + |
| PA4150 | probable dehydrogenase E1 component | + | + | + | + | + |
| PA4151 | acetoin catabolism protein AcoB | + | + | + | + | + |
| PA4152 | probable hydrolase | + | + | + | + | - |
| PA4162 | probable short-chain dehydrogenase | - | - | - | - | + |
| PA4163 | hypothetical protein | + | + | + | + | - |
| PA4174 | probable transcriptional regulator | - | - | - | - | + |
| PA4175 | protease IV | + | + | + | + | + |
| PA4176 | peptidyl-prolyl cis-trans isomerase C2 | + | + | + | + | - |
| PA4179 | probable porin | - | - | - | - | + |
| PA4180 | probable acetolactate synthase large subunit | + | + | + | + | - |
| PA4189 | probable aldehyde dehydrogenase | - | - | - | - | + |
| PA4190 | probable FAD-dependent monooxygenase | + | + | + | + | - |
| PA4194 | probable permease of ABC transporter | - | - | - | - | + |
| PA4195 | probable binding protein component of ABC transporter | + | + | + | + | - |
| PA4198 | probable AMP-binding enzyme | - | - | - | - | + |
| PA4199 | probable acyl-CoA dehydrogenase | + | + | + | + | + |
| PA4200 | hypothetical protein | + | + | + | + | + |
| PA4201 | D-alanine-D-alanine ligase A | + | + | + | + | + |
| PA4202 | nitronate monooxygenase | + | + | + | + | - |
| PA4203 | probable transcriptional regulator | - | - | - | - | + |
| PA4204 | periplasmic gluconolactonase, PpgL | + | + | + | + | - |
| PA4205 | hypothetical protein | - | - | - | - | + |
| PA4206 | probable Resistance-Nodulation-Cell Division (RND) efflux membrane fusion protein precursor | + | + | + | + | - |
| PA4208 | probable outer membrane protein precursor | - | - | - | - | + |
| PA4210 | probable phenazine biosynthesis protein | - | - | - | - | + |
| PA4211 | probable phenazine biosynthesis protein | + | + | + | - | + |
| PA4212 | phenazine biosynthesis protein PhzC | + | + | + | + | + |
| PA4213 | phenazine biosynthesis protein PhzD | + | + | + | + | - |
| PA4214 | phenazine biosynthesis protein PhzE | - | - | - | - | + |
| PA4216 | probable pyridoxamine 5'-phosphate oxidase | + | + | + | + | + |
| PA4217 | flavin-containing monooxygenase | + | + | + | + | - |
| PA4223 | probable ATP-binding component of ABC transporter | - | - | - | - | + |
| PA4224 | pyochelin biosynthetic protein PchG | + | + | + | + | + |
| PA4225 | pyochelin synthetase | + | + | + | + | + |
| PA4226 | dihydroaeruginoic acid synthetase | - | - | - | + | - |
| PA4227 | transcriptional regulator PchR | - | - | - | - | + |
| PA4228 | pyochelin biosynthesis protein PchD | + | + | + | + | - |
| PA4229 | pyochelin biosynthetic protein PchC | - | - | - | - | + |
| PA4230 | salicylate biosynthesis protein PchB | + | + | + | + | + |
| PA4232 | single-stranded DNA-binding protein | + | + | + | + | - |
| PA4233 | probable major facilitator superfamily (MFS) transporter | - | - | - | - | + |
| PA4234 | excinuclease ABC subunit A | + | + | + | + | + |
| PA4235 | bacterial ferritin | + | + | + | + | + |
| PA4236 | catalase | + | + | + | + | + |
| PA4237 | 50S ribosomal protein L17 | + | + | + | + | + |
| PA4238 | DNA-directed RNA polymerase alpha chain | + | + | + | + | + |
| PA4239 | 30S ribosomal protein S4 | + | + | + | + | + |
| PA4240 | 30S ribosomal protein S11 | + | + | + | + | + |
| PA4241 | 30S ribosomal protein S13 | + | + | + | + | + |
| PA4242 | 50S ribosomal protein L36 | - | - | - | + | - |
| PA4243 | secretion protein SecY | - | - | - | - | + |
| PA4244 | 50S ribosomal protein L15 | + | + | + | + | + |
| PA4245 | 50S ribosomal protein L30 | + | + | + | + | + |
| PA4246 | 30S ribosomal protein S5 | + | + | + | + | + |
| PA4247 | 50S ribosomal protein L18 | + | + | + | + | + |
| PA4248 | 50S ribosomal protein L6 | + | + | + | + | + |
| PA4249 | 30S ribosomal protein S8 | + | + | + | + | + |
| PA4250 | 30S ribosomal protein S14 | + | + | + | + | + |
| PA4251 | 50S ribosomal protein L5 | + | + | + | + | + |
| PA4252 | 50S ribosomal protein L24 | + | + | + | + | + |
| PA4253 | 50S ribosomal protein L14 | + | + | + | + | + |
| PA4254 | 30S ribosomal protein S17 | + | + | + | + | + |
| PA4255 | 50S ribosomal protein L29 | + | + | + | + | + |
| PA4256 | 50S ribosomal protein L16 | + | + | + | + | + |
| PA4257 | 30S ribosomal protein S3 | + | + | + | + | + |
| PA4258 | 50S ribosomal protein L22 | + | + | + | + | + |
| PA4259 | 30S ribosomal protein S19 | + | + | + | + | + |
| PA4260 | 50S ribosomal protein L2 | + | + | + | + | - |
| PA4261 | 50S ribosomal protein L23 | - | - | - | - | + |
| PA4262 | 50S ribosomal protein L4 | + | + | + | + | + |
| PA4263 | 50S ribosomal protein L3 | + | + | + | + | + |
| PA4264 | 30S ribosomal protein S10 | + | + | + | + | - |
| PA4265 | elongation factor Tu | - | - | - | - | + |
| PA4266 | elongation factor G | + | + | + | + | + |
| PA4267 | 30S ribosomal protein S7 | + | + | + | + | + |
| PA4268 | 30S ribosomal protein S12 | + | + | + | + | + |
| PA4269 | DNA-directed RNA polymerase beta* chain | + | + | + | + | + |
| PA4270 | DNA-directed RNA polymerase beta chain | + | + | + | + | + |
| PA4271 | 50S ribosomal protein L7 / L12 | + | + | + | + | + |
| PA4272 | 50S ribosomal protein L10 | + | + | + | + | + |
| PA4273 | 50S ribosomal protein L1 | + | + | + | + | + |
| PA4274 | 50S ribosomal protein L11 | + | + | + | + | + |
| PA4275 | transcription antitermination protein NusG | + | + | + | + | - |
| PA4276 | secretion protein SecE | - | - | - | - | + |
| PA4278 | hypothetical protein | - | - | - | - | + |
| PA4279 | hypothetical protein | - | + | + | + | - |
| PA4284 | exodeoxyribonuclease V beta chain | - | - | - | - | + |
| PA4285 | exodeoxyribonuclease V gamma chain | - | - | + | + | - |
| PA4286 | hypothetical protein | + | - | - | - | - |
| PA4296 | two-component response regulator, PprB | - | - | + | - | - |
| PA4307 | chemotactic transducer PctC | - | - | - | - | + |
| PA4308 | conserved hypothetical protein | + | + | + | + | - |
| PA4309 | chemotactic transducer PctA | - | - | - | + | + |
| PA4313 | hypothetical protein | - | - | - | - | + |
| PA4314 | formyltetrahydrofolate deformylase | + | + | + | + | + |
| PA4315 | transcriptional regulator MvaT, P16 subunit | + | + | + | + | + |
| PA4316 | exodeoxyribonuclease I | + | + | + | + | - |
| PA4321 | hypothetical protein | - | - | - | - | + |
| PA4327 | hypothetical protein | - | - | - | - | + |
| PA4328 | hypothetical protein | + | + | + | + | - |
| PA4329 | pyruvate kinase II | - | + | - | - | - |
| PA4332 | SadC | - | - | - | - | + |
| PA4333 | probable fumarase | + | + | + | + | - |
| PA4335 | hypothetical protein | - | - | - | - | + |
| PA4336 | conserved hypothetical protein | + | + | + | + | - |
| PA4339 | probable phospholipase | - | - | - | - | + |
| PA4340 | hypothetical protein | + | + | + | + | - |
| PA4344 | probable hydrolase | - | - | - | - | + |
| PA4345 | hypothetical protein | + | + | + | + | - |
| PA4347 | hypothetical protein | - | - | - | - | + |
| PA4348 | conserved hypothetical protein | + | + | + | + | - |
| PA4351 | OlsA | - | - | - | - | + |
| PA4352 | conserved hypothetical protein | + | + | + | + | - |
| PA4355 | PyeM | - | - | - | - | + |
| PA4356 | xenobiotic reductase | + | + | + | + | - |
| PA4361 | probable oxidoreductase | - | - | - | - | + |
| PA4362 | hypothetical protein | + | + | + | + | - |
| PA4365 | probable transporter | - | - | - | - | + |
| PA4366 | superoxide dismutase | + | + | + | + | - |
| PA4369 | hypothetical protein | + | + | + | + | + |
| PA4370 | Insulin-cleaving metalloproteinase outer membrane protein precursor | + | + | + | + | - |
| PA4371 | hypothetical protein | - | - | - | - | + |
| PA4372 | hypothetical protein | + | + | + | + | - |
| PA4373 | hypothetical protein | + | + | - | - | - |
| PA4375 | Resistance-Nodulation-Cell Division (RND) multidrug efflux transporter MexW | - | - | - | - | + |
| PA4376 | nicotinate phosphoribosyltransferase | + | + | + | + | - |
| PA4381 | probable two-component response regulator | + | - | + | - | - |
| PA4384 | hypothetical protein | - | - | - | - | + |
| PA4385 | GroEL protein | + | + | + | + | - |
| PA4387 | conserved hypothetical protein | - | - | - | - | + |
| PA4389 | probable short-chain dehydrogenase | + | + | + | + | - |
| PA4394 | conserved hypothetical protein | - | - | - | - | + |
| PA4399 | conserved hypothetical protein | - | - | - | - | + |
| PA4400 | probable pyrophosphohydrolase | + | + | + | + | + |
| PA4401 | probable glutathione S-transferase | + | + | + | + | + |
| PA4402 | glutamate N-acetyltransferase | + | + | + | + | + |
| PA4403 | secretion protein SecA | + | + | + | + | - |
| PA4405 | hypothetical protein | - | - | - | - | + |
| PA4406 | UDP-3-O-acyl-N-acetylglucosamine deacetylase | + | + | + | + | + |
| PA4407 | cell division protein FtsZ | + | + | + | + | + |
| PA4408 | cell division protein FtsA | + | + | + | + | - |
| PA4409 | cell division protein FtsQ | - | - | - | - | + |
| PA4410 | D-alanine--D-alanine ligase | + | + | + | + | + |
| PA4411 | UDP-N-acetylmuramate--alanine ligase | + | + | + | + | - |
| PA4412 | UDP-N-acetylglucosamine--N-acetylmuramyl-(pentapeptide) pyrophosphoryl-undecaprenol N-acetylglucosamine transferase | + | - | - | - | - |
| PA4413 | cell division protein FtsW | - | - | - | - | + |
| PA4414 | UDP-N-acetylmuramoylalanine--D-glutamate ligase | + | + | + | + | - |
| PA4415 | phospho-N-acetylmuramoyl-pentapeptide-transferase | - | - | - | - | + |
| PA4416 | UDP-N-acetylmuramoylalanyl-D-glutamyl-2, 6-diaminopimelate--D-alanyl-D-alanyl ligase | + | + | + | + | + |
| PA4417 | UDP-N-acetylmuramoylalanyl-D-glutamate-2, 6-diaminopimelate ligase | + | + | + | + | - |
| PA4419 | cell division protein FtsL | - | - | - | - | + |
| PA4420 | conserved hypothetical protein | + | + | + | + | - |
| PA4422 | conserved hypothetical protein | - | - | - | - | + |
| PA4424 | conserved hypothetical protein | - | - | - | - | + |
| PA4425 | sedoheptulose 7-phosphate isomerase GmhA | + | + | + | + | - |
| PA4427 | stringent starvation protein B | - | - | - | - | + |
| PA4428 | stringent starvation protein A | + | + | + | + | + |
| PA4429 | probable cytochrome c1 precursor | + | + | + | + | + |
| PA4430 | probable cytochrome b | + | + | + | + | + |
| PA4431 | probable iron-sulfur protein | + | + | + | + | + |
| PA4432 | 30S ribosomal protein S9 | + | + | + | + | - |
| PA4434 | probable oxidoreductase | + | + | + | + | - |
| PA4437 | hypothetical protein | - | - | - | - | + |
| PA4438 | conserved hypothetical protein | + | + | + | + | - |
| PA4439 | tryptophanyl-tRNA synthetase | - | - | - | - | + |
| PA4441 | hypothetical protein | + | + | + | + | + |
| PA4442 | ATP sulfurylase GTP-binding subunit/APS kinase | + | + | + | + | + |
| PA4443 | ATP sulfurylase small subunit | + | + | + | + | - |
| PA4444 | soluble and membrane-bound lytic transglycosylases | + | + | + | + | + |
| PA4445 | conserved hypothetical protein | + | + | + | + | - |
| PA4446 | AlgW protein | - | - | - | - | + |
| PA4447 | histidinol-phosphate aminotransferase | + | + | + | + | + |
| PA4448 | histidinol dehydrogenase | + | + | + | + | + |
| PA4449 | ATP-phosphoribosyltransferase | + | + | + | + | + |
| PA4450 | UDP-N-acetylglucosamine 1-carboxyvinyltransferase | + | + | + | + | + |
| PA4451 | conserved hypothetical protein | + | + | + | + | - |
| PA4452 | conserved hypothetical protein | - | - | - | - | + |
| PA4453 | conserved hypothetical protein | + | + | + | + | + |
| PA4454 | conserved hypothetical protein | + | + | + | + | - |
| PA4456 | probable ATP-binding component of ABC transporter | - | - | - | - | + |
| PA4457 | arabinose-5-phosphate isomerase KdsD | + | + | + | + | + |
| PA4458 | conserved hypothetical protein | + | + | + | + | - |
| PA4459 | conserved hypothetical protein | - | - | - | - | + |
| PA4460 | conserved hypothetical protein | + | + | + | + | + |
| PA4461 | probable ATP-binding component of ABC transporter | + | + | + | + | + |
| PA4463 | conserved hypothetical protein | + | + | + | + | + |
| PA4464 | nitrogen regulatory IIA protein | + | + | + | + | - |
| PA4471 | hypothetical protein | - | - | - | - | + |
| PA4472 | PmbA protein | + | + | + | + | - |
| PA4473 | hypothetical protein | - | - | - | - | + |
| PA4474 | conserved hypothetical protein | + | + | + | + | + |
| PA4475 | conserved hypothetical protein | + | + | + | - | - |
| PA4476 | hypothetical protein | - | - | - | - | + |
| PA4477 | cytoplasmic axial filament protein | + | + | + | + | - |
| PA4480 | rod shape-determining protein MreC | - | - | - | - | + |
| PA4481 | rod shape-determining protein MreB | + | + | + | + | - |
| PA4482 | Glu-tRNA(Gln) amidotransferase subunit C | - | - | - | - | + |
| PA4483 | Glu-tRNA(Gln) amidotransferase subunit A | + | + | + | + | + |
| PA4484 | Glu-tRNA(Gln) amidotransferase subunit B | + | + | + | + | - |
| PA4489 | MagD | + | - | - | - | - |
| PA4492 | MagA | - | - | - | - | + |
| PA4493 | RoxR | + | + | + | + | - |
| PA4494 | RoxS | - | - | - | - | + |
| PA4495 | hypothetical protein | + | + | + | + | + |
| PA4497 | probable binding protein component of ABC transporter | + | + | + | + | + |
| PA4498 | probable metallopeptidase | + | + | + | + | - |
| PA4499 | probable transcriptional regulator | - | - | - | - | + |
| PA4500 | probable binding protein component of ABC transporter | + | + | + | + | + |
| PA4501 | Glycine-glutamate dipeptide porin OpdP | - | - | + | - | + |
| PA4502 | probable binding protein component of ABC transporter | + | + | + | + | - |
| PA4505 | probable ATP-binding component of ABC transporter | + | + | + | + | + |
| PA4518 | hypothetical protein | - | - | - | - | + |
| PA4519 | ornithine decarboxylase | + | + | + | + | - |
| PA4523 | hypothetical protein | - | - | - | - | + |
| PA4524 | nicotinate-nucleotide pyrophosphorylase | + | + | + | + | + |
| PA4525 | type 4 fimbrial precursor PilA | + | - | + | - | - |
| PA4528 | type 4 prepilin peptidase PilD | - | - | - | - | + |
| PA4529 | dephosphocoenzyme A kinase | + | + | + | + | - |
| PA4532 | hypothetical protein | - | - | - | - | + |
| PA4533 | hypothetical protein | + | + | + | + | - |
| PA4541 | hypothetical protein | - | - | - | - | + |
| PA4542 | ClpB protein | + | + | + | + | - |
| PA4543 | conserved hypothetical protein | - | - | - | - | + |
| PA4544 | pseudouridine synthase | - | - | + | - | - |
| PA4546 | two-component sensor PilS | - | - | - | - | + |
| PA4547 | two-component response regulator PilR | + | + | + | + | + |
| PA4548 | probable D-amino acid oxidase | + | + | + | + | - |
| PA4553 | type 4 fimbrial biogenesis protein PilX | - | - | - | - | + |
| PA4554 | type 4 fimbrial biogenesis protein PilY1 | + | + | + | + | - |
| PA4556 | type 4 fimbrial biogenesis protein PilE | - | - | - | - | + |
| PA4557 | LytB protein | + | + | + | + | + |
| PA4558 | probable peptidyl-prolyl cis-trans isomerase, FkbP-type | + | + | + | + | - |
| PA4559 | prolipoprotein signal peptidase | - | - | - | - | + |
| PA4560 | isoleucyl-tRNA synthetase | + | + | + | + | + |
| PA4561 | riboflavin kinase/FAD synthase | + | + | + | + | - |
| PA4562 | conserved hypothetical protein | - | - | - | - | + |
| PA4563 | 30S ribosomal protein S20 | + | + | + | + | - |
| PA4564 | conserved hypothetical protein | - | - | - | - | + |
| PA4565 | glutamate 5-kinase | + | + | + | + | + |
| PA4566 | GTP-binding protein Obg | + | + | + | + | + |
| PA4567 | 50S ribosomal protein L27 | + | + | + | + | + |
| PA4568 | 50S ribosomal protein L21 | + | + | + | + | + |
| PA4569 | octaprenyl-diphosphate synthase | + | + | + | + | - |
| PA4572 | peptidyl-prolyl cis-trans isomerase FklB | + | - | - | + | - |
| PA4575 | hypothetical protein | - | - | - | - | + |
| PA4576 | probable ATP-dependent protease | + | + | + | + | - |
| PA4577 | hypothetical protein | + | - | - | + | - |
| PA4581 | transcriptional regulator RtcR | - | - | - | - | + |
| PA4582 | conserved hypothetical protein | + | + | + | + | + |
| PA4583 | conserved hypothetical protein | + | + | + | + | - |
| PA4584 | conserved hypothetical protein | - | - | - | - | + |
| PA4585 | RNA 3'-terminal phosphate cyclase | + | + | + | + | - |
| PA4586 | hypothetical protein | - | - | - | - | + |
| PA4587 | cytochrome c551 peroxidase precursor | + | + | + | + | - |
| PA4589 | probable outer membrane protein precursor | - | - | - | - | + |
| PA4590 | protein activator | - | + | - | + | - |
| PA4594 | probable ATP-binding component of ABC transporter | + | + | + | - | + |
| PA4595 | probable ATP-binding component of ABC transporter | + | - | - | - | - |
| PA4601 | motility regulator | - | - | - | - | + |
| PA4603 | hypothetical protein | - | - | - | - | + |
| PA4604 | conserved hypothetical protein | + | + | + | + | - |
| PA4609 | DNA repair protein RadA | - | + | - | - | - |
| PA4610 | hypothetical protein | - | - | - | - | + |
| PA4611 | hypothetical protein | + | + | + | + | - |
| PA4613 | catalase | - | - | - | - | + |
| PA4614 | conductance mechanosensitive channel | + | + | + | + | + |
| PA4615 | probable oxidoreductase | + | + | + | + | - |
| PA4620 | hypothetical protein | - | - | - | - | + |
| PA4621 | probable oxidoreductase | - | - | + | - | - |
| PA4624 | cyclic diguanylate-regulated TPS partner B, CdrB | + | + | + | + | + |
| PA4631 | hypothetical protein | - | - | - | - | + |
| PA4632 | hypothetical protein | + | + | + | + | - |
| PA4635 | conserved hypothetical protein | - | - | - | - | + |
| PA4636 | hypothetical protein | + | + | + | + | - |
| PA4638 | hypothetical protein | - | - | - | - | + |
| PA4639 | hypothetical protein | + | + | + | + | - |
| PA4640 | malate:quinone oxidoreductase | - | - | - | - | + |
| PA4642 | hypothetical protein | - | + | + | + | - |
| PA4643 | hypothetical protein | - | - | - | - | + |
| PA4644 | hypothetical protein | + | + | + | + | + |
| PA4645 | probable purine/pyrimidine phosphoribosyl transferase | + | + | + | + | + |
| PA4646 | uracil phosphoribosyltransferase | + | + | + | + | - |
| PA4647 | uracil permease | - | - | - | - | + |
| PA4648 | Pilin subunit CupE1 | + | + | + | + | - |
| PA4655 | ferrochelatase | - | - | - | - | + |
| PA4657 | hypothetical protein | + | + | + | + | - |
| PA4660 | deoxyribodipyrimidine photolyase | - | - | - | - | + |
| PA4661 | Lipid A 3-O-deacylase | + | + | + | + | - |
| PA4662 | glutamate racemase | - | - | - | - | + |
| PA4663 | molybdopterin biosynthesis MoeB protein | + | + | + | + | - |
| PA4664 | probable methyl transferase | + | + | + | + | + |
| PA4665 | peptide chain release factor 1 | + | + | + | + | - |
| PA4669 | isopentenyl monophosphate kinase | - | - | - | - | + |
| PA4670 | ribose-phosphate pyrophosphokinase | + | + | + | + | + |
| PA4671 | probable ribosomal protein L25 | + | + | + | + | + |
| PA4672 | peptidyl-tRNA hydrolase | + | + | + | + | + |
| PA4673 | conserved hypothetical protein | + | + | + | + | - |
| PA4674 | conserved hypothetical protein | - | - | - | - | + |
| PA4675 | ChtA | + | + | + | + | + |
| PA4676 | probable carbonic anhydrase | + | + | + | + | - |
| PA4683 | hypothetical protein | - | - | - | - | + |
| PA4684 | hypothetical protein | - | + | + | + | - |
| PA4685 | hypothetical protein | - | - | - | - | + |
| PA4686 | hypothetical protein | + | + | + | + | + |
| PA4687 | ferric iron-binding periplasmic protein HitA | + | + | + | + | - |
| PA4692 | conserved hypothetical protein | + | + | + | + | - |
| PA4693 | phosphatidylserine synthase | - | - | - | - | + |
| PA4694 | ketol-acid reductoisomerase | + | + | + | + | + |
| PA4695 | acetolactate synthase isozyme III small subunit | + | + | + | + | + |
| PA4696 | acetolactate synthase large subunit | + | + | + | + | - |
| PA4707 | PhuU | - | - | - | - | + |
| PA4708 | Heme-transport protein, PhuT | + | + | + | + | - |
| PA4715 | probable aminotransferase | + | - | - | - | - |
| PA4719 | probable transporter | - | - | - | - | + |
| PA4720 | tRNA (uracil-5-)-methyltransferase | + | + | + | + | + |
| PA4722 | probable aminotransferase | + | + | + | + | + |
| PA4723 | suppressor protein DksA | + | + | + | + | - |
| PA4725 | two-component sensor CbrA | - | - | - | - | + |
| PA4726 | two-component response regulator CbrB | + | + | + | + | + |
| PA4728 | 2-amino-4-hydroxy-6-hydroxymethyldihydropteridine pyrophosphokinase | - | - | - | - | + |
| PA4729 | 3-methyl-2-oxobutanoate hydroxymethyltransferase | + | + | + | + | + |
| PA4730 | pantoate--beta-alanine ligase | + | + | + | + | - |
| PA4731 | aspartate 1-decarboxylase precursor | - | - | - | - | + |
| PA4732 | glucose-6-phosphate isomerase | + | + | + | + | + |
| PA4738 | conserved hypothetical protein | + | + | + | + | + |
| PA4739 | conserved hypothetical protein | + | + | + | + | + |
| PA4740 | polyribonucleotide nucleotidyltransferase | + | + | + | + | - |
| PA4741 | 30S ribosomal protein S15 | - | - | - | - | + |
| PA4742 | tRNA pseudouridine 55 synthase | + | + | + | + | + |
| PA4743 | ribosome-binding factor A | + | + | + | + | + |
| PA4744 | translation initiation factor IF-2 | + | + | + | + | + |
| PA4745 | N utilization substance protein A | + | + | + | + | - |
| PA4747 | secretion protein SecG | - | - | - | - | + |
| PA4748 | triosephosphate isomerase | + | + | + | + | + |
| PA4749 | phosphoglucosamine mutase | + | + | + | + | + |
| PA4750 | dihydropteroate synthase | + | + | + | + | + |
| PA4751 | cell division protein FtsH | + | + | + | + | - |
| PA4752 | cell division protein FtsJ | - | - | - | - | + |
| PA4753 | conserved hypothetical protein | + | + | + | + | - |
| PA4754 | hypothetical protein | - | - | - | - | + |
| PA4755 | transcription elongation factor GreA | + | + | + | + | + |
| PA4756 | carbamoylphosphate synthetase large subunit | + | + | + | + | - |
| PA4757 | conserved hypothetical protein | - | - | - | - | + |
| PA4758 | carbamoyl-phosphate synthase small chain | + | + | + | + | + |
| PA4759 | dihydrodipicolinate reductase | + | + | + | + | + |
| PA4760 | DnaJ protein | + | + | + | + | + |
| PA4761 | DnaK protein | + | + | + | + | + |
| PA4762 | heat shock protein GrpE | + | + | + | + | - |
| PA4763 | DNA repair protein RecN | - | - | - | - | + |
| PA4764 | ferric uptake regulation protein | + | + | + | + | - |
| PA4766 | conserved hypothetical protein | - | - | - | - | + |
| PA4767 | conserved hypothetical protein | + | + | + | + | + |
| PA4768 | SmpB protein | + | + | + | + | - |
| PA4770 | L-lactate permease | - | - | - | - | + |
| PA4771 | L-lactate dehydrogenase | + | + | + | + | - |
| PA4775 | hypothetical protein | - | - | - | - | + |
| PA4776 | PmrA: two-component regulator system response regulator PmrA | + | + | + | + | - |
| PA4777 | PmrB: two-component regulator system signal sensor kinase PmrB | - | - | - | - | + |
| PA4785 | probable acyl-CoA thiolase | - | - | - | - | + |
| PA4793 | hypothetical protein | + | - | - | - | - |
| PA4808 | L-seryl-tRNA(ser) selenium transferase | - | - | - | - | + |
| PA4840 | conserved hypothetical protein | - | - | - | - | + |
| PA4841 | conserved hypothetical protein | + | + | + | + | + |
| PA4842 | hypothetical protein | - | + | + | + | - |
| PA4845 | thiol:disulfide interchange protein DipZ | + | + | + | + | + |
| PA4846 | 3-dehydroquinate dehydratase | + | + | + | + | + |
| PA4847 | biotin carboxyl carrier protein (BCCP) | + | + | + | + | + |
| PA4848 | biotin carboxylase | + | + | + | + | - |
| PA4849 | hypothetical protein | - | - | - | - | + |
| PA4850 | ribosomal protein L11 methyltransferase | + | + | + | + | - |
| PA4853 | DNA-binding protein Fis | - | - | - | - | + |
| PA4854 | phosphoribosylaminoimidazolecarboxamide formyltransferase | + | + | + | + | + |
| PA4855 | phosphoribosylamine--glycine ligase | + | + | + | + | - |
| PA4871 | hypothetical protein | - | - | - | - | + |
| PA4872 | hypothetical protein | + | + | + | + | - |
| PA4875 | hypothetical protein | - | - | - | - | + |
| PA4876 | osmotically inducible lipoprotein OsmE | + | + | + | + | - |
| PA4879 | conserved hypothetical protein | - | - | - | - | + |
| PA4880 | probable bacterioferritin | + | + | + | + | - |
| PA4899 | probable aldehyde dehydrogenase | + | + | + | + | - |
| PA4906 | probable transcriptional regulator | - | - | - | - | + |
| PA4907 | probable short-chain dehydrogenase | + | + | + | + | - |
| PA4912 | branched chain amino acid ABC transporter membrane protein | - | - | - | - | + |
| PA4913 | probable binding protein component of ABC transporter | + | + | + | + | - |
| PA4915 | probable chemotaxis transducer | - | - | - | - | + |
| PA4916 | hypothetical protein | + | + | + | + | - |
| PA4918 | hypothetical protein | - | - | - | - | + |
| PA4919 | nicotinate phosphoribosyltransferase | + | + | + | + | + |
| PA4920 | NH3-dependent NAD synthetase | + | + | + | + | - |
| PA4921 | cholinesterase, ChoE | - | - | - | - | + |
| PA4922 | azurin precursor | + | + | + | + | - |
| PA4923 | conserved hypothetical protein | - | - | - | + | - |
| PA4929 | hypothetical protein | - | - | - | - | + |
| PA4930 | biosynthetic alanine racemase | + | + | + | + | - |
| PA4931 | replicative DNA helicase | - | - | - | - | + |
| PA4932 | 50S ribosomal protein L9 | + | + | + | + | - |
| PA4933 | hypothetical protein | - | - | - | - | + |
| PA4934 | 30S ribosomal protein S18 | + | + | + | + | + |
| PA4935 | 30S ribosomal protein S6 | + | + | + | + | + |
| PA4937 | exoribonuclease RNase R | + | + | + | + | + |
| PA4938 | adenylosuccinate synthetase | + | + | + | + | + |
| PA4939 | conserved hypothetical protein | + | + | + | + | - |
| PA4940 | conserved hypothetical protein | - | - | - | - | + |
| PA4941 | protease subunit HflC | + | + | + | + | + |
| PA4942 | protease subunit HflK | + | + | + | + | + |
| PA4944 | Hfq | + | + | + | + | - |
| PA4947 | N-acetylmuramoyl-L-alanine amidase | + | + | + | + | - |
| PA4948 | conserved hypothetical protein | - | - | - | - | + |
| PA4949 | conserved hypothetical protein | - | + | - | + | - |
| PA4951 | oligoribonuclease | - | + | - | + | - |
| PA4955 | hypothetical protein | - | - | - | - | + |
| PA4956 | thiosulfate:cyanide sulfurtransferase | + | + | + | + | - |
| PA4957 | phosphatidylserine decarboxylase | - | - | - | - | + |
| PA4958 | hypothetical protein | - | + | - | + | - |
| PA4959 | FimX | + | - | - | - | + |
| PA4960 | probable phosphoserine phosphatase | + | + | + | + | - |
| PA4963 | hypothetical protein | - | - | - | - | + |
| PA4965 | hypothetical protein | - | - | - | - | + |
| PA4966 | hypothetical protein | + | + | + | + | + |
| PA4967 | topoisomerase IV subunit B | + | + | + | + | - |
| PA4968 | conserved hypothetical protein | - | - | - | - | + |
| PA4969 | Cyclic AMP (cAMP) Phosphodiesterase, CpdA | + | + | + | + | - |
| PA4970 | conserved hypothetical protein | - | - | - | - | + |
| PA4971 | adenosine diphosphate sugar pyrophosphatase | + | + | + | + | - |
| PA4973 | thiamin biosynthesis protein ThiC | - | - | - | - | + |
| PA4975 | NAD(P)H quinone oxidoreductase | - | - | - | - | + |
| PA4976 | Arginine:Pyruvate Transaminas, AruH | - | + | + | + | - |
| PA4990 | SMR multidrug efflux transporter | - | - | - | - | + |
| PA4991 | hypothetical protein | + | + | + | + | + |
| PA4992 | hypothetical protein | + | + | + | + | - |
| PA4995 | probable acyl-CoA dehydrogenase | - | - | - | - | + |
| PA4997 | transport protein MsbA | - | - | - | - | + |
| PA4998 | conserved hypothetical protein | - | + | + | + | - |
| PA5005 | probable carbamoyl transferase | + | + | + | + | - |
| PA5009 | lipopolysaccharide kinase WaaP | - | - | - | - | + |
| PA5010 | UDP-glucose:(heptosyl) LPS alpha 1,3-glucosyltransferase WaaG | + | + | + | + | - |
| PA5012 | heptosyltransferase II | - | - | - | - | + |
| PA5013 | branched-chain amino acid transferase | + | + | + | + | - |
| PA5014 | glutamate-ammonia-ligase adenylyltransferase | - | - | - | - | + |
| PA5015 | pyruvate dehydrogenase | + | + | + | + | + |
| PA5016 | dihydrolipoamide acetyltransferase | + | + | + | + | - |
| PA5017 | DipA | - | - | - | - | + |
| PA5018 | peptide methionine sulfoxide reductase | + | + | + | + | - |
| PA5024 | conserved hypothetical protein | - | - | - | - | + |
| PA5025 | homocysteine synthase | + | + | + | + | - |
| PA5026 | hypothetical protein | - | - | - | - | + |
| PA5027 | hypothetical protein | + | + | + | + | - |
| PA5035 | glutamate synthase small chain | + | + | + | + | - |
| PA5037 | hypothetical protein | - | - | - | + | + |
| PA5038 | 3-dehydroquinate synthase | + | + | + | + | + |
| PA5039 | shikimate kinase | + | + | + | + | + |
| PA5040 | Type 4 fimbrial biogenesis outer membrane protein PilQ precursor | + | + | + | + | - |
| PA5045 | penicillin-binding protein 1A | - | - | - | - | + |
| PA5046 | malic enzyme | + | + | + | + | - |
| PA5048 | probable nuclease | - | - | - | - | + |
| PA5049 | 50S ribosomal protein L31 | + | + | + | + | - |
| PA5051 | arginyl-tRNA synthetase | + | + | + | + | - |
| PA5053 | heat shock protein HslV | - | - | - | - | + |
| PA5055 | hypothetical protein | + | + | + | + | - |
| PA5059 | probable transcriptional regulator | - | - | - | - | + |
| PA5060 | polyhydroxyalkanoate synthesis protein PhaF | - | - | + | - | - |
| PA5062 | conserved hypothetical protein | - | - | - | - | + |
| PA5063 | ubiquinone biosynthesis methyltransferase UbiE | + | + | + | + | - |
| PA5064 | hypothetical protein | - | - | - | - | + |
| PA5066 | phosphoribosyl-AMP cyclohydrolase | + | + | + | + | - |
| PA5075 | probable permease of ABC transporter | - | - | - | - | + |
| PA5076 | probable binding protein component of ABC transporter | + | + | + | + | - |
| PA5077 | OpgH |  | - | | + | + |
| PA5078 | OpgG |  | + | | + | + |
| PA5079 | conserved hypothetical protein | + | + | + | + | + |
| PA5080 | prolyl aminopeptidase | + | + | + | + | - |
| PA5081 | hypothetical protein | - | - | - | - | + |
| PA5082 | DguC | + | + | + | + | + |
| PA5083 | DguB | + | + | + | + | - |
| PA5090 | VgrG5 | - | - | - | - | + |
| PA5091 | N-formylglutamate amidohydrolase | + | + | + | + | - |
| PA5092 | imidazolone-5-propionate hydrolase HutI | + | - | - | - | - |
| PA5097 | probable amino acid permease | - | - | - | - | + |
| PA5098 | histidine ammonia-lyase | + | + | + | + | - |
| PA5099 | probable transporter | - | - | - | - | + |
| PA5100 | urocanase | + | + | + | + | - |
| PA5102 | hypothetical protein | - | - | - | - | + |
| PA5103 | PuuR | + | + | + | - | - |
| PA5104 | conserved hypothetical protein | - | - | - | - | + |
| PA5105 | histidine utilization repressor HutC | + | + | + | + | - |
| PA5108 | hypothetical protein | - | - | - | - | + |
| PA5109 | hypothetical protein | + | + | + | + | + |
| PA5110 | fructose-1,6-bisphosphatase | + | + | + | + | + |
| PA5111 | lactoylglutathione lyase | + | + | + | + | + |
| PA5112 | esterase EstA | + | + | + | + | - |
| PA5114 | hypothetical protein | - | - | - | - | + |
| PA5115 | conserved hypothetical protein | + | + | + | + | - |
| PA5116 | probable transcriptional regulator | - | - | - | - | + |
| PA5117 | regulatory protein TypA | + | + | + | + | - |
| PA5118 | thiazole biosynthesis protein ThiI | + | + | + | - | + |
| PA5119 | glutamine synthetase | + | + | + | + | - |
| PA5122 | hypothetical protein | + | - | - | - | - |
| PA5124 | two-component sensor NtrB | - | - | - | - | + |
| PA5125 | two-component response regulator NtrC | + | + | + | + | - |
| PA5127 | probable rRNA methylase | - | - | - | - | + |
| PA5128 | secretion protein SecB | + | + | + | + | - |
| PA5130 | conserved hypothetical protein | - | - | - | - | + |
| PA5131 | phosphoglycerate mutase | + | + | + | + | - |
| PA5133 | conserved hypothetical protein | - | - | - | - | + |
| PA5134 | carboxyl-terminal processing protease, CtpA | + | + | + | + | - |
| PA5136 | hypothetical protein | - | - | - | - | + |
| PA5137 | hypothetical protein | + | + | + | + | + |
| PA5138 | hypothetical protein | + | + | + | + | - |
| PA5139 | hypothetical protein | + | - | - | - | + |
| PA5140 | imidazoleglycerol-phosphate synthase, cyclase subunit | + | + | + | + | + |
| PA5141 | phosphoribosylformimino-5-aminoimidazole carboxamide | + | + | + | + | + |
| PA5142 | glutamine amidotransferase | + | + | + | + | + |
| PA5143 | imidazoleglycerol-phosphate dehydratase | + | + | + | + | - |
| PA5145 | hypothetical protein | - | - | - | - | + |
| PA5146 | hypothetical protein | - | + | + | + | - |
| PA5152 | probable ATP-binding component of ABC transporter | - | - | - | - | + |
| PA5153 | amino acid (lysine/arginine/ornithine/histidine/octopine) ABC transporter periplasmic binding protein | + | + | + | + | - |
| PA5160 | drug efflux transporter | - | - | - | - | + |
| PA5161 | dTDP-D-glucose 4,6-dehydratase | + | + | + | + | + |
| PA5162 | dTDP-4-dehydrorhamnose reductase | + | + | + | + | + |
| PA5163 | glucose-1-phosphate thymidylyltransferase | + | + | + | + | + |
| PA5164 | dTDP-4-dehydrorhamnose 3,5-epimerase | + | + | + | + | - |
| PA5165 | DctB | - | - | - | - | + |
| PA5167 | DctP | + | + | + | + | - |
| PA5170 | arginine/ornithine antiporter | - | - | - | - | + |
| PA5171 | arginine deiminase | + | + | + | + | + |
| PA5172 | ornithine carbamoyltransferase, catabolic | + | + | + | + | + |
| PA5173 | carbamate kinase | + | + | + | + | + |
| PA5174 | probable beta-ketoacyl synthase | + | + | + | + | + |
| PA5175 | 3,5-bisphosphate nucleotidase CysQ | + | + | + | + | + |
| PA5176 | conserved hypothetical protein | + | + | + | + | - |
| PA5177 | probable hydrolase | - | - | - | - | + |
| PA5178 | conserved hypothetical protein | + | + | + | + | - |
| PA5183 | hypothetical protein | - | - | - | - | + |
| PA5184 | hypothetical protein | + | + | + | + | - |
| PA5189 | probable transcriptional regulator | - | - | - | - | + |
| PA5190 | NAD(P)H quinone oxidoreductase | + | + | + | + | - |
| PA5191 | hypothetical protein | - | - | - | - | + |
| PA5192 | phosphoenolpyruvate carboxykinase | + | + | + | + | + |
| PA5193 | heat shock protein HSP33 | + | + | + | + | - |
| PA5195 | probable heat shock protein | - | - | - | - | + |
| PA5196 | hypothetical protein | + | + | + | + | - |
| PA5197 | ribosomal protein S6 modification protein | - | - | - | - | + |
| PA5198 | LD-carboxypeptidase | + | + | + | + | - |
| PA5199 | AmgS | - | - | - | - | + |
| PA5200 | AmgR | + | + | + | + | + |
| PA5201 | conserved hypothetical protein | - | + | + | + | - |
| PA5202 | hypothetical protein | + | - | - | - | + |
| PA5205 | conserved hypothetical protein | - | - | - | - | + |
| PA5206 | acetylornithine deacetylase | + | + | + | + | - |
| PA5208 | conserved hypothetical protein | - | - | - | - | + |
| PA5209 | hypothetical protein | + | + | + | + | + |
| PA5210 | probable secretion pathway ATPase | + | + | + | + | - |
| PA5212 | hypothetical protein | - | - | - | - | + |
| PA5213 | glycine cleavage system protein P1 | + | + | + | - | + |
| PA5214 | glycine cleavage system protein H1 | + | + | + | + | + |
| PA5215 | glycine-cleavage system protein T1 | + | + | + | + | - |
| PA5216 | probable permease of ABC iron transporter | - | - | - | - | + |
| PA5217 | probable binding protein component of ABC iron transporter | + | + | + | + | - |
| PA5219 | hypothetical protein | - | - | - | - | + |
| PA5220 | hypothetical protein | + | + | + | + | - |
| PA5222 | hypothetical protein | - | - | - | - | + |
| PA5224 | aminopeptidase P | - | - | - | - | + |
| PA5228 | conserved hypothetical protein | - | - | - | - | + |
| PA5229 | conserved hypothetical protein | + | + | + | + | - |
| PA5231 | probable ATP-binding/permease fusion ABC transporter | - | - | - | - | + |
| PA5232 | conserved hypothetical protein | + | + | + | + | - |
| PA5236 | probable aromatic hydrocarbon reductase | - | - | - | - | + |
| PA5237 | conserved hypothetical protein | + | + | + | + | - |
| PA5238 | probable O-antigen acetylase | - | - | - | - | + |
| PA5239 | transcription termination factor Rho | + | + | + | + | + |
| PA5240 | thioredoxin | + | + | + | + | + |
| PA5242 | polyphosphate kinase | + | + | + | + | + |
| PA5243 | delta-aminolevulinic acid dehydratase | + | + | + | + | - |
| PA5244 | conserved hypothetical protein | - | - | - | - | + |
| PA5245 | conserved hypothetical protein | + | + | + | + | - |
| PA5252 | probable ATP-binding component of ABC transporter | + | - | - | - | + |
| PA5253 | alginate regulatory protein AlgP | - | + | - | + | - |
| PA5256 | disulfide bond formation protein | - | - | - | - | + |
| PA5257 | hypothetical protein | + | + | + | + | + |
| PA5258 | hypothetical protein | + | + | + | + | - |
| PA5259 | uroporphyrinogen-III synthetase | - | - | - | - | + |
| PA5260 | porphobilinogen deaminase | + | + | + | + | + |
| PA5261 | alginate biosynthesis regulatory protein AlgR | + | + | + | + | - |
| PA5262 | FimS | - | - | - | - | + |
| PA5263 | argininosuccinate lyase | + | + | + | + | - |
| PA5266 | VgrG6 | - | - | - | - | + |
| PA5270 | hypothetical protein | + | - | - | - | - |
| PA5273 | hypothetical protein | - | - | - | - | + |
| PA5276 | Lipopeptide LppL precursor | - | - | - | - | + |
| PA5277 | diaminopimelate decarboxylase | + | + | + | + | + |
| PA5278 | diaminopimelate epimerase | + | + | + | + | - |
| PA5280 | site-specific recombinase Sss | - | - | - | - | + |
| PA5281 | probable hydrolase | + | + | + | + | - |
| PA5284 | hypothetical protein | - | - | - | - | + |
| PA5285 | hypothetical protein | + | + | + | + | + |
| PA5286 | conserved hypothetical protein | + | + | + | + | - |
| PA5287 | ammonium transporter AmtB | - | - | - | - | + |
| PA5288 | nitrogen regulatory protein P-II 2 | + | + | + | + | - |
| PA5297 | pyruvate dehydrogenase (cytochrome) | - | - | - | - | + |
| PA5298 | xanthine phosphoribosyltransferase | + | + | + | + | - |
| PA5299 | hypothetical protein | - | - | - | - | + |
| PA5302 | catabolic alanine racemase | + | + | + | + | + |
| PA5303 | conserved hypothetical protein | + | + | + | + | + |
| PA5304 | D-amino acid dehydrogenase, small subunit | + | + | + | + | - |
| PA5307 | hypothetical protein | - | - | - | - | + |
| PA5308 | leucine-responsive regulatory protein | + | + | + | + | + |
| PA5309 | FAD-dependent oxidoreductase | + | + | + | + | - |
| PA5311 | probable major facilitator superfamily (MFS) transporter | - | - | - | - | + |
| PA5312 | Aldehyde dehydrogenase | + | + | + | + | + |
| PA5313 | Transaminase | + | + | + | + | + |
| PA5314 | hypothetical protein | + | + | + | + | + |
| PA5315 | 50S ribosomal protein L33 | + | + | + | + | + |
| PA5316 | 50S ribosomal protein L28 | + | + | + | + | + |
| PA5317 | probable binding protein component of ABC dipeptide transporter | + | + | + | + | - |
| PA5319 | DNA repair protein RadC | - | - | - | - | + |
| PA5320 | Phosphopantothenoylcysteine synthase/(R)-4'-phospho-N-pantothenoylcysteine decarboxylase | + | + | + | + | - |
| PA5321 | deoxyuridine 5'-triphosphate nucleotidohydrolase | - | - | - | - | + |
| PA5322 | phosphomannomutase AlgC | + | + | + | + | + |
| PA5323 | acetylglutamate kinase | + | + | + | + | - |
| PA5328 | SphB | - | - | - | - | + |
| PA5330 | hypothetical protein | + | + | + | + | + |
| PA5331 | orotate phosphoribosyltransferase | + | + | + | + | + |
| PA5332 | catabolite repression control protein | + | + | + | + | - |
| PA5333 | conserved hypothetical protein | - | - | - | - | + |
| PA5334 | ribonuclease PH | + | + | + | + | - |
| PA5337 | RNA polymerase omega subunit | + | - | - | - | - |
| PA5338 | guanosine-3',5'-bis(diphosphate) 3'-pyrophosphohydrolase | + | - | - | - | + |
| PA5339 | conserved hypothetical protein | + | + | + | + | - |
| PA5342 | probable transcriptional regulator | - | - | - | - | + |
| PA5343 | hypothetical protein | + | + | + | + | + |
| PA5344 | OxyR | + | + | + | + | - |
| PA5345 | ATP-dependent DNA helicase RecG | - | - | - | - | + |
| PA5346 | SadB | + | + | + | + | - |
| PA5347 | hypothetical protein | - | - | - | - | + |
| PA5348 | probable DNA-binding protein | + | + | + | + | + |
| PA5349 | rubredoxin reductase | - | + | - | + | - |
| PA5356 | transcriptional regulator GlcC | - | - | - | - | + |
| PA5357 | hypothetical protein | + | + | + | + | - |
| PA5358 | 4-hydroxybenzoate-octaprenyl transferase | - | - | - | - | + |
| PA5359 | hypothetical protein | + | + | + | + | - |
| PA5363 | hypothetical protein | - | - | - | - | + |
| PA5364 | probable two-component response regulator | - | + | + | + | - |
| PA5365 | phosphate uptake regulatory protein PhoU | - | - | - | - | + |
| PA5366 | ATP-binding component of ABC phosphate transporter | + | - | - | + | - |
| PA5368 | membrane protein component of ABC phosphate transporter | - | - | - | - | + |
| PA5369 | phosphate ABC transporter, periplasmic phosphate-binding protein, PstS | + | + | + | + | - |
| PA5370 | probable major facilitator superfamily (MFS) transporter | - | - | - | - | + |
| PA5371 | conserved hypothetical protein | + | + | + | + | - |
| PA5372 | choline dehydrogenase | - | - | - | - | + |
| PA5373 | betaine aldehyde dehydrogenase | + | + | + | + | - |
| PA5377 | CbcW | - | - | - | - | + |
| PA5378 | CbcX | + | + | + | + | - |
| PA5412 | hypothetical protein | - | - | - | - | + |
| PA5413 | low specificity l-threonine aldolase | + | + | + | + | - |
| PA5421 | glutathione-independent formaldehyde dehydrogenase | - | - | - | - | + |
| PA5422 | hypothetical protein | + | + | + | + | + |
| PA5424 | conserved hypothetical protein | - | - | - | - | + |
| PA5425 | phosphoribosylaminoimidazole carboxylase | + | + | + | + | + |
| PA5426 | phosphoribosylaminoimidazole carboxylase, catalytic subunit | + | + | + | + | + |
| PA5427 | alcohol dehydrogenase | + | + | + | + | - |
| PA5429 | aspartate ammonia-lyase | + | + | - | - | - |
| PA5434 | tryptophan permease | - | - | - | - | + |
| PA5435 | probable transcarboxylase subunit | + | + | + | + | + |
| PA5436 | probable biotin carboxylase subunit of a transcarboxylase | + | + | + | + | - |
| PA5437 | probable transcriptional regulator | - | - | - | - | + |
| PA5438 | probable transcriptional regulator | + | + | + | + | - |
| PA5439 | probable glucose-6-phosphate dehydrogenase | + | + | + | + | + |
| PA5441 | hypothetical protein | + | + | + | + | - |
| PA5442 | conserved hypothetical protein | - | - | - | - | + |
| PA5443 | DNA helicase II | + | + | + | + | - |
| PA5444 | conserved hypothetical protein | - | - | - | - | + |
| PA5445 | probable coenzyme A transferase | + | + | + | + | - |
| PA5474 | probable metalloprotease | - | - | - | - | + |
| PA5475 | hypothetical protein | + | + | + | + | - |
| PA5482 | hypothetical protein | - | - | - | - | + |
| PA5483 | two-component response regulator AlgB | + | + | + | + | - |
| PA5488 | hypothetical protein | - | - | - | - | + |
| PA5489 | thiol:disulfide interchange protein DsbA | + | + | + | + | + |
| PA5491 | probable cytochrome | + | + | + | + | + |
| PA5493 | DNA polymerase I | + | + | + | + | - |
| PA5494 | hypothetical protein | - | - | - | - | + |
| PA5495 | homoserine kinase | + | + | + | + | + |
| PA5496 | class II (cobalamin-dependent) ribonucleotide-diphosphate reductase subunit, NrdJb | + | + | + | + | + |
| PA5497 | class II (cobalamin-dependent) ribonucleotide-diphosphate reductase subunit, NrdJa | + | + | + | + | + |
| PA5498 | probable adhesin | + | + | + | + | - |
| PA5504 | D-methionine ABC transporter membrane protein | - | - | - | - | + |
| PA5505 | probable TonB-dependent receptor | + | + | + | + | - |
| PA5506 | hypothetical protein | - | - | - | - | + |
| PA5507 | hypothetical protein | + | + | + | + | - |
| PA5508 | Glutamylpolyamine synthetase homologue | - | - | - | - | + |
| PA5509 | hypothetical protein | + | + | + | + | - |
| PA5515 | hypothetical protein | - | - | - | - | + |
| PA5516 | pyridoxamine kinase | + | + | + | + | - |
| PA5520 | hypothetical protein | - | - | - | - | + |
| PA5521 | probable short-chain dehydrogenase | + | + | + | + | - |
| PA5522 | Glutamylpolyamine synthetase | - | - | - | - | + |
| PA5523 | probable aminotransferase | + | + | + | + | - |
| PA5527 | hypothetical protein | - | - | - | - | + |
| PA5528 | hypothetical protein | + | + | + | + | - |
| PA5544 | conserved hypothetical protein | - | - | - | - | + |
| PA5545 | conserved hypothetical protein | + | + | + | + | + |
| PA5546 | conserved hypothetical protein | + | + | + | + | - |
| PA5548 | probable major facilitator superfamily (MFS) transporter | - | - | - | - | + |
| PA5549 | glucosamine--fructose-6-phosphate aminotransferase | + | + | + | + | - |
| PA5551 | hypothetical protein | - | - | - | - | + |
| PA5552 | glucosamine-1-phosphate acetyltransferase/N-acetylglucosamine-1-phosphate uridyltransferase | + | + | + | + | + |
| PA5553 | ATP synthase epsilon chain | + | + | + | + | + |
| PA5554 | ATP synthase beta chain | + | + | + | + | + |
| PA5555 | ATP synthase gamma chain | + | + | + | + | + |
| PA5556 | ATP synthase alpha chain | + | + | + | + | + |
| PA5557 | ATP synthase delta chain | + | + | + | + | + |
| PA5558 | ATP synthase B chain | + | + | + | + | - |
| PA5559 | atp synthase C chain | - | - | - | - | + |
| PA5560 | ATP synthase A chain | + | + | + | + | - |
| PA5561 | ATP synthase protein I | - | - | - | - | + |
| PA5562 | chromosome partitioning protein Spo0J | + | + | + | + | - |
| PA5563 | chromosome partitioning protein Soj | - | - | - | - | + |
| PA5564 | glucose inhibited division protein B | + | + | + | + | - |
| PA5566 | hypothetical protein | - | - | - | - | + |
| PA5567 | conserved hypothetical protein | + | + | + | + | - |
| PA5568 | conserved hypothetical protein | + | - | - | - | - |
| PA5570 | 50S ribosomal protein L34 | - | - | - | - | + |
| NA | Conserved domain protein | + | + | + | + | - |
| NA | Alpha/beta hydrolase fold (EC 3.8.1.5) | + | + | + | + | - |
| NA | FIG006163: hypothetical protein | + | + | + | + | - |
| NA | PhnB protein; putative DNA binding 3-demethylubiquinone-9 3-methyltransferase domain protein | + | + | + | + | - |
| NA | Type III effector HopPmaJ | + | + | + | + | - |
| NA | cytosolic long-chain acyl-CoA thioester hydrolase family protein | + | + | + | + | - |
| NA | FIG00957482: hypothetical protein | + | + | + | + | - |
| NA | Cysteine synthase (EC 2.5.1.47) | + | + | + | + | - |
| NA | Diadenosine tetraphosphate (Ap4A) hydrolase and other HIT family hydrolases | + | + | + | + | - |
| NA | Glutamine synthetase (EC 6.3.1.2) | - | + | + | + | - |
| NA | Aspartokinase (EC 2.7.2.4) | - | + | + | + | - |
| NA | hypothetical protein | - | + | - | + | - |
| NA | UDP-glucose dehydrogenase (EC 1.1.1.22) | - | + | - | + | - |
| NA | UDP-glucose 4-epimerase (EC 5.1.3.2) | - | + | - | + | - |
| NA | putative glutamine synthetase | - | + | - | + | - |
| NA | Flagellin protein FlaC | - | + | - | + | - |
| NA | Phenazine biosynthesis protein PhzE @ 2-Amino-2-deoxy-isochorismate synthase (EC 4.1.3.-) # TrpAa/TrpAb-PhzE type | - | - | - | + | - |
| NA | Serine hydroxymethyltransferase (EC 2.1.2.1) | - | - | - | + | - |
| NA | 3-oxoacyl-[acyl-carrier-protein] synthase, KASIII (EC 2.3.1.41) | - | - | - | + | - |
| NA | Outer membrane porin, OprD family | - | - | - | + | - |
| NA | CRISPR-associated protein, Cse3 family | - | - | - | + | - |
| NA | Outer membrane porin, OprD family | - | - | - | + | - |
| NA | Polyhydroxyalkanoate granule-associated protein PhaF | - | - | - | + | - |
| NA | Xaa-Pro aminopeptidase (EC 3.4.11.9) | - | - | - | + | - |
| NA | Rubredoxin-NAD(+) reductase (EC 1.18.1.1) | + | + | + | - | - |
| NA | Phage T7 exclusion protein | + | - | + | - | - |
| NA | Type I restriction-modification system, DNA-methyltransferase subunit M (EC 2.1.1.72) / Type I restriction-modification system, specificity subunit S (EC 3.1.21.3) | + | - | + | - | - |
| NA | hypothetical protein | + | - | + | - | - |
| NA | Beta-glucosidase (EC 3.2.1.21) | + | - | + | - | - |
| NA | Translation elongation factor Tu | + | - | + | - | - |
| NA | Malonyl CoA-acyl carrier protein transacylase (EC 2.3.1.39) | + | - | + | - | - |
| NA | FIG00960783: hypothetical protein | + | - | + | - | - |
| NA | hypothetical protein | + | - | + | - | - |
| NA | elements of external origin; phage-related functions and prophages | + | - | + | - | - |
| NA | Secreted protein Hcp | + | - | + | - | - |
| NA | Large exoproteins involved in heme utilization or adhesion | + | + | - | - | - |
| NA | Phosphogluconate dehydratase (EC 4.2.1.12) | + | - | - | - | - |
| NA | Exoenzymes regulatory protein AepA precursor | + | - | - | - | - |
| NA | DamX, an inner membrane protein involved in bile resistance | + | - | - | - | - |
| NA | 2-methylaconitate isomerase | + | - | - | - | - |
| NA | COGs COG3777 | + | - | - | - | - |
| NA | Chemotactic transducer | + | - | - | - | - |
| NA | CTP synthase (EC 6.3.4.2) | + | - | - | - | - |
| NA | FIG00956760: hypothetical protein | + | - | - | - | - |
| NA | putative glutamine synthetase | + | - | - | - | - |
| NA | Branched-chain alpha-keto acid dehydrogenase, E1 component, alpha subunit (EC 1.2.4.4) | + | - | - | - | - |
| NA | Phenazine biosynthesis protein PhzF | + | - | - | - | - |
| NA | S-adenosylmethionine synthetase (EC 2.5.1.6) | + | - | - | - | - |
| NA | still frameshift probable component of chemotactic signal transduction system | + | - | - | - | - |
| NA | Alcohol dehydrogenase (EC 1.1.1.1) | + | - | - | - | - |
| NA | Phosphoadenylyl-sulfate reductase [thioredoxin] (EC 1.8.4.8) / Adenylyl-sulfate reductase [thioredoxin] (EC 1.8.4.10) | + | - | - | - | - |
| NA | TPR repeat containing exported protein; Putative periplasmic protein contains a protein prenylyltransferase domain | + | - | - | - | - |
| NA | Dihydroaeruginoate synthetase PchE, non-ribosomal peptide synthetase modules | - | + | + | - | - |
| NA | COG1629: Outer membrane receptor proteins, mostly Fe transport | - | - | + | - | - |
| NA | Alginate o-acetyltransferase AlgF | - | - | + | - | - |
| NA | Inosine-5'-monophosphate dehydrogenase (EC 1.1.1.205) | - | - | + | - | - |
| NA | WbjC | - | - | + | - | - |
| NA | UDP-N-acetylglucosamine 4,6-dehydratase (EC 4.2.1.-) | - | - | + | - | - |
| NA | Cyclohexadienyl dehydrogenase (EC 1.3.1.12)(EC 1.3.1.43) / 5-Enolpyruvylshikimate-3-phosphate synthase (EC 2.5.1.19) # TyrAc/AroF, NAD-specific | - | - | + | - | - |
| NA | formate dehydrogenase formation protein FdhE | - | - | + | - | - |
| NA | hypothetical protein | - | - | + | - | - |
| NA | Chemotaxis response regulator protein-glutamate methylesterase CheB (EC 3.1.1.61) | - | - | + | - | - |
| NA | Transcriptional regulatory protein PhoP | - | + | - | - | - |
| NA | Molybdopterin biosynthesis protein MoeA | - | + | - | - | - |
| NA | Membrane alanine aminopeptidase N (EC 3.4.11.2) | - | + | - | - | - |
| NA | Dipeptide-binding ABC transporter, periplasmic substrate-binding component (TC 3.A.1.5.2) | - | + | - | - | - |
| NA | ATP-dependent protease La (EC 3.4.21.53) Type II | - | + | - | - | - |
| NA | hypothetical protein | - | + | - | - | - |
| NA | hypothetical protein | - | + | - | - | - |
| NA | Phenazine biosynthesis protein PhzA | - | + | - | - | - |
| NA | Nicotinamidase family protein YcaC | - | + | - | - | - |
| NA | CRISPR-associated protein, Csy3 family | - | + | - | - | - |
| NA | CRISPR-associated protein, Csy4 family | - | + | - | - | - |
| NA | Low-specificity L-threonine aldolase (EC 4.1.2.5) | - | + | - | - | - |
| NA | Histone acetyltransferase HPA2 and related acetyltransferases | - | + | - | - | - |
| NA | tRNA pseudouridine 13 synthase (EC 4.2.1.-) | - | + | - | - | - |
| NA | Poly (beta-D-mannuronate) C5 epimerase precursor (EC 5.1.3.-) | - | + | - | - | - |
